# Lipid Profiling of Alzheimer’s Disease Brain Highlights Enrichment in Glycerol(phospho)lipid, and Sphingolipid Metabolism

**DOI:** 10.3390/cells10102591

**Published:** 2021-09-29

**Authors:** Sumeyya Akyol, Zafer Ugur, Ali Yilmaz, Ilyas Ustun, Santosh Kapil Kumar Gorti, Kyungjoon Oh, Bernadette McGuinness, Peter Passmore, Patrick G. Kehoe, Michael E. Maddens, Brian D. Green, Stewart F. Graham

**Affiliations:** 1 Metabolomics Department, Beaumont Research Institute, Beaumont Health, Royal Oak, MI 48073, USA; sumeyyaak@gmail.com (S.A.); zaferugur34@gmail.com (Z.U.); ali.yilmaz@beaumont.org (A.Y.); kjohmd@gmail.com (K.O.); 2 William Beaumont School of Medicine, Oakland University, Rochester, MI 48073, USA; 3 College of Computing and Digital Media, DePaul University, Chicago, IL 60604, USA; IUSTUN@depaul.edu (I.U.); Michael.Maddens@beaumont.org (M.E.M.); 4 SCIEX, 500 Old Connecticut, Framingham, MA 01701, USA; Santosh.Kapil@sciex.com; 5 Department of Obstetrics and Gynecology, Seoul National University Bundang Hospital, 82, Gumi-ro 173 Beon-gil, Bundang-gu, Seongnam-si 13620, Gyeonggi-do, Korea; 6 Centre for Public Health, School of Medicine, Dentistry and Biomedical Sciences, Queen’s University Belfast, Belfast BT12 6BA, UK; B.McGuinness@qub.ac.uk (B.M.); p.passmore@qub.ac.uk (P.P.); 7 Dementia Research Group, Translational Health Sciences, Bristol Medical School, University of Bristol, Bristol BS10 5NB, UK; patrick.kehoe@bristol.ac.uk; 8 Institute for Global Food Security, School of Biological Sciences, Queen’s University Belfast, Belfast BT9 5DL, UK; b.green@qub.ac.uk

**Keywords:** Alzheimer’s disease, brain, metabolomics, lipidomics, pathogenesis

## Abstract

Alzheimer’s disease (AD) is reported to be closely linked with abnormal lipid metabolism. To gain a more comprehensive understanding of what causes AD and its subsequent development, we profiled the lipidome of postmortem (PM) human brains (neocortex) of people with a range of AD pathology (Braak 0–6). Using high-resolution mass spectrometry, we employed a semi-targeted, fully quantitative lipidomics profiling method (Lipidyzer) to compare the biochemical profiles of brain tissues from persons with mild AD (n = 15) and severe AD (AD; n = 16), and compared them with age-matched, cognitively normal controls (n = 16). Univariate analysis revealed that the concentrations of 420 lipid metabolites significantly (*p* < 0.05; q < 0.05) differed between AD and controls. A total of 49 lipid metabolites differed between mild AD and controls, and 439 differed between severe AD and mild AD. Interestingly, 13 different subclasses of lipids were significantly perturbed, including neutral lipids, glycerolipids, glycerophospholipids, and sphingolipids. Diacylglycerol (DAG) (14:0/14:0), triacylglycerol (TAG) (58:10/FA20:5), and TAG (48:4/FA18:3) were the most notably altered lipids when AD and control brains were compared (*p* < 0.05). When we compare mild AD and control brains, phosphatidylethanolamine (PE) (*p*-18:0/18:1), phosphatidylserine (PS) (18:1/18:2), and PS (14:0/22:6) differed the most (*p* < 0.05). PE (*p*-18:0/18:1), DAG (14:0/14:0), and PS (18:1/20:4) were identified as the most significantly perturbed lipids when AD and mild AD brains were compared (*p* < 0.05). Our analysis provides the most extensive lipid profiling yet undertaken in AD brain tissue and reveals the cumulative perturbation of several lipid pathways with progressive disease pathology. Lipidomics has considerable potential for studying AD etiology and identifying early diagnostic biomarkers.

## 1. Introduction

Alzheimer’s disease (AD) is the most common cause of progressive and degenerative dementia and accounts for about two-thirds of dementia cases [1]. It is estimated that the total number of people with AD in the United States in 2020 was 5.8 million, and this is projected to rise to 13.8 million in 2050 [2]. The estimated healthcare costs for AD in the United States is estimated to have been USD 305 billion in 2020, and this is expected to rise to USD 1 trillion in 2050 [3] if the current lack of disease-modifying therapies continues.

Current AD diagnosis is predominantly based on clinical symptoms characterized by the onset and progressive impairment of memory and other cognitive functions allied to reduction of daily living activities [4]. The accumulation of β-amyloid (Aβ) plaques and neurofibrillary tangles are considered classical hallmarks of AD pathology [5]. However, the actual biochemical basis for neurodegeneration is poorly understood. A growing body of evidence shows that the pathological processes may begin 20–30 years before the diagnosis of clinical AD [6,7], including that of cerebrovascular dysfunction, and the only truly definitive means of confirming diagnosis is via autopsy. As such, great efforts continue to identify accurate biomarkers for the early diagnosis of the disease before irreversible neurodegeneration has occurred and when therapies are most likely to be effective [8].

In 2011, the National Institute on Aging and the Alzheimer’s Association (NIA-AA) joint workgroups proposed a revised diagnostic criterion for AD [9]. They recommended (1) separating the disease into three clinical stages (preclinical, mild cognitive impairment (MCI), and Alzheimer’s dementia), and (2) incorporating biomarkers, such as cerebrospinal fluid (CSF) analysis, structural and molecular imaging, and genetic mutation analysis into diagnostic formulations for probable and possible AD dementia. Recently, the NIA-AA research framework defined AD by its underlying pathologic processes that can be documented by in vivo biomarkers (imaging and biofluid) regardless of the presence or absence of clinical symptoms [10]. Although the criteria are not intended for clinical practice, they create a scheme for defining and staging the disease across its entire spectrum by the incorporation of in vivo biomarkers with the previously established neuropathologic definition of AD [11].

The changes in the lipidome and how they relate to AD remain poorly understood. However, alterations in lipid metabolism have been reported to play an important role in AD pathogenesis [12]. Lipidomics is considered one of the newer ‘omics’ techniques which enables the comprehensive characterization of thousands of lipids [13]. Recent lipidomics studies have proposed that phospholipids and sphingolipids (SL) play a role in AD development [14]. Our group have previously reported substantial differences in fatty acid concentration in postmortem (PM) brain tissue in people who died from AD as compared with cognitively healthy controls [15,16]. In another study, Zangh et al. (2020) noted the significant perturbation of lipids, such as sterol lipids, phospholipids, and fatty acids, in the brain tissue of APP/PS1 transgenic mice, and identified nine lipids as a potential biomarker panel for the diagnosis of AD [17]. Barupal et al. (2019) reported that acylcarnitines, fatty acids, sterol lipids, phospholipids, sphingolipids, and acylglycerols are significantly perturbed in AD. They describe 168 lipids which are at significantly different concentrations in the blood of AD patients [18]. Wood et al. (2015) also detail elevations in monoacylglycerols and diacylglycerols in gray matter from subjects with MCI. Further, they also highlight low levels of docosahexaenoic acid (DHA) in the cerebrospinal fluid and gray matter, suggesting that the DHA transport metabolism was altered in AD [19].

In this present study, we applied an expansive and quantitative lipidomic methodology to compare post-mortem human brain tissue from cases of mild AD and AD, with that of age-matched, cognitively normal control subjects. The principal objectives were to identify lipids that can function as biomarkers associated with the development and progression (severity) of the disease, and to gain insights into the changing biochemical environment underlying AD development in brain.

## 2. Materials and Methods

### 2.1. Tissue Samples

Lipidomics studies were performed on: human PM brain tissue (neocortex) obtained from people with no medical history of cognitive impairment and the absence of significant pathology normally commensurate with a disease diagnosis (Braak stage 0–2; n = 16); human PM brain tissue (neocortex) obtained from people with mild AD, representing subjects with a history of cognitive impairment consistent with dementia and mild AD pathology (Braak stage 3–4; n = 15); and human PM brain tissue (neocortex) obtained from people with severe AD (AD) patients (Braak stage 5–6; n = 16), representing an advanced pathology related to end-stage disease.

Table S1 of the Supplementary Materials lists the patient demographics, including their Braak stage and PM interval (PMI) in addition to all available clinical characteristics. Tissue samples were obtained through the Brains for Dementia Research (BDR) initiative (https://bdr.alzheimersresearchuk.org/, accessed on September 2014), a brain bank network funded by Alzheimer’s Brain Bank UK (ABBUK), a charity co-funded by Alzheimer’s Research UK (ARUK) and the Alzheimer’s Society. The neuropathological diagnoses were made using widely accepted criteria [20,21] and uniformly applied according to a standardized protocol by members of the BDR neuropathology group. Consent and ethical approval for the use of tissue was obtained by the individual brain banks, all of which are licensed by the Human Tissue Authority. Further, all protocols were approved by the Beaumont Institutional Review Board (HIC#20017-017).

### 2.2. Chemicals and Internal Standards

LC-MS grade dichloromethane, methanol, isopropyl alcohol, and ethanol were obtained from Fisher Scientific (Pittsburgh, PA, USA). Ammonium acetate was purchased from Millipore Sigma Sigma-Aldrich (St. Louis, MO, USA). EquiSPLASH Lipidomics Mass Spec Internal standards were purchased from Avanti Polar Lipids (Alabaster, AL, USA) and are listed in the Supplementary Materials, Table S2.

### 2.3. Sample Preparation

25 mg (±0.1 mg) of lyophilized post-mortem brain tissue was dissolved in a mixture of 1 mL of MiliQ H_2_O, 2.0 mL of methanol (MeOH), and 0.9 mL of dichloromethane (CH_2_Cl_2_), and the sample was vortexed for 10 s. Two distinct phases were observed when the samples were allowed to equilibrate. We subsequently added an additional 50 µL of MeOH to achieve a mono phase extract, to which we added 20 µL of the EquiSPLASH internal standard. Spiked PM brain samples were vortexed for 10 s and kept at room temperature for 30 min. Samples were extracted with the addition of 1 mL of MiliQ H_2_O and 0.9 mL CH_2_Cl_2_, and inverted 10 times. The distinctive lower layer was collected following centrifugation at 1200× *g* at room temperature for 10 min. Any remaining lipids were further extracted from the initial extract using 2 mL of dichloromethane and subsequently combined with the preceding extract. The supernatant was dried overnight in a fume hood and the sample was subsequently reconstituted in 50 µL of ethanol prior to analysis.

### 2.4. LC-MS/MS Analysis

A Shimadzu Exion liquid chromatography unit coupled with a Sciex QTRAP 6500+ mass spectrometer (MS) was used for the separation and detection of all lipids in the PM brain tissue extracts with polarity switching. We injected 5 µL of extract onto a Waters XBridge Amide (3.5 µm, 4.6 × 150 mm) HILIC column (maintained at 35 °C) and the optimal chromatographic separation was achieved at a flow rate of 0.7 mL/min using a gradient with solvent A (1 mM ammonium acetate in 95% acetonitrile containing pH~8.2) and solvent B (1 mM ammonium acetate in 50% acetonitrile 50% MiliQ-H2O pH~8.2) as follows: initial conditions were 100% solvent A; 0–6.0 min, 6% Solvent B; 6–10 min, 25% Solvent B; 10–11 min, 98% Solvent B; 11–13 min, 100% Solvent B; 13–18 min, 100% Solvent B; 18–24 min, 0% Solvent B. Retention times were initially determined using spiked internal standards or a representative matrix sample and the unscheduled MRM method. The ion source parameters were optimal at curtain gas 30 psi, nebulizer gas 50 psi, auxiliary gas 60 psi, 5500V ion spray voltage and at 500 °C. A scheduled multiple reaction monitoring (MRM) transition method was used to quantify over 1200 lipids. Details on the chromatographic and MS methods can be obtained from the Comprehensive Targeted Method for Global Lipidomics Screening. All data was processed using MultiQuant™ Software 3.0.2. Automated computation of the time scheduled final MRM methods was performed using the sMRM Pro Builder.

### 2.5. Statistical Analysis

#### 2.5.1. Data Quality Control

All data handling, statistical analysis, and machine learning (ML) model building steps were completed with Python using numpy, pandas, and sklearn packages [22,23]. Prior to statistical analysis, preprocessing steps (cleaning, transformation, and imputation of missing values) were undertaken in order to vigorously prepare the data. In brief, variables were omitted if they had >50% missing values for a given analyte. Subsequently, data were normalized using the sum to one normalization algorithm, which aims to reduce sample-to-sample variation due to any potential dilution effect. The resultant data were multiplied by a factor of 1e106 to optimize the effect of small values. Normalized data were subjected to generalized log (glog) transformation [24] and auto-scaling (each variable is centered by the mean and scaled by the sample standard deviation using n-1 degrees of freedom). Finally, missing measurements were imputed using K-nearest neighbors with K equal to 3.

#### 2.5.2. Univariate and One-Way ANOVA Analysis

All demographic information was analyzed using a one-way Analysis of Variance (ANOVA) in IBM SPSS Statistics toolbox (version 24.0, Chicago, IL, USA. Three pairwise comparisons (control vs. mild AD; control vs. AD; mild AD vs. AD) were performed on only the lipidomics data. All data were tested for normality. The false discovery rate (FDR, q-values) was also calculated to take into consideration the corrections needed for multiple comparisons. For lipids showing normal distribution, *p*-values were calculated with Student’s *t*-test as a default, and for lipids which were non-normally distributed, their *p*-values were calculated using the non-parametric Wilcoxon–Mann–Whitney U test.

#### 2.5.3. Machine Learning Models

Preprocessed data were analyzed in subsets of two classes where the class was the target variable. Feature selection was performed using the recursive feature elimination (RFE) method with logistic regression as the classifier. RFE recursively removes attributes and builds a model on the remaining traits. It uses the model accuracy to identify and rank which of the attributes contribute the most to predicting the target characteristic. The highest-ranked single attribute was chosen as an input variable to identify the target group value in the binary-class classification analysis. To prevent overfitting when too many variables are used and there are not enough observations, a single attribute was chosen. For each model, a five-fold cross validation was performed for parameter optimization [25,26]. In an attempt to systematically investigate the predictive performance of the various machine learning approaches, several models were tested. The models included logistic regression, linear discriminant analysis, quadratic discriminant analysis, Gaussian naïve Bayes, linear support vector machine (SVM), Gaussian kernel SVM, K-nearest neighbors, decision trees, random forest, gradient-boosted machine, and XGBoost. After the models were trained and the parameters were optimized, each model’s accuracy has been obtained by three-fold cross-validation with 10 repeats. In addition, sensitivity, specificity, F1-scores, and AUC metrics have been calculated. When considering the AUC performance measures, it can be expected that the random forest and gradient boosting yield had a very good performance at extreme levels of class imbalance. The statistical significance of each model has been further assessed by permutation testing (*p* < 0.05).

## 3. Results

The results from corresponding multi-group comparison tests through one-way ANOVA showed that age, gender, and PMI were not statistically different across the three diagnostic groups. This indicates that pathology (i.e., Braak stage) scoring of each participant can be confidently associated with the measured lipid profiles (Table 1).

### 3.1. Principal Component Analysis (PCA)

**Figure 1:**
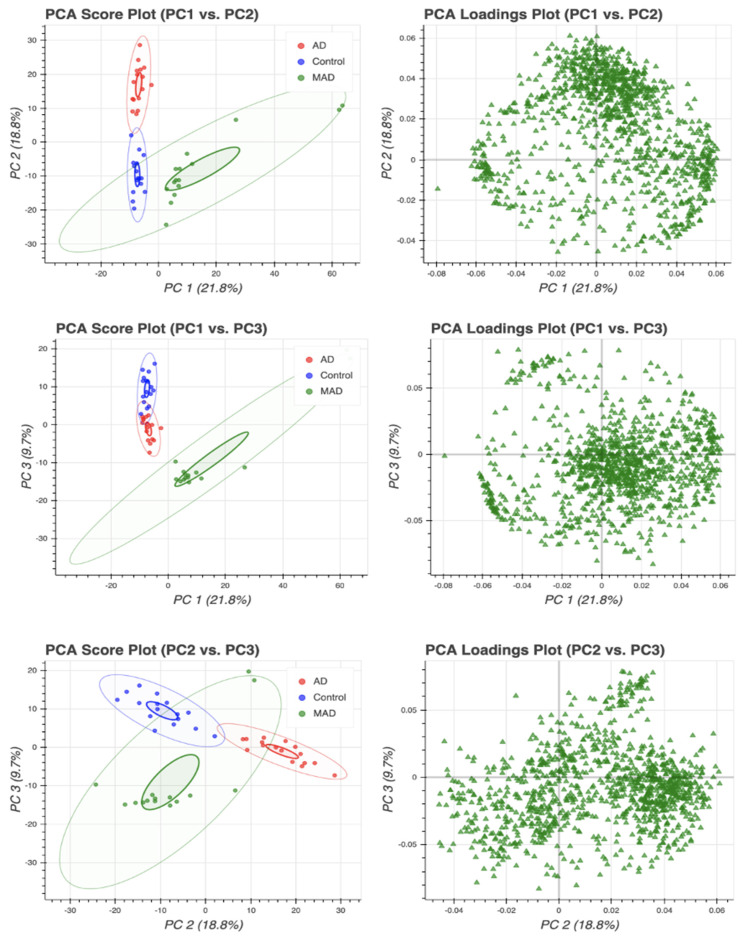
Principal component analysis (PCA) of the imputed and transformed data for outlier detection in control, mild AD, and AD groups was performed to check for any intrinsic variation. The following steps were followed to handle the data: standardization of the data; computing the covariance matrix; calculating the eigenvectors and eigenvalues; computing the principal components; and reducing the dimensions of the dataset.

Prior to performing univariate and subsequent machine learning statistics, principal component analysis (PCA) was performed (Figure 1) on the complete lipidomic dataset to check for any intrinsic variation and to remove any potential outliers (i.e., data points outside the 95% confidence interval). One sample in the AD group was an outlier and was excluded. Among the 1143 lipid species detected, only 500 were used for further statistical analysis, as the remainder were not measured consistently in all three technical repeats. Figure S1 in the Supplementary Materials displays the heat map combining the complete dataset for all brain tissues studied, highlighting the increasing concentration of the majority of lipids from Braak stage 0 (controls) through to Braak stage 6 (AD). Subsequent univariate analysis revealed that of the quantified lipids, when comparing the mild AD and control groups, 49 lipids showed significant differences (*p* < 0.05; q < 0.05) (Supplementary Materials, Table S3). When comparing mild AD and AD, of the 500 lipids measured, the levels of 439 in these groups were statistically significantly different (*p* < 0.05; q < 0.05) (Supplementary Materials, Table S4). Lastly, when we compared healthy controls with AD, 420 lipids were found to be at significantly different concentrations (*p* < 0.05; q < 0.05) (Supplementary Materials, Table S5). According to our findings, the most notably perturbed individual lipids when comparing AD and controls were diacylglycerol (DAG) (14:0/14:0), triacylglycerol (TAG) (58:10/FA20:5), and TAG (48:4/FA18:3), when comparing mild AD and controls were phosphatidylethanolamine (PE) (*p*-18:0/18:1), phosphatidylserine (PS) (18:1/18:2), and PS (14:0/22:6), and when comparing AD and mild AD, the most notably perturbed were PE (*p*-18:0/18:1), DAG (14:0/14:0), and PS (18:1/20:4).

### 3.2. Overall Lipid Subclass Changes between the Groups

**Figure 2:**
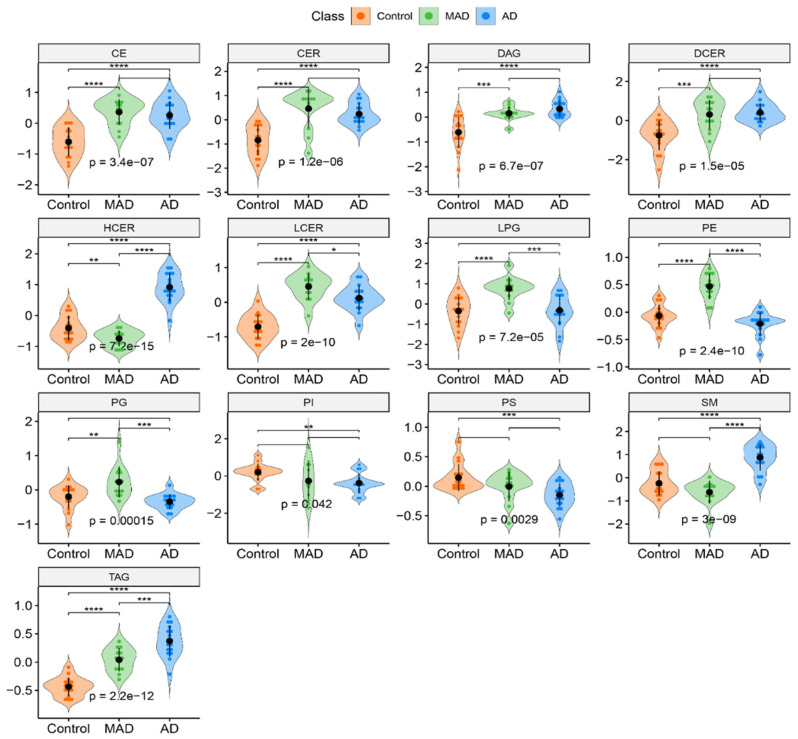
Violin plots comparing the median concentration of different total species of complex lipids in the brain among AD (n = 16), mild AD (n = 15), and control (n = 16) groups. Data was analyzed by *t*-test, where: ns, *p* > 5 × 10^−2^; * *p* < = 5 × 10^−2^; ** *p* < = 1 × 10^−2^; *** *p* < = 1 × 10^−3^; **** *p* < = 1 × 10^−4^ Abbreviations: CE, cholesteryl esters; CER, ceramides; DAG, diacylglycerols; DCER, dihydroceramides; HCER, hexosylceramides; LCER, lactosylceramides; LPG, lysyl-phosphatidylglycerol; MAD, Mild AD (Mild Alzheimer’s disease); PE, phosphatidylethanolamine; PG, phosphatidylglycerol; PI, phosphatidylinositol; PS, phosphatidylserine; SM, sphingomyelins; TAG, triacylglycerols.

Given the large number of measured lipids, we investigated overall concentration changes of lipid subclasses between groups (instead of individual lipids) to determine if any significant perturbation in the lipidome was a consequence of the Braak stage (Figure 2). Apart from a few exceptions, the violin plots highlight elevated levels of lipid classes in mild AD cases compared to cognitively healthy controls (*p* < 0.05; q < 0.05). Those lipid groups include cholesteryl esters (CE), ceramides (CER), diacylglycerols (DAG), dihydroceramides (DCER), lactosylceramides (LCER), lysyl-phosphatidylglycerol (LPG), phosphatidylethanolamine (PE), phosphatidylglycerol (PG) and triacylglycerols (TAG). Among the identified lipids CE, CER, DAG, DCER, LCER, and LPG showed levels very similar to those observed in the control brain profiles. In this pairwise comparison, hexosylceramides (HCER) were the only class of lipids found to be at a higher concentration in cognitively healthy controls as compared to mild AD. In contrast, when comparing AD vs. mild AD/control, HCER was identified as being at significantly higher concentrations in AD brains (*p* < 0.05), showing that it is a unique compound possibly capable of measuring disease severity. Phosphatidylinositol (PI), phosphatidylserine (PS), and sphingomyelins (SM) did not differ between any groups.

When comparing mild AD and AD, SM and TAG, as well as HCER, as already mentioned, showed statistically significant elevated levels of concentration (*p* < 0.05; q < 0.05), whilst LCER, LPG, PE, and PG were found to be significantly decreased in AD cases (*p* < 0.05; q < 0.05). Notably, TAG concentrations appeared to have a stepwise increase from control to mild AD to AD. In the summary of the 13 lipid classes, 10 were found to have significantly different lipid concentrations (*p* < 0.05; q < 0.05) between controls and AD, eight of which (CE, CER, DAG, DCER, HCER, LCER, SM, and TAG) were at decreased concentrations in controls, and two (PI and PS) were at higher concentrations in AD PM brains.

### 3.3. Diagnostic Performance of the Machine Learning Model

The diagnostic performance of each machine learning model when classifying control vs mild AD, mild AD vs AD, and control vs AD all demonstrated excellent predictive performances with ~90–100% accuracy, highlighting the potential of lipids as potential diagnostic tools for dementia. For example, using the concentrations of the measured lipids, we evaluated performance of several ML algorithms based on their classification accuracy, sensitivity and specificity, and area under curve (AUC) values. As highlighted in Table S6 of the Supplementary Materials, the top three ML classification algorithms for differentiating mild AD from controls using the concentration of PC (14:0/18:1) were logistic regression, gbm, and xgboots, with sensitivity values of 87%, 100%, and 93%, and specificity of 81%, 81%, and 88%, respectively. The AUC values of these top models, using RFE-selected features, were 91%, 92%, and 91%, respectively.

The same procedure was repeated with PC (18:0/18:1) as identified using the RFE method. The top three models accurately distinguishing mild AD from AD were found to be random forest, xgboost, and gbm, with sensitivity–specificity values of 100–80%, 94–88%, and 94–87% at a 95% confidence interval, respectively. In this instance, all three models provided AUC values of 94%, 92%, and 94, respectively. (Supplementary Materials, Table S7).

We identified the sphingomyelin SM (22:0) as the lipid that explains the greatest amount of variation associated with the separation of cognitively healthy samples from AD. For this pairwise comparison, we identified random forest, gbm, and xgboost as the greatest classification algorithms providing sensitivity–specificity values of 95–89%, 94–90%, and 93–86%, respectively (Supplementary Materials, Table S8). The corresponding AUC values for said model were found to be 99%, 88%, and 89%, respectively.

## 4. Discussion

To investigate the major perturbations in the AD brain lipidome, we employed an unbiased, quantitative, global lipidomics approach to profile PM brain from AD cases with a range of AD pathologies and compared them with controls. The results highlight significant cumulative differences in brain lipid biochemistry following AD onset. To the best of our knowledge, no previously reported studies have used quantitative lipidomics to examine the severity of AD brain pathology.

Previous animal and human studies have suggested that AD pathophysiology is directly linked to perturbed lipid metabolism [27,28]. Our study highlights several subclasses of lipids that are linked with the increasing severity of AD pathology, including: (i) neutral lipids (CE); (ii) glycerolipids (DAG and TAG); (iii) glycerophospholipids (GP) (PE, PG, phosphatidylinositol (PI), PS, and LPG); and (iv) sphingolipids (SL) (SM, CER, DCER, HCER, and LCER).

**Figure 3:**
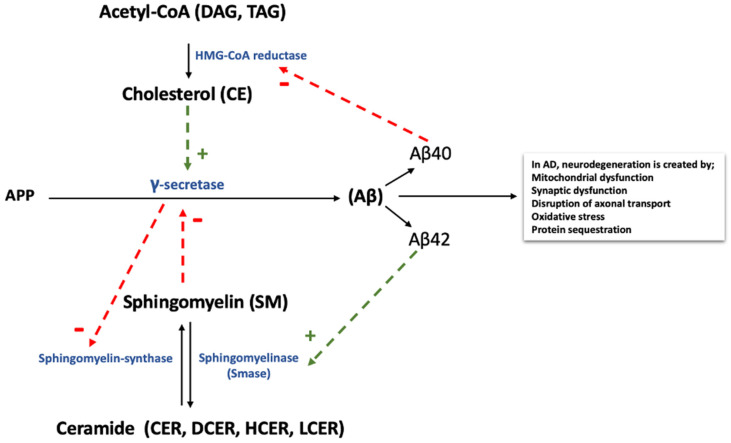
Proposed feedback modulation of Aβ synthesized from APP by the enzyme γ-secretase. Two main lipid groups, cholesterol and sphingomyelin, are closely related to Aβ production in AD. Cholesterol increases γ-secretase activity, which leads to elevated Aβ production. Conversely, Aβ downregulates cholesterol de novo synthesis from acetyl-CoA by inhibiting enzyme HMG-CoA reductase. Sphingomyelin inhibits γ-secretase activity, and in return reduced γ-secretase activity depresses sphingomyelin synthase activity. (Figure modified from the manuscript Zinser E.G. et al. BBA 2007;1768:1991–2001.) The related lipid species we measured are listed in the parentheses. Acetyl-CoA synthesized from fatty acids is released from diacylglycerol and triacylglycerols. Abbreviations: APP, amyloid precursor protein; CE, cholesteryl esters; CER, ceramides; DAG, diacylglycerols; DCER, dihydroceramides; HCER, hexosylceramides; LCER, lactosylceramides; SM, sphingomyelins; TAG, triacylglycerols.

The link between AD and cholesterol has long been established, but it is not clear how cholesterol influences AD pathology. It is known that cholesterol combines with SL on the cell membrane to form lipid rafts that are resistant to detergents [29]. It has also been shown that brain cholesterol can directly affect Aβ-related processing (Figure 3). Aβ oligomerization has been found to be reduced by decreased cholesterol levels in hippocampal neurons [30], while γ- and β-secretase activities were promoted due to elevated cholesterol levels in AD brains [31]. Taken together, in the AD affected brain, cholesterol increases γ-secretase and BACE1′s ability to cleave APP (Figure 3) and produce increased levels of Aβ. Our data demonstrate that both AD and mild AD groups have increased CE levels compared with controls, with 15 perturbed in AD, and two in mild AD (Supplementary Materials, Tables S3 and S5). Brain cholesterol levels predominantly depend on de novo synthesis within astrocytes, oligodendrocytes, and (to a lesser extent) neurons [32]. Cholesterol and its links with AD pathogenesis have been studied in depth, with the genetic variant APOE ε4 of apolipoprotein E (ApoE) being the strongest genetic risk factor for late-onset AD (LOAD) [33]. ApoE is the main carrier protein for cholesterol in the brain, and helps cholesterol transfer from the astrocytes to the neurons. In addition to lipid transport, ApoE also has the capacity to bind Aβ in a genotype-dependent manner and contribute to differences in levels of abnormal protein aggregation [34]. The ε4 allele is linked to increased accumulation and deposition of Aβ in the brain and the cerebrovascular system [35], and increased tau tangles [36]. Despite the fact that not all APOE ε4 carriers will develop LOAD, studies have shown that people with altered cholesterol and SL metabolism may develop AD as a result, while APOE ε4 carriers do not [37]. Our results indicate that the aforementioned functions may be dysregulated in AD due to perturbed CE levels. It has been previously reported that both higher and lower levels of brain cholesterol may be a direct contributor to AD pathogenesis, as cholesterol is important for the production of tau tangles and Aβ plaques [38].

Our data highlight increased levels of DAG (FA18:1) and TAG lipid species in both AD and mild AD PM cohorts when compared with controls. There were also significant perturbations in the concentration of GPs and SLs in both AD and mild AD sufferers. Glycerophospholipids and SL are important not only for the neuronal membrane configurational integrity, but also as pioneers of biologically active brain lipid mediators [39]. Changes in lipid remodeling affect signal transduction [40]. The combined loss of myelin PE and sulfatides further reflects a complex and progressive hypomyelination in AD. As a member of SL, CER are thought to be involved in the activation of cell death pathways and neuroinflammation [41], both known contributors to complex AD pathology.

Our study highlights specific changes in GP metabolism, previously associated with the development of AD. We report 15 and 27 GPs, respectively, that significantly differ in AD and mild AD compared with control (Supplementary Materials, Tables S3 and S5). Previous studies have reported that AD brain tissue has fluctuating levels of phospholipids, including PE and PI [42]. Glycerophospholipid is an amphiphilic molecule that plays an active role in ion channel function, transporters, receptors, and regulation of neuronal membrane function, transport, and proliferation. Brain glycerophospholipids are reportedly lower whenever there is AD pathology [43] and it might be surmised that this is a result of increased phospholipid degradation [44]. In general, lower GP concentrations have been linked with increased neurofibrillary tangle formation and amyloid pathology. Subgroups of the PG family, namely PI, PE, and PC, are significantly reduced in the neuronal membrane of AD patients as compared to age- and gender-matched controls [45]. This reduction causes changes in membrane permeability and fluidity, as well as homeostasis of ions, which in turn increases oxidative stress.

Many GP degradation products are known to be proinflammatory, involved in the direct activation of microglia and astrocytes, leading to the secretion of inflammatory cytokines [46], which consequently contributes to Aβ development. These cytokines further enhance neuroinflammation and oxidative stress [46]. In this study, some changes were observed in lipids related to the Aβ clearance pathway. The reduction of CSF Aβ1-42 peptide markers was proposed to indicate that the peptide was associated with less clearance from the brain, resulting in accumulation [18]. In the study by Barupal et al. (2019), poor removal of Aβ was demonstrated due to the negative relationship between various different lipids, including polyunsaturated fatty acids (PUFA), TGs, choline plasmalogens, CER, lysophosphatidylcholine (lysoPCs), and PI. Conjugated lipids, especially lysoPC, PI, and CER, are associated with cellular death and may also lead to neuronal toxicity driven by Aβ aggregation [47]. Increased levels of CER with oleic acid (C18:1) has been shown to increase the risk of developing AD [48]. We confirmed this result in the present study and also noticed that lower levels of PI containing PUFAs are correlated with Aβ accumulation.

During the initial stages of AD, PE accounts for approximately 40% of all glycerophospholipids, and this level remains consistent throughout the pathophysiology of the disease. As AD progresses, PE levels decrease by 10% to 30% in gray matter, suggesting that PEs are one of the earliest phospholipids to be affected with the onset of disease [49]. Our data highlight the variability of various lipids (including PE), particularly with respect to the pathological stage of AD.

Biologically active SLs include CER-1-phosphate, sphingosine-1–phosphate (S1P), SM, CER, and others. Sphingolipids play a key role in synaptic transmission and stability, neuronal survival, and signaling pathways by occupying an important part in the myelin layer. All SLs are connected in the phospholipid bilayer by CER, and, in addition to cholesterol, they also represent the main constituent of the lipid raft membrane microdomains [50]. SLs have previously been associated with lysosomal storage disease, which is similar to the mechanism which leads to the development of Aβ and tau pathologies [51]. Perhaps our most notable finding was that SL metabolism in AD and mild AD was severely reduced compared to other lipid groups. The observed changes of several SLs in the AD groups in this study, and their potential to regulate Aβ aggregation and APP processing, supports the association and hypothesis that AD pathogenesis is directly linked with SL metabolism [14]. Our findings are favorably supported by evidence from the literature. Several reports, including PM studies, indicate higher CER levels in CSF from AD [52] and in the cerebral cortical area [41]. White matter CER was reported to be three-fold higher in the cerebellum and temporal cortex of early-stage AD [53]. In the later-stage AD, CER was approximately twice that of the age-matched control group. However, in other studies, gray matter CER remained consistent across all stages of AD [53]. In vitro studies have shown that Aβ stimulates the hydrolysis of SM and causes CER assembly [41,52,54], and CER in turn affects the production of Aβ [55]. The SL pathway is composed of several biologically active molecules with key signaling capabilities, including S1P, sphingosine, thiolipids, CER, and SM [56]. Sphingomyelin can be hydrolyzed by the acidic sphingomyelinase to obtain CER. Subsequently, CER produces sphingosine through ceramidase, and is finally phosphorylated to generate S1P [56]. In this regard we have identified 21 significantly perturbed SLs in AD and four in mild AD compared with control (Supplementary Materials, Tables S3 and S5).

In addition to being directly involved in cell differentiation and proliferation [57], CER is also responsible for promoting and inducing apoptosis [58] and neurotoxic signaling molecules. Given the role of CER in regulating apoptosis and cell survival, imbalances in CER metabolism do have important consequences. As one of the most effective biologically active SLs, it has been reported that CER levels are elevated in various brain regions and CSF in AD patients. Because elevated CER levels occur before clinically recognized stages of AD, they may be related to the etiopathophysiology of the disease [52,53,59] which is supported by longitudinal increases in serum CER concentrations and the increased risk of AD [56]. Disorders of multiple gene expression can explain the accumulation of CER in AD-affected individuals [59]. Katsel et al. (2007) report elevated expression of the genes required for de novo CER synthesis in the brain, while the expression of genes involved in glycosphingolipid formation in CER were reduced. Aβ-mediated activation of sphingomyelinases (SMases) that break down SM to CER may also explain increased CER concentrations in AD brain tissue. Grimm et al. (2005) report that Aβ-peptide directly stimulates neutral SMase activity [60] while Malaplate-Armand et al. (2006) report that Aβ stimulates acidic SMase activity [61]. Increased CER levels and the upregulation of SMases in PM AD brains [49] indicate that the concentration of SM in these tissues may be reduced, however the reported findings to-date are inconsistent [37,41,54]. Additionally, SM levels have been reported to be significantly elevated in the CSF of patients diagnosed with prodromal AD, while SM levels in patients with mild AD were lower but not significantly different [62]. A study by Mielke et al. (2011) [56] reported increased plasma SM concentrations and increased SM/CER ratios in AD patients which were associated with delayed disease progression. Our results also support the findings of the above studies. Gangliosides are predominantly degraded to CER by sequentially removing sugar moieties in the oligosaccharide groups. We report significant changes in CER concentrations in AD affected brains which may also reflect ganglioside metabolism. Their importance is highlighted by the fact that insufficient gangliosides in the CNS can cause degenerative changes [63].

The violin plot visualizations (Figure 2) compare the median concentrations of each lipid class (aggregates of all measured species), highlighting some extremely important differences. Of the 13 lipid classes, seven were found to be significantly perturbed. The mean HCER, SM, and TAG concentrations were found to be significantly increased, and mean LCER, LPG, PE, and PG concentrations were found to be significantly decreased in AD compared with mild AD. This finding suggests that the aforementioned lipid species could be directly linked to the dramatic structural changes observed in the brain when people phenoconvert from mild AD (Braak stage 3–4) to AD (Braak Stage 5–6).

AD is a progressive disease, and as both symptoms and pathology associated with dementia gradually worsen with time, the need for biomarkers to diagnose early-stage dementia caused by AD (or mild AD pathology) is of great importance. This is particularly so for the development of novel disease-modifying treatments which need to target early-stage dementia caused by AD [64,65]. Further investigations of the disease-induced remodeling of brain lipids have considerable potential to transform diagnosis and treatment.

It should be noted that variability of the proportions of white and gray matter in tissue were not accounted for here. Further lipidomic profiling should potentially consider reporting separate analyses for white/gray matter. However, the cryomilling approach here produced a homogenous, smooth, fine powder for optimal extraction. Furthermore, the panel of lipids identified here can sensitively and selectively differentiate AD (mild AD and manifest AD) from control cases, including CE, CER, DAG, DCER, LCER, and TAG. These lipid changes are certainly worth investigating in much more accessible biomatrices to determine their utility as a biomarker panel for identifying persons at risk of developing AD.

## 5. Conclusions

The major limitations associated with this study are the sample sizes of each individual group and the lack of clinical information other than that provided herein for each individual specimen. While we do have obvious underpinnings in this work, here the great potential of lipidomics in the field of neurodegeneration has been well illustrated. In this article, we report striking differences in the extent of lipid dysregulation, related to several pathological hallmarks of AD pathology and which become more pronounced with increasing severity of AD. Our preliminary findings have identified some potential biomarkers that might be used to distinguish AD from controls with a high degree of accuracy, but which require replication and further validation. Having a further validated lipid panel for easily accessible non-invasive or nearly non-invasive bio-matrices will be of immense clinical potential for early detection of AD. This study provides significant insights into the importance of lipid metabolism in AD. It indicates that there may be a biochemical cascade directly linked to changes in brain morphology (as gauged by the Braak stage). Finally, this study demonstrated the resolving power of lipidomics in neurodegenerative disease research and the potential for identifying novel biomarkers for the disease. Such an approach may alter future clinical strategies, perhaps leading to the diagnosis of AD with blood serum or saliva samples.

## Figures and Tables

**Table 1:** Multi-group comparison of available demographic information by one-way ANOVA and chi-square tests.

	Controls	Mild AD	AD	*p*-Value
**n**	16	15	15	
**Age, mean (SD)**	79.12 (6.28)	84.573 (8.03)	81.33 (6.51)	0.3306
**Gender**				
**Male**	8	8	7	0.8704
**Female**	8	7	8	
**PMI in hours (SD)**	49.93 (0.45)	38.96 (0.48)	42.07 (0.47)	0.3826

## Data Availability

The lipidomics data represented in this exploratory study will be available upon request.

## Supplementary Materials

The following are available online at https://www.mdpi.com/article/10.3390/cells10102591/s1, Figure S1 Heatmap of thirteen class of lipids selected during Random Forest classification. Table S1: Available demographic information for study subjects. Table S2: Lipid standard mixtures. Table S3: The list of lipids showing statistically significant concentration change (*p* < 0.05; q < 0.05) when Mild-AD sufferers compared to cognitively healthy controls Table S3: The list of lipids showing statistically significant concentration change (*p* < 0.05; q < 0.05) when Mild-AD sufferers compared to AD patients. Table S4: The list of lipids showing statistically significant concentration change (*p* < 0.05; q < 0.05) when Mild-AD sufferers compared to AD patients. Table S5: The list of lipids showing statistically significant concentration change (*p* < 0.05; q < 0.05) when AD patients compared to cognitively healthy controls. Table S6: Optimized model parameters for each machine learning algorithm evaluated for prediction of cognitively healthy controls as compare to Mild-AD. Table S7: Optimized model parameters for each machine learning algorithm evaluated for prediction of Mild-AD as compare to AD. Table S8: Heatmap of thirteen class of lipids selected during Random Forest classification, following recursive feature elimination, which were consistently above the level of quantification (LOQ). AD, Alzheimer’s disease; MAD, Mild-AD (Mild Alzheimer’s disease).

## References

[1] Livingston G., Sommerlad A., Orgeta V., Costafreda S.G., Huntley J., Ames D., Ballard C., Banerjee S., Burns A., Cohen-Mansfield J. (2017). Dementia prevention, intervention, and care. Lancet.

[2] Hebert L.E., Weuve J., Scherr P.A., Evans D.A. (2013). Alzheimer disease in the United States (2010–2050) estimated using the 2010 census. Neurology.

[3] (2020). 2020 Alzheimer’s disease facts and figures. Alzheimer’s Dement..

[4] McKhann G., Drachman D., Folstein M., Katzman R., Price D., Stadlan E.M. (1984). Clinical diagnosis of Alzheimer’s disease: Report of the NINCDS-ADRDA Work Group under the auspices of Department of Health and Human Services Task Force on Alzheimer’s Disease. Neurology.

[5] Karran E., Mercken M., De Strooper B. (2011). The amyloid cascade hypothesis for Alzheimer’s disease: An appraisal for the development of therapeutics. Nat. Rev. Drug Discov..

[6] Savva G.M., Wharton S.B., Ince P.G., Forster G., Matthews F.E., Brayne C. (2009). Age, neuropathology, and dementia. N. Engl. J. Med..

[7] Shaw L.M., Vanderstichele H., Knapik-Czajka M., Clark C.M., Aisen P.S., Petersen R.C., Blennow K., Soares H., Simon A., Lewczuk P. (2009). Cerebrospinal fluid biomarker signature in Alzheimer’s disease neuroimaging initiative subjects. Ann. Neurol..

[8] Pan X., Nasaruddin M.B., Elliott C.T., McGuinness B., Passmore A.P., Kehoe P.G., Hölscher C., McClean P.L., Graham S.F., Green B.D. (2016). Alzheimer’s disease-like pathology has transient effects on the brain and blood metabolome. Neurobiol. Aging.

[9] McKhann G.M., Knopman D.S., Chertkow H., Hyman B.T., Jack C.R., Kawas C.H., Klunk W.E., Koroshetz W.J., Manly J.J., Mayeux R. (2011). The diagnosis of dementia due to Alzheimer’s disease: Recommendations from the National Institute on Aging-Alzheimer’s Association workgroups on diagnostic guidelines for Alzheimer’s disease. Alzheimer’s Dement. J. Alzheimer’s Assoc..

[10] Jack C.R., Bennett D.A., Blennow K., Carrillo M.C., Dunn B., Haeberlein S.B., Holtzman D.M., Jagust W., Jessen F., Karlawish J. (2018). NIA-AA Research Framework: Toward a biological definition of Alzheimer’s disease. Alzheimer’s Dement. J. Alzheimer’s Assoc..

[11] Jack C.R., Wiste H.J., Weigand S.D., Therneau T.M., Lowe V.J., Knopman D.S., Botha H., Graff-Radford J., Jones D.T., Ferman T.J. (2020). Predicting future rates of tau accumulation on PET. Brain.

[12] Wood P.L. (2012). Lipidomics of Alzheimer’s disease: Current status. Alzheimer’s Res. Ther..

[13] Köfeler H.C., Fauland A., Rechberger G.N., Trötzmüller M. (2012). Mass spectrometry based lipidomics: An overview of technological platforms. Metabolites.

[14] Wong M.W., Braidy N., Poljak A., Sachdev P.S. (2017). The application of lipidomics to biomarker research and pathomechanisms in Alzheimer’s disease. Curr. Opin. Psychiatry.

[15] Nasaruddin M.L., Hölscher C., Kehoe P., Graham S.F., Green B.D. (2016). Wide-ranging alterations in the brain fatty acid complement of subjects with late Alzheimer’s disease as detected by GC-MS. Am. J. Transl. Res..

[16] Nasaruddin M.L., Pan X., McGuinness B., Passmore P., Kehoe P.G., Hölscher C., Graham S.F., Green B.D. (2018). Evidence That Parietal Lobe Fatty Acids May Be More Profoundly Affected in Moderate Alzheimer’s Disease (AD) Pathology Than in Severe AD Pathology. Metabolites.

[17] Zhang A.H., Ma Z.M., Kong L., Gao H.L., Sun H., Wang X.Q., Yu J.B., Han Y., Yan G.L., Wang X.J. (2020). High-throughput lipidomics analysis to discover lipid biomarkers and profiles as potential targets for evaluating efficacy of Kai-Xin-San against APP/PS1 transgenic mice based on UPLC-Q/TOF-MS. Biomed. Chromatogr. BMC.

[18] Barupal D.K., Baillie R., Fan S., Saykin A.J., Meikle P.J., Arnold M., Nho K., Fiehn O., Kaddurah-Daouk R., Alzheimer Disease Metabolomics Consortium (2019). Sets of coregulated serum lipids are associated with Alzheimer’s disease pathophysiology. Alzheimer’s Dement..

[19] Wood P.L., Barnette B.L., Kaye J.A., Quinn J.F., Woltjer R.L. (2015). Non-targeted lipidomics of CSF and frontal cortex grey and white matter in control, mild cognitive impairment, and Alzheimer’s disease subjects. Acta Neuropsychiatr..

[20] McKeith I.G., Dickson D.W., Lowe J., Emre M., O’Brien J.T., Feldman H., Cummings J., Duda J.E., Lippa C., Perry E.K. (2005). Diagnosis and management of dementia with Lewy bodies: Third report of the DLB Consortium. Neurology.

[21] Montine T.J., Phelps C.H., Beach T.G., Bigio E.H., Cairns N.J., Dickson D.W., Duyckaerts C., Frosch M.P., Masliah E., Mirra S.S. (2012). National Institute on Aging-Alzheimer’s Association guidelines for the neuropathologic assessment of Alzheimer’s disease: A practical approach. Acta Neuropathol..

[22] Angarita-Zapata J.S., Masegosa A.D., Triguero I. (2020). General-Purpose Automated Machine Learning for Transportation: A Case Study of Auto-Sklearn for Traffic Forecasting.

[23] René de Cotret L.P., Otto M.R., Stern M.J., Siwick B.J. (2018). An open-source software ecosystem for the interactive exploration of ultrafast electron scattering data. Adv. Struct. Chem. Imaging.

[24] Parsons H., Viant M. (2007). Variance stabilising transformations for NMR metabolomics data. BMC Syst. Biol..

[25] Lalwani A.M., Yilmaz A., Bisgin H., Ugur Z., Akyol S., Graham S.F. (2020). The Biochemical Profile of Post-Mortem Brain from People Who Suffered from Epilepsy Reveals Novel Insights into the Etiopathogenesis of the Disease. Metabolites.

[26] Graham S.F., Turkoglu O., Yilmaz A., Ustun I., Ugur Z., Bjorndhal T., Han B., Mandal R., Wishart D., Bahado-Singh R.O. (2020). Targeted metabolomics highlights perturbed metabolism in the brain of autism spectrum disorder sufferers. Metabolomics.

[27] Varma V.R., Oommen A.M., Varma S., Casanova R., An Y., Andrews R.M., O’Brien R., Pletnikova O., Troncoso J.C., Toledo J. (2018). Brain and blood metabolite signatures of pathology and progression in Alzheimer disease: A targeted metabolomics study. PLoS Med..

[28] Tajima Y., Ishikawa M., Maekawa K., Murayama M., Senoo Y., Nishimaki-Mogami T., Nakanishi H., Ikeda K., Arita M., Taguchi R. (2013). Lipidomic analysis of brain tissues and plasma in a mouse model expressing mutated human amyloid precursor protein/tau for Alzheimer’s disease. Lipids Health Dis..

[29] Dart C. (2010). Lipid microdomains and the regulation of ion channel function. J. Physiol..

[30] Schneider A., Schulz-Schaeffer W., Hartmann T., Schulz J.B., Simons M. (2006). Cholesterol depletion reduces aggregation of amyloid-beta peptide in hippocampal neurons. Neurobiol. Dis..

[31] Xiong H., Callaghan D., Jones A., Walker D.G., Lue L.F., Beach T.G., Sue L.I., Woulfe J., Xu H., Stanimirovic D.B. (2008). Cholesterol retention in Alzheimer’s brain is responsible for high beta- and gamma-secretase activities and Abeta production. Neurobiol. Dis..

[32] Grimm M.O., Mett J., Grimm H.S., Hartmann T. (2017). APP Function and Lipids: A Bidirectional Link. Front. Mol. Neurosci..

[33] Corder E.H., Saunders A.M., Strittmatter W.J., Schmechel D.E., Gaskell P.C., Small G.W., Roses A.D., Haines J.L., Pericak-Vance M.A. (1993). Gene dose of apolipoprotein E type 4 allele and the risk of Alzheimer’s disease in late onset families. Science.

[34] Martins I.J., Berger T., Sharman M.J., Verdile G., Fuller S.J., Martins R.N. (2009). Cholesterol metabolism and transport in the pathogenesis of Alzheimer’s disease. J. Neurochem..

[35] Zhao N., Liu C.C., Qiao W., Bu G. (2018). Apolipoprotein E, Receptors, and Modulation of Alzheimer’s Disease. Biol. Psychiatry.

[36] Sato N., Morishita R. (2015). The roles of lipid and glucose metabolism in modulation of β-amyloid, tau, and neurodegeneration in the pathogenesis of Alzheimer disease. Front. Aging Neurosci..

[37] Bandaru V.V.R., Troncoso J., Wheeler D., Pletnikova O., Wang J., Conant K., Haughey N.J. (2009). ApoE4 disrupts sterol and sphingolipid metabolism in Alzheimer’s but not normal brain. Neurobiol. Aging.

[38] El Gaamouch F., Jing P., Xia J., Cai D. (2016). Alzheimer’s Disease Risk Genes and Lipid Regulators. J. Alzheimer’s Dis. JAD.

[39] Frisardi V., Panza F., Seripa D., Farooqui T., Farooqui A.A. (2011). Glycerophospholipids and glycerophospholipid-derived lipid mediators: A complex meshwork in Alzheimer’s disease pathology. Prog. Lipid Res..

[40] Shindou H., Shimizu T. (2009). Acyl-CoA:lysophospholipid acyltransferases. J. Biol. Chem..

[41] Cutler R.G., Kelly J., Storie K., Pedersen W.A., Tammara A., Hatanpaa K., Troncoso J.C., Mattson M.P. (2004). Involvement of oxidative stress-induced abnormalities in ceramide and cholesterol metabolism in brain aging and Alzheimer’s disease. Proc. Natl. Acad. Sci. USA.

[42] González-Domínguez R., García-Barrera T., Gómez-Ariza J.L. (2014). Combination of metabolomic and phospholipid-profiling approaches for the study of Alzheimer’s disease. J. Proteom..

[43] Nitsch R., Pittas A., Blusztajn J.K., Slack B.E., Growdon J.H., Wurtman R.J. (1991). Alterations of phospholipid metabolites in postmortem brain from patients with Alzheimer’s disease. Ann. N. Y. Acad. Sci..

[44] Mulder C., Wahlund L.O., Teerlink T., Blomberg M., Veerhuis R., van Kamp G.J., Scheltens P., Scheffer P.G. (2003). Decreased lysophosphatidylcholine/phosphatidylcholine ratio in cerebrospinal fluid in Alzheimer’s disease. J. Neural. Transm..

[45] Igarashi M., Ma K., Gao F., Kim H.W., Rapoport S.I., Rao J.S. (2011). Disturbed choline plasmalogen and phospholipid fatty acid concentrations in Alzheimer’s disease prefrontal cortex. J. Alzheimer’s Dis. JAD.

[46] Wang W.Y., Tan M.S., Yu J.T., Tan L. (2015). Role of pro-inflammatory cytokines released from microglia in Alzheimer’s disease. Ann. Transl. Med..

[47] Wong M.W., Braidy N., Poljak A., Pickford R., Thambisetty M., Sachdev P.S. (2017). Dysregulation of lipids in Alzheimer’s disease and their role as potential biomarkers. Alzheimer’s Dement. J. Alzheimer’s Assoc..

[48] Mielke M.M., Bandaru V.V.R., Haughey N.J., Xia J., Fried L.P., Yasar S., Albert M., Varma V., Harris G., Schneider E.B. (2012). Serum ceramides increase the risk of Alzheimer disease: The Women’s Health and Aging Study II. Neurology.

[49] Han X., Holtzman D.M., McKeel D.W. (2001). Plasmalogen deficiency in early Alzheimer’s disease subjects and in animal models: Molecular characterization using electrospray ionization mass spectrometry. J. Neurochem..

[50] Posse de Chaves E., Sipione S. (2010). Sphingolipids and gangliosides of the nervous system in membrane function and dysfunction. FEBS Lett..

[51] Tarasiuk J., Kapica-Topczewska K., Kułakowska A., Halicka D., Drozdowski W., Kornhuber J., Lewczuk P. (2012). Increased concentration of the CSF Tau protein and its phosphorylated form in the late juvenile metachromatic leukodystrophy form: A case report. J. Neural. Transm..

[52] Satoi H., Tomimoto H., Ohtani R., Kitano T., Kondo T., Watanabe M., Oka N., Akiguchi I., Furuya S., Hirabayashi Y. (2005). Astroglial expression of ceramide in Alzheimer’s disease brains: A role during neuronal apoptosis. Neuroscience.

[53] Han X.M., Holtzman D.W., McKeel D.W., Kelley J., Morris J.C. (2002). Substantial sulfatide deficiency and ceramide elevation in very early Alzheimer’s disease: Potential role in disease pathogenesis. J. Neurochem..

[54] He X., Huang Y., Li B., Gong C.X., Schuchman E.H. (2010). Deregulation of sphingolipid metabolism in Alzheimer’s disease. Neurobiol. Aging.

[55] Patil S., Melrose J., Chan C. (2007). Involvement of astroglial ceramide in palmitic acid-induced Alzheimer-like changes in primary neurons. Eur. J. Neurosci..

[56] Mielke M.M., Haughey N.J., Bandaru V.V., Weinberg D.D., Darby E., Zaidi N., Pavlik V., Doody R.S., Lyketsos C.G. (2011). Plasma sphingomyelins are associated with cognitive progression in Alzheimer’s disease. J. Alzheimer’s Dis. JAD.

[57] Jazvinšćak Jembrek M., Hof P.R., Šimić G. (2015). Ceramides in Alzheimer’s Disease: Key Mediators of Neuronal Apoptosis Induced by Oxidative Stress and Aβ Accumulation. Oxid. Med. Cell Longev..

[58] Mullen T.D., Obeid L.M. (2012). Ceramide and apoptosis: Exploring the enigmatic connections between sphingolipid metabolism and programmed cell death. Anti-Cancer Agents Med. Chem..

[59] Katsel P., Li C., Haroutunian V. (2007). Gene expression alterations in the sphingolipid metabolism pathways during progression of dementia and Alzheimer’s disease: A shift toward ceramide accumulation at the earliest recognizable stages of Alzheimer’s disease?. Neurochem. Res..

[60] Grimm M.O., Grimm H.S., Pätzold A.J., Zinser E.G., Halonen R., Duering M., Tschäpe J.A., De Strooper B., Müller U., Shen J. (2005). Regulation of cholesterol and sphingomyelin metabolism by amyloid-beta and presenilin. Nat. Cell Biol..

[61] Malaplate-Armand C., Florent-Béchard S., Youssef I., Koziel V., Sponne I., Kriem B., Leininger-Muller B., Olivier J.-L., Oster T., Pillot T. (2006). Soluble oligomers of amyloid-beta peptide induce neuronal apoptosis by activating a cPLA2-dependent sphingomyelinase-ceramide pathway. Neurobiol. Dis..

[62] Kosicek M., Zetterberg H., Andreasen N., Peter-Katalinic J., Hecimovic S. (2012). Elevated cerebrospinal fluid sphingomyelin levels in prodromal Alzheimer’s disease. Neurosci. Lett..

[63] Yanagisawa K. (2015). GM1 ganglioside and Alzheimer’s disease. Glycoconj. J..

[64] Sevigny J., Chiao P., Bussière T., Weinreb P.H., Williams L., Maier M., Dunstan R., Salloway S., Chen T., Ling Y. (2016). The antibody aducanumab reduces Aβ plaques in Alzheimer’s disease. Nature.

[65] Schneider L. (2020). A resurrection of aducanumab for Alzheimer’s disease. Lancet Neurol..

