# Supplementary material for: Lipid Profiling of Alzheimer’s Disease Brain Highlights Enrichment in Glycerol(phospho)lipid, and Sphingolipid Metabolism
Source: Cells. 2021 Sep 29;10(10):2591. doi: 10.3390/cells10102591 (PMC8534054; doi:10.3390/cells10102591)

Metabolic profiling of CSF from people suffering from Sporadic and LRRK2 Parkinson's disease: a pilot study.

Sumeyya Akyol [1, ¥], Zafer Ugur [1, ¥], Ali Yilmaz [1, 2 ¥], Ilyas Ustun [3], Santosh Kapil Kumar Gorti [4], Kyung Joon Oh [1, 5], Bernadette McGuinness [6], Peter Passmore [6], Patrick G. Kehoe [7], Michael E. Maddens [3], Brian D. Green [8] and Stewart F. Graham [1, 3, *]

[1] Metabolomics Department, Beaumont Research Institute, Beaumont Health, Royal Oak, MI, USA
[2] Oakland University-William Beaumont School of Medicine, Rochester, MI, USA
[3] College of Computing and Digital Media, DePaul University, Chicago, IL, 60604
[4] SCIEX
[5] Department of Obstetrics and Gynecology, Seoul National University Bundang Hospital, Seongnam-si, Republic of Korea
[6] Centre for Public Health, School of Medicine, Dentistry and Biomedical Sciences, Queen's University Belfast, Belfast, UK
[7] Dementia Research Group, Translational Health Sciences, Bristol Medical School, University of Bristol, Bristol, UK
[8] Institute for Global Food Security, School of Biological Sciences, Queen's University Belfast, UK

*Correspondence: Stewart F. Graham, Email: stewart.graham@beaumont.org; Phone: +1248-551-2038; Fax: +1248-551-2947
Mailing Address: 3811 W 13 Mile Rd, Suite 415, Royal Oak, MI 48073, USA
¥Authors contributed equally. Academic Editor: name
Received: date; Accepted: date; Published: date

Search Terms: Parkinson Disease, ^{1}H NMR, targeted Mass spectrometry, metabolomics, Machine learning

Page Number	Table of Content
P3-5	**Table S1:** The available demographic information of AD and Mild-AD sufferers and control subjects including Braaking stages
P6	**Table S2:** Lipid standard mixtures.
P7-34	**Table S3:** The list of lipids showing statistically significant concentration change ($p<0.05$; $q<0.05$) when Mild-AD sufferers compared to cognitively healthy controls. Grp0, Control; Grp1, Mild-AD.
P35-55	**Table S4:** The list of lipids showing statistically significant concentration change ($p<0.05$; $q<0.05$) when Mild-AD sufferers compared to AD patients. Grp0, Mild-AD; Grp1, AD.
P56-76	**Table S5:** The list of lipids showing statistically significant concentration change ($p<0.05$; $q<0.05$) when AD patients compared to cognitively healthy controls. Grp0, Control; Grp1, AD.
P77	**Table S6:** Optimized model parameters for each machine learning algorithm evaluated for prediction of cognitively healthy controls as compare to Mild-AD.
P78	**Table S7:** Optimized model parameters for each machine learning algorithm evaluated for prediction of Mild-AD as compare to AD.
P79	**Table S8:** Optimized model parameters for each machine learning algorithm evaluated for prediction of HC as compare to AD.
P80	**Figure S1:** Heatmap of thirteen class of lipids selected during Random Forest classification, following recursive feature elimination, which were consistently above the level of quantification (LOQ). AD, Alzheimer's disease; MAD, Mild-AD (Mild Alzheimer's disease).

Table S1: *The available demographic information of AD and Mild-AD sufferers and control subjects including Braaking stages*

#	Group	Gender	Age	PMI (hours)	Braak area
1	AD	F	71	67	5
2	AD	F	79	27	6
3	AD	F	92	24	5
4	AD	F	75	21	6
5	AD	F	87	36	6
6	AD	F	76	11	5
7	AD	F	73	50	5
8	AD	F	88	28	6
9	AD	M	87	71	5
10	AD	M	75	54	5
11	AD	M	86	72	6
12	AD	M	88	75	5
13	AD	M	83	85	6
14	AD	M	82	110	4
15	AD	M	78	49	6
16	AD	F	77	19	6

17	Mild-AD	F	80	26	4
18	Mild-AD	F	90	13	4
19	Mild-AD	F	89	14	4
20	Mild-AD	F	70	70	3
21	Mild-AD	F	78	21	4
22	Mild-AD	F	72	29	4
23	Mild-AD	M	90	13	3
24	Mild-AD	M	85	58	4
25	Mild-AD	M	95	48	3
26	Mild-AD	M	80	24	4
27	Mild-AD	M	95	27	4
28	Mild-AD	M	81	12	3
29	Mild-AD	M	93	13.5	4
30	Mild-AD	M	81	46.5	4
31	Mild-AD	F	92	19.5	3
32	Control	F	73	59	1
33	Control	F	83	24	2
34	Control	F	82	37	2
35	Control	F	72	24	0

36	Control	F	87	47	2
37	Control	F	88	32	2
38	Control	F	73	50	2
39	Control	F	90	41	2
40	Control	M	80	67	3
41	Control	M	89	91	2
42	Control	M	78	48	1
43	Control	M	76	23	2
44	Control	M	87	24	2
45	Control	M	85	30	2
46	Control	M	77	42	1
47	Control	M	92	34.25	2

Table S2: Lipid standard mixtures.

Lipids Classes Name	Chemical Formula	Concentration (μg/ml)	Exact Mass
15:0-18:1(d7) PC	$C_{41}H_{73}D_7NO_8P$	100	752.6061
18:1(d7) Lyso PC	$C_{26}H_{45}D_7NO_7P$	100	528.3921
15:0-18:1(d7) PE	$C_{38}H_{67}D_7NO_8P$	100	710.5591
18:1(d7) Lyso PE	$C_{23}H_{39}D_7NO_7P$	100	486.3451
15:0-18:1(d7) PG	$C_{39}H_{68}D_7O_{10}P$	100	741.5537
15:0-18:1(d7) PI	$C_{42}H_{72}D_7O_{13}P$	100	829.5698
15:0-18:1(d7) PS	$C_{39}H_{67}D_7NO_{10}P$	100	754.5490
15:0-18:1(d7)-15:0 TAG	$C_{51}H_{89}D_7O_6$	100	811.7646
15:0-18:1(d7) DAG	$C_{36}H_{61}D_7O_5$	100	587.5506
18:1(d7) MAG	$C_{21}H_{33}D_7O_4$	100	363.3366
18:1(d7) Chol Ester	$C_{45}H_{71}D_7O$	100	657.6441
d18:1-18:1(d9) SM	$C_{41}H_{72}D_9N_2O_6P$	100	737.6397
C15 Ceramide-d7	$C_{33}H_{58}D_7NO_3$	100	530.5404

Table S3: The list of lipids showing statistically significant concentration change (p<0.05; q<0.05) when Mild-AD sufferers compared to cognitively healthy controls. Grp0, Control; Grp1, Mild-AD.

Metabolite name	Grp0_Mean	Grp0_Mean-95CI	Grp1_Mean	Grp1_Mean-95CI	P value	Q value	Fold Change
PE(P-18:0/18:1)-H	722930140	(661654720.64, 784205559.33)	310063474	(257939930.78, 362187016.69)	1.1893E-10	3.5796E-08	-2.33155531
PS(18:1/18:2)-H	581464.874	(501259.43, 661670.32)	120039.891	(84567.29, 155512.49)	1.4318E-10	3.5796E-08	-4.84393039
PS(14:0/22:6)-H	143990.725	(98506.72, 189474.73)	751688.158	(650581.51, 852794.81)	1.0032E-09	1.6601E-07	5.2203929
PS(16:0/22:5)-H	4053765.21	(3330389.51, 4777140.92)	455560.987	(249103.57, 662018.4)	1.3898E-09	1.6601E-07	-8.89840291
PS(18:0/16:1)-H	5030752.19	(4331619.19, 5729885.19)	1448291.34	(1299054.13, 1597528.55)	1.6601E-09	1.6601E-07	-3.47357748
PS(18:1/20:5)-H	501220.44	(420034.19, 582406.69)	42429.2965	(28666.74, 56191.85)	2.2252E-09	1.7799E-07	-11.8130745
PS(18:1/20:4)-H	822486.192	(729945.8, 915026.59)	232909.511	(139887.65, 325931.37)	2.4918E-09	1.7799E-07	-3.53135512
PS(18:0/18:3)-H	559793.17	(487870.63, 631715.71)	194764.211	(147103.89, 242424.53)	1.7918E-08	1.1199E-06	-2.87420963
PS(16:0/20:4)-H	2587354.45	(2188602.36, 2986106.54)	161359.011	(88519.97, 234198.06)	3.1514E-08	1.7508E-06	-16.0347688
PS(18:1/20:3)-H	222864.114	(192951.65, 252776.58)	77814.8576	(57035.11, 98594.61)	5.1731E-08	2.5865E-06	-2.86403035
PE(P-18:0/20:1)-H	179140055	(160001512.88, 198278597.44)	81478524.3	(63715787.13, 99241261.49)	9.7807E-08	4.4458E-06	-2.19861683
SM(26:0)+H	10063140.1	(8052971.76, 12073308.36)	2644497.15	(1882090.01, 3406904.29)	1.8392E-07	7.6632E-06	-3.80531325
PS(18:2/18:2)-H	614481.784	(513979.76, 714983.81)	129801.903	(101257.55, 158346.25)	1.383E-06	5.319E-05	-4.73399673
PE(P-18:0/20:3)-H	55618784.2	(51357994.33, 59879574.16)	32462195.7	(25969733.0, 38954658.49)	1.9126E-06	6.8306E-05	-1.71334018

PE(P-18:0/20:2)-H	46316125.5	(42404875.48, 50227375.58)	25224862	(18924944.92, 31524779.04)	3.661E-06	0.00012203	-1.83612999
LCER(d18:0/20:0)+H	45117.7457	(37588.36, 52647.13)	158869.048	(114872.78, 202865.32)	7.4984E-06	0.00023432	3.52120978
PE(O-18:0/20:1)-H	27886674.7	(25169113.6, 30604235.8)	14964407.4	(10905571.77, 19023242.94)	1.6716E-05	0.00049166	-1.86353352
PS(18:2/16:1)-H	2195801.8	(1865408.32, 2526195.27)	1080205.79	(840209.1, 1320202.47)	2.1486E-05	0.00059685	-2.03276248
PE(P-18:0/18:3)-H	582894.376	(413493.9, 752294.85)	119241.055	(69034.07, 169448.05)	2.5364E-05	0.00066747	-4.88836982
HCER(18:1)+H	924946.228	(715296.68, 1134595.77)	228234.637	(130349.62, 326119.65)	2.7494E-05	0.00068734	-4.05261112
PI(20:0/20:4)-H	364913.74	(329015.95, 400811.53)	175516.065	(102793.51, 248238.62)	5.3519E-05	0.00127427	-2.07909026
TAG(53:4/FA20:4)+NH4	10968.8324	(9183.03, 12754.64)	5659.4884	(4559.0, 6759.98)	8.8928E-05	0.0020211	-1.93813143
TAG(57:3/FA18:2)+NH4	11988.8025	(9945.95, 14031.66)	5690.94315	(3980.77, 7401.12)	9.2981E-05	0.00202132	-2.10664598
PE(O-18:0/22:5)-H	13531920.1	(12498263.25, 14565576.95)	9698441.67	(8171143.65, 11225739.69)	0.00027088	0.00564324	-1.39526746
TAG(57:2/FA18:1)+NH4	12089.8942	(9951.49, 14228.3)	6266.58764	(4564.75, 7968.43)	0.00032602	0.00652044	-1.92926277
PE(18:2/20:5)-H	49513.3267	(38641.77, 60384.88)	23703.8036	(17537.85, 29869.75)	0.00042738	0.00821892	-2.08883467
TAG(49:3/FA18:3)+NH4	7107.74958	(5887.05, 8328.45)	3893.66632	(2821.2, 4966.13)	0.0006697	0.01240189	-1.82546448
TAG(58:9/FA22:5)+NH4	11860.3502	(9145.49, 14575.21)	5750.42499	(4395.61, 7105.24)	0.00076384	0.01364002	-2.06251716
TAG(52:1/FA16:1)+NH4	10028.8801	(8458.2, 11599.56)	5819.87147	(4285.56, 7354.19)	0.00080264	0.01383869	-1.72321334
TAG(58:2/FA18:1)+NH4	12801.8436	(10510.54, 15093.14)	7131.03603	(5226.7, 9035.37)	0.00086107	0.01435111	-1.79522913
TAG(46:2/FA18:1)+NH4	41333.5545	(27584.49, 55082.62)	107056.123	(73037.19, 141075.06)	0.0009808	0.01581942	2.59005363

HCER(d18:0/20:0)+H	27432.2665	(19638.08, 35226.45)	12202.4176	(9500.32, 14904.52)	0.00115661	0.01807196	-2.24810095
TAG(60:10/FA22:5)+NH4	14748.0956	(12463.1, 17033.09)	9104.39331	(7105.15, 11103.63)	0.00123369	0.01869232	-1.61988779
TAG(58:3/FA18:1)+NH4	11334.7493	(9278.82, 13390.68)	6195.18011	(4300.16, 8090.2)	0.00132603	0.01950048	-1.82960771
PE(P-18:2/18:2)-H	579188.639	(473431.07, 684946.21)	350988.423	(282091.3, 419885.55)	0.001371	0.01958567	-1.65016451
TAG(53:2/FA18:1)+NH4	14634.7766	(11503.75, 17765.8)	8034.60825	(6332.46, 9736.76)	0.00181153	0.02516018	-1.82146735
TAG(58:8/FA20:3)+NH4	12278.0519	(10514.94, 14041.16)	7591.7812	(5560.54, 9623.02)	0.00187776	0.02519475	-1.61728211
TAG(54:8/FA18:2)+NH4	12199.0984	(9270.5, 15127.69)	6321.26968	(4994.78, 7647.76)	0.00196258	0.02519475	-1.92984938
TAG(52:3/FA18:0)+NH4	11622.5712	(9479.94, 13765.2)	6823.2558	(5139.88, 8506.63)	0.00200947	0.02519475	-1.70337615
LCER(d18:0/24:0)+H	25931.392	(20765.88, 31096.9)	59337.7484	(40815.89, 77859.6)	0.00201558	0.02519475	2.28825928
TAG(54:6/FA20:3)+NH4	10437.1065	(8760.5, 12113.71)	6116.88631	(4233.28, 8000.5)	0.00236167	0.02880088	-1.70627766
TAG(54:7/FA20:4)+NH4	12887.8284	(9980.94, 15794.71)	7095.99522	(5348.47, 8843.52)	0.00316279	0.03765226	-1.81621154
TAG(49:1/FA14:0)+NH4	6781.90004	(5231.54, 8332.26)	3876.04299	(3112.04, 4640.05)	0.00331302	0.03852345	-1.74969681
TAG(56:7/FA18:1)+NH4	16734.3942	(12786.73, 20682.05)	8497.22736	(5651.35, 11343.1)	0.00371437	0.04140838	-1.96939466
TAG(58:7/FA18:1)+NH4	27808.3296	(20785.27, 34831.39)	14609.6718	(11183.8, 18035.54)	0.00372675	0.04140838	-1.90341919
TAG(58:10/FA20:4)+NH4	15413.6733	(11340.46, 19486.89)	7880.89804	(5384.01, 10377.78)	0.0044873	0.04776915	-1.95582702
TAG(58:6/FA16:0)+NH4	12434.4866	(9460.17, 15408.8)	6568.25081	(4488.3, 8648.2)	0.0044903	0.04776915	-1.89311994
TAG(60:10/FA22:6)+NH4	21193.7119	(16397.76, 25989.67)	11227.6651	(7402.01, 15053.32)	0.0047514	0.04949374	-1.88763307

TAG(49:3/FA16:1)+NH4	7772.61814	(5973.41, 9571.82)	4372.18725	(3331.39, 5412.99)	0.00486221	0.04961439	-1.77774137
SM(18:0)+H	2958954.77	(1984077.5, 3933832.04)	1347643.34	(918308.85, 1776977.82)	0.00526959	0.05269587	-2.19565125
TAG(51:4/FA16:1)+NH4	10125.066	(7349.0, 12901.14)	4942.68975	(3773.59, 6111.79)	0.00574137	0.05628794	-2.04849312
CE(22:2)+H	14484.5215	(11071.22, 17897.82)	23508.0019	(18416.91, 28599.09)	0.0060936	0.05859228	1.62297401
TAG(51:2/FA16:1)+NH4	9607.47136	(7508.73, 11706.21)	5752.63052	(4455.05, 7050.21)	0.00664046	0.06264585	-1.67010055
TAG(56:7/FA22:6)+NH4	19653.7612	(15864.12, 23443.41)	10852.13	(6253.01, 15451.25)	0.006937	0.06317413	-1.81105103
TAG(56:4/FA20:3)+NH4	18031.2102	(15162.17, 20900.25)	9881.59279	(5209.8, 14553.38)	0.00694915	0.06317413	-1.8247271
TAG(47:2/FA18:1)+NH4	24496.9807	(19248.7, 29745.26)	41970.2611	(31390.04, 52550.48)	0.00718673	0.0641672	1.71328302
TAG(46:0/FA16:0)+NH4	16769.7147	(12042.27, 21497.16)	7543.3546	(5597.16, 9489.55)	0.00768233	0.06738889	-2.22311102
TAG(54:2/FA18:2)+NH4	10024.9715	(7851.12, 12198.82)	6076.21698	(4556.85, 7595.59)	0.00834168	0.07191106	-1.64987056
TAG(50:3/FA18:3)+NH4	7822.67542	(6786.01, 8859.34)	5540.97981	(4339.87, 6742.09)	0.00876113	0.07397707	-1.41178558
TAG(56:6/FA18:2)+NH4	20999.4044	(16337.36, 25661.45)	12126.7081	(8418.22, 15835.19)	0.00887725	0.07397707	-1.7316657
TAG(45:1/FA18:1)+NH4	9416.99211	(6132.07, 12701.91)	17271.4356	(12864.31, 21678.56)	0.00909163	0.0744971	1.83407137
TAG(55:2/FA18:2)+NH4	10574.8785	(8815.05, 12334.71)	6917.2734	(5086.74, 8747.81)	0.00924045	0.0744971	-1.52876399
TAG(42:1/FA18:1)+NH4	9558.83733	(7061.62, 12056.05)	17392.0918	(11876.18, 22908.01)	0.00938663	0.0744971	1.81947774
TAG(53:2/FA17:0)+NH4	19235.3405	(12754.97, 25715.71)	8466.75833	(5358.45, 11575.06)	0.00962409	0.07518823	-2.27186601
TAG(58:7/FA22:5)+NH4	24582.8245	(18705.19, 30460.46)	14793.4778	(11887.59, 17699.36)	0.01117234	0.08594104	-1.66173396
TAG(51:0/FA17:0)+NH4	9934.22089	(7980.57, 11887.87)	6259.33357	(4542.23, 7976.44)	0.01176193	0.08910552	-1.58710521

TAG(56:7/FA22:5)+NH4	13336.3191	(10585.99, 16086.65)	8715.74739	(7110.76, 10320.74)	0.01249514	0.09056624	-1.53014061
TAG(52:4/FA20:0)+NH4	10852.5615	(8854.99, 12850.13)	6951.11083	(4944.52, 8957.7)	0.01261867	0.09056624	-1.56127011
TAG(58:9/FA20:4)+NH4	16217.1061	(12304.69, 20129.52)	9879.0507	(7689.61, 12068.49)	0.01266283	0.09056624	-1.64156523
TAG(56:4/FA22:4)+NH4	10436.3293	(8268.53, 12604.13)	6549.64413	(4752.23, 8347.06)	0.01280894	0.09056624	-1.59341929
TAG(50:1/FA20:1)+NH4	7059.42311	(5435.14, 8683.71)	4171.49496	(2927.24, 5415.75)	0.01288446	0.09056624	-1.69230052
TAG(52:5/FA22:5)+NH4	8917.40576	(7165.05, 10669.76)	5709.14193	(4156.27, 7262.01)	0.01304154	0.09056624	-1.56195202
TAG(52:5/FA20:5)+NH4	10702.9374	(8611.83, 12794.04)	6978.78684	(5271.37, 8686.21)	0.01428813	0.09786392	-1.53363867
TAG(52:6/FA18:1)+NH4	10892.9417	(8518.04, 13267.84)	7135.35408	(5675.93, 8594.78)	0.01502764	0.10008547	-1.52661544
TAG(56:1/FA16:0)+NH4	12536.5994	(9909.6, 15163.6)	7821.44259	(5539.08, 10103.8)	0.01534373	0.10008547	-1.60285001
TAG(54:5/FA20:5)+NH4	11129.8942	(7840.84, 14418.95)	6055.00518	(4737.97, 7372.04)	0.01545487	0.10008547	-1.83813124
TAG(48:2/FA16:1)+NH4	9745.18598	(7224.6, 12265.78)	5564.96946	(3917.05, 7212.89)	0.01553307	0.10008547	-1.75116612
PE(P-18:2/20:4)-H	3814144.27	(3215487.66, 4412800.87)	2709126.14	(2123814.58, 3294437.7)	0.01574465	0.10008547	-1.40788729
TAG(56:2/FA20:0)+NH4	15449.8556	(10206.92, 20692.79)	8007.67449	(5828.75, 10186.6)	0.0158135	0.10008547	-1.92938107
TAG(56:3/FA16:0)+NH4	14531.9076	(12010.58, 17053.24)	19602.7454	(16589.12, 22616.38)	0.01677195	0.10375385	1.34894509
TAG(52:2/FA20:0)+NH4	12170.6798	(9631.5, 14709.86)	7780.98163	(5725.16, 9836.8)	0.01680812	0.10375385	-1.56415738
TAG(60:11/FA22:6)+NH4	12918.4288	(10112.48, 15724.38)	8335.89434	(6169.5, 10502.28)	0.01716636	0.1046729	-1.54973519
TAG(54:4/FA18:0)+NH4	11613.4045	(9107.55, 14119.26)	7712.07927	(6102.63, 9321.53)	0.01917013	0.11499895	-1.50587203

TAG(48:4/FA14:0)+NH4	7275.5486	(5959.9, 8591.2)	4983.58382	(3765.51, 6201.65)	0.01937902	0.11499895	-1.45990293
TAG(58:6/FA22:5)+NH4	22717.1476	(17674.25, 27760.04)	13975.7358	(9347.68, 18603.79)	0.01954982	0.11499895	-1.62547059
TAG(52:8/FA18:2)+NH4	10525.7204	(7851.35, 13200.09)	6360.59749	(5033.18, 7688.01)	0.02043848	0.1188284	-1.65483202
PS(18:2/20:5)-H	317223.665	(266006.73, 368440.6)	196566.088	(110522.08, 282610.1)	0.02166844	0.12453127	-1.61382702
TAG(46:3/FA14:0)+NH4	9011.85835	(7210.92, 10812.8)	5840.72689	(4014.91, 7666.55)	0.02252844	0.1280025	-1.54293438
TAG(47:1/FA18:1)+NH4	12666.4825	(9282.85, 16050.11)	19025.1764	(14952.23, 23098.13)	0.02494767	0.14015545	1.50200946
TAG(56:9/FA20:5)+NH4	11530.6384	(8305.97, 14755.31)	7050.70674	(5184.29, 8917.12)	0.02566688	0.14112174	-1.63538761
TAG(51:2/FA18:2)+NH4	9796.21001	(7572.8, 12019.62)	6559.24907	(5214.48, 7904.02)	0.02568416	0.14112174	-1.49349566
TAG(56:8/FA22:5)+NH4	10077.6164	(8261.31, 11893.92)	7029.56419	(5306.7, 8752.43)	0.02651377	0.14409659	-1.43360472
HCER(d18:0/26:1)+H	2970.32334	(1677.8, 4262.84)	1355.81381	(930.25, 1781.38)	0.02751547	0.14779111	-2.19080476
TAG(54:5/FA18:2)+NH4	11796.6637	(9985.56, 13607.76)	8435.94618	(6239.9, 10631.99)	0.02778473	0.14779111	-1.39838063
TAG(52:4/FA18:3)+NH4	13101.2969	(10427.76, 15774.83)	8490.69876	(5641.02, 11340.38)	0.02844691	0.14972059	-1.54301752
PE(18:2/22:5)-H	656091.321	(475580.27, 836602.37)	381041.786	(258653.67, 503429.9)	0.02887942	0.15041362	-1.72183563
PS(16:0/20:5)-H	6863045.29	(5696490.18, 8029600.4)	5237820.01	(4683727.42, 5791912.6)	0.03001732	0.15472843	-1.31028658
TAG(50:3/FA20:3)+NH4	10775.7372	(8288.26, 13263.22)	7110.01334	(5336.55, 8883.47)	0.0307064	0.15666532	-1.51557201
CER(14:0)+H	41243.5298	(31458.22, 51028.84)	60214.0923	(46724.29, 73703.89)	0.03111058	0.15712416	1.45996457
TAG(52:5/FA16:1)+NH4	7996.99996	(6005.65, 9988.35)	5273.6472	(3959.28, 6588.02)	0.03165156	0.15825778	-1.51640784

TAG(60:11/FA22:5)+NH4	18218.0222	(13879.56, 22556.49)	11973.9223	(8928.07, 15019.77)	0.03275657	0.16216124	-1.52147489
TAG(58:5/FA18:1)+NH4	12210.7431	(8926.79, 15494.7)	7660.7731	(5653.14, 9668.4)	0.03340867	0.163768	-1.59393092
TAG(52:5/FA20:4)+NH4	9890.7932	(7215.48, 12566.1)	6150.68586	(4416.32, 7885.05)	0.03416186	0.16583427	-1.60807972
TAG(58:6/FA20:4)+NH4	16722.253	(13117.81, 20326.7)	11328.4093	(8451.24, 14205.58)	0.03534535	0.16992955	-1.47613425
TAG(47:0/FA14:0)+NH4	6496.6155	(5100.12, 7893.11)	4406.33063	(3223.11, 5589.55)	0.03596521	0.17126293	-1.47438221
TAG(48:4/FA16:1)+NH4	11727.8455	(9231.3, 14224.39)	7844.63477	(5372.45, 10316.82)	0.03895661	0.1837576	-1.49501485
TAG(52:7/FA18:1)+NH4	12861.9138	(8814.13, 16909.7)	7760.41074	(6016.32, 9504.5)	0.03960261	0.1848271	-1.6573754
TAG(46:3/FA18:3)+NH4	12400.4033	(9066.06, 15734.74)	18023.7032	(14100.5, 21946.9)	0.03992265	0.1848271	1.45347718
TAG(58:8/FA22:5)+NH4	11642.1047	(9206.39, 14077.82)	7899.31771	(5540.27, 10258.36)	0.04129754	0.18687187	-1.47381142
TAG(52:7/FA22:6)+NH4	13594.0281	(11000.25, 16187.8)	9379.57285	(6590.7, 12168.45)	0.04137342	0.18687187	-1.44932272
TAG(46:3/FA18:1)+NH4	11716.7259	(9086.57, 14346.88)	18149.7213	(12593.36, 23706.08)	0.04148831	0.18687187	1.54904377
LPG(22:4)-H	771554.362	(664055.74, 879052.99)	963474.022	(819582.75, 1107365.3)	0.0418593	0.18687187	1.24874418
TAG(56:7/FA20:4)+NH4	12881.9188	(9505.43, 16258.41)	8414.82118	(6296.73, 10532.91)	0.04264125	0.18867808	-1.53086067
TAG(45:0/FA16:0)+NH4	8479.24241	(6830.89, 10127.6)	5648.49636	(3588.71, 7708.28)	0.04317673	0.18937162	-1.50115037
TAG(54:0/FA16:0)+NH4	15600.6764	(10717.03, 20484.32)	9345.55721	(6197.22, 12493.89)	0.04392584	0.19077113	-1.66931474
TAG(53:5/FA20:4)+NH4	11323.0971	(9037.48, 13608.72)	7920.67699	(5782.76, 10058.59)	0.04471061	0.19077113	-1.42956178
TAG(40:0/FA16:0)+NH4	5756.02468	(3948.15, 7563.9)	8650.85776	(6635.13, 10666.59)	0.04601539	0.19077113	1.50292228
TAG(53:3/FA16:0)+NH4	11560.0258	(8448.26, 14671.79)	7749.16584	(6156.15, 9342.18)	0.04609244	0.19077113	-1.4917768

TAG(49:2/FA14:0)+NH4	8112.59227	(6097.53, 10127.65)	5544.43284	(4390.04, 6698.82)	0.04618402	0.19077113	-1.46319606
TAG(50:2/FA18:2)+NH4	10516.9554	(8353.52, 12680.39)	6973.59211	(4397.04, 9550.15)	0.04682949	0.19077113	-1.50811164
TAG(56:6/FA18:3)+NH4	16832.6154	(12656.2, 21009.03)	11739.11	(9868.23, 13609.99)	0.04694554	0.19077113	-1.43389195
TAG(51:3/FA17:0)+NH4	8474.39483	(6199.86, 10748.93)	5607.66839	(4335.48, 6879.86)	0.04750853	0.19077113	-1.5112154
TAG(56:4/FA16:0)+NH4	18801.8073	(12740.14, 24863.48)	10878.9143	(6870.14, 14887.69)	0.04759108	0.19077113	-1.72827976
PE(14:0/16:1)-H	1187820	(1029850.09, 1345789.92)	930272.585	(741643.48, 1118901.7)	0.04805702	0.19077113	-1.27685156
TAG(52:4/FA16:0)+NH4	11776.0107	(9033.31, 14518.71)	8078.51159	(5958.27, 10198.76)	0.04820289	0.19077113	-1.45769559
TAG(53:1/FA17:0)+NH4	14701.5042	(11209.18, 18193.83)	9198.158	(5359.13, 13037.19)	0.04825557	0.19077113	-1.5983096
TAG(52:4/FA18:1)+NH4	11273.0809	(8648.46, 13897.7)	7666.50376	(5686.03, 9646.98)	0.04845587	0.19077113	-1.4704331
TAG(44:2/FA18:1)+NH4	9763.15804	(7510.15, 12016.17)	13512.3753	(10667.29, 16357.47)	0.04987434	0.19482164	1.38401686
TAG(49:2/FA18:2)+NH4	7442.98378	(5139.88, 9746.08)	4443.84049	(3211.79, 5675.89)	0.05112073	0.19783035	-1.67489895
TAG(49:1/FA16:1)+NH4	9148.69846	(6580.89, 11716.51)	6029.4677	(5010.1, 7048.83)	0.05157229	0.19783035	-1.51733103
TAG(55:1/FA16:0)+NH4	10431.1931	(7895.22, 12967.17)	7322.91042	(5807.01, 8838.81)	0.05183155	0.19783035	-1.42446001
TAG(56:9/FA18:3)+NH4	16946.9788	(13187.95, 20706.01)	11773.2916	(8737.35, 14809.23)	0.05224462	0.19789629	-1.43944271
TAG(48:4/FA16:0)+NH4	17230.7129	(13525.51, 20935.92)	23409.019	(18479.22, 28338.82)	0.05543895	0.20841709	1.35856358
TAG(58:6/FA18:1)+NH4	16469.5249	(12010.19, 20928.86)	10731.4659	(7497.18, 13965.76)	0.05641542	0.21050532	-1.5346948
TAG(52:1/FA20:1)+NH4	12109.8819	(10052.13, 14167.63)	8781.00143	(6140.31, 11421.69)	0.05804799	0.21499257	-1.37910032

PS(18:0/20:5)-H	5160404.29	(4386254.49, 5934554.08)	6731898.25	(5308959.13, 8154837.37)	0.05909387	0.21693673	1.30452923
TAG(51:2/FA17:0)+NH4	8960.90893	(6690.55, 11231.26)	6068.06483	(4403.79, 7732.34)	0.05944066	0.21693673	-1.47673256
TAG(56:5/FA22:5)+NH4	11664.1094	(8253.57, 15074.65)	7459.778	(4948.03, 9971.52)	0.05989777	0.21702092	-1.56360006
TAG(52:3/FA20:0)+NH4	10079.6278	(8225.39, 11933.86)	7268.36689	(5108.62, 9428.11)	0.06160502	0.21927343	-1.38678027
HCER(14:0)+H	699199.717	(422662.68, 975736.75)	258565.679	(-15866.1, 532997.45)	0.06166336	0.21927343	-2.70414743
TAG(51:4/FA20:4)+NH4	8337.41934	(6286.55, 10388.29)	5745.84182	(4343.55, 7148.14)	0.06183511	0.21927343	-1.45103531
LPG(20:2)-H	1784974.21	(1512224.69, 2057723.73)	2183955.72	(1886222.52, 2481688.92)	0.0637414	0.22256958	1.22352228
TAG(42:2/FA18:2)+NH4	5233.42293	(3551.98, 6914.86)	8424.00504	(5666.38, 11181.63)	0.06378665	0.22256958	1.60965493
TAG(50:4/FA20:3)+NH4	21368.1357	(16104.97, 26631.3)	14716.1162	(10713.32, 18718.92)	0.06410004	0.22256958	-1.45202276
TAG(56:3/FA18:2)+NH4	12700.7043	(9050.68, 16350.73)	8336.84245	(5783.91, 10889.78)	0.06506733	0.22437009	-1.52344301
TAG(50:2/FA16:0)+NH4	15975.7255	(11788.46, 20162.99)	10532.4627	(6547.92, 14517.01)	0.0755239	0.25864349	-1.51680818
TAG(53:3/FA17:0)+NH4	11716.7467	(6792.42, 16641.07)	6292.54949	(4399.58, 8185.52)	0.07770059	0.26428773	-1.8620031
TAG(52:6/FA16:0)+NH4	8972.0916	(6634.46, 11309.72)	6287.59851	(4914.61, 7660.58)	0.07872165	0.26588805	-1.42695046
TAG(52:8/FA16:1)+NH4	8065.05406	(6027.21, 10102.9)	5318.33261	(3183.27, 7453.39)	0.07923464	0.26588805	-1.51646289
TAG(52:3/FA18:1)+NH4	19109.6057	(12531.93, 25687.28)	12036.1817	(8785.48, 15286.89)	0.08152428	0.27174761	-1.58768006
TAG(54:3/FA16:1)+NH4	12148.3519	(10043.33, 14253.37)	9334.20525	(7106.92, 11561.49)	0.08288377	0.27444956	-1.30148755
CE(18:3)+H	34485.5443	(26990.03, 41981.06)	48518.7439	(34496.88, 62540.61)	0.08398898	0.27627952	1.40692992
TAG(54:4/FA20:3)+NH4	10128.87	(7920.82, 12336.92)	13077.1825	(10672.74, 15481.62)	0.08859243	0.2891973	1.29108011

TAG(56:1/FA18:1)+NH4	12805.249	(9897.84, 15712.66)	8584.16427	(4816.74, 12351.59)	0.08907277	0.2891973	-1.49172926
TAG(54:4/FA18:3)+NH4	13005.1114	(9883.98, 16126.25)	18729.9665	(12901.75, 24558.18)	0.08973192	0.28945781	1.44020039
TAG(56:6/FA22:5)+NH4	19390.8217	(14140.57, 24641.07)	13059.2715	(8460.07, 17658.47)	0.0903709	0.28965032	-1.4848318
TAG(50:1/FA18:1)+NH4	24998.159	(14989.89, 35006.43)	12315.9593	(6433.65, 18198.27)	0.09231066	0.29362611	-2.02973705
TAG(56:3/FA20:1)+NH4	19435.3335	(14717.5, 24153.16)	13652.9963	(9252.36, 18053.64)	0.09278585	0.29362611	-1.42352148
TAG(56:5/FA20:3)+NH4	18751.6244	(14207.67, 23295.57)	13514.7503	(9831.37, 17198.13)	0.0960281	0.30197516	-1.38749322
TAG(44:1/FA14:0)+NH4	5264.19825	(4116.71, 6411.69)	3653.52511	(2184.49, 5122.56)	0.09694946	0.30296705	-1.44085454
TAG(58:7/FA18:2)+NH4	16859.8087	(13089.77, 20629.85)	12720.6685	(10027.1, 15414.24)	0.09920882	0.30695186	-1.325387
TAG(58:9/FA18:2)+NH4	14790.346	(10022.99, 19557.7)	9881.35467	(7063.76, 12698.95)	0.0994524	0.30695186	-1.49679336
TAG(50:1/FA16:1)+NH4	10587.908	(7466.23, 13709.59)	7252.86171	(5215.79, 9289.93)	0.10037109	0.30730363	-1.45982488
DAG(18:0/18:3)+NH4	159461.47	(109168.12, 209754.82)	107800.366	(80086.5, 135514.23)	0.10133795	0.30730363	-1.4792294
TAG(46:1/FA14:0)+NH4	8925.47076	(6386.72, 11464.22)	6224.32255	(4573.83, 7874.81)	0.1014102	0.30730363	-1.43396662
DCER(18:1)+H	23538.6348	(19261.49, 27815.78)	31763.5472	(22749.81, 40777.28)	0.10345775	0.30874416	1.34942181
DAG(16:0/16:0)+NH4	70756.6561	(48548.18, 92965.13)	146922.878	(48397.62, 245448.13)	0.10399022	0.30874416	2.0764531
TAG(54:5/FA20:2)+NH4	12131.5614	(9997.65, 14265.47)	9174.40139	(6434.67, 11914.13)	0.10415248	0.30874416	-1.32232729
PE(O-18:0/20:2)-H	3638017	(2340902.5, 4935131.49)	5140462.47	(3992318.74, 6288606.2)	0.10443312	0.30874416	1.41298473
TAG(56:3/FA20:0)+NH4	12965.8724	(10711.27, 15220.47)	16216.7234	(13040.58, 19392.87)	0.10497301	0.30874416	1.25072366
TAG(51:4/FA18:3)+NH4	8567.88931	(6996.69, 10139.09)	6518.58457	(4674.53, 8362.64)	0.10853148	0.3173435	-1.31437879

TAG(56:2/FA20:1)+NH4	12216.4723	(10221.77, 14211.18)	9382.32826	(6578.12, 12186.54)	0.10950203	0.31751956	-1.30207257
TAG(56:2/FA16:0)+NH4	11553.4315	(9004.1, 14102.76)	8697.07535	(6681.97, 10712.18)	0.10986177	0.31751956	-1.32842721
CE(20:4)+H	32978.6172	(24802.86, 41154.37)	45307.5727	(32282.71, 58332.44)	0.11449275	0.32900217	1.37384695
TAG(51:0/FA18:0)+NH4	10097.6425	(6901.95, 13293.33)	6809.68822	(5095.2, 8524.18)	0.11839417	0.33559027	-1.48283478
TAG(50:5/FA14:0)+NH4	9411.38048	(6974.75, 11848.02)	6802.07974	(4960.64, 8643.52)	0.11901491	0.33559027	-1.38360337
TAG(54:4/FA20:2)+NH4	10204.207	(7729.51, 12678.9)	7519.79624	(5559.64, 9479.96)	0.119114	0.33559027	-1.35697919
TAG(57:10/FA22:6)+NH4	15311.9252	(11769.05, 18854.8)	11187.641	(7909.9, 14465.38)	0.11947014	0.33559027	-1.36864646
TAG(50:3/FA18:2)+NH4	7240.24896	(5933.8, 8546.7)	5616.21739	(4111.79, 7120.64)	0.12040079	0.33631506	-1.28916822
TAG(58:8/FA18:2)+NH4	38131.8532	(25928.79, 50334.92)	26076.8164	(18531.43, 33622.2)	0.12280511	0.34112531	-1.46228944
TAG(54:7/FA18:1)+NH4	10844.2304	(7232.35, 14456.11)	7197.28829	(4950.85, 9443.72)	0.12367736	0.34165016	-1.50671058
LPG(22:6)-H	70357.8061	(57919.89, 82795.72)	55577.5682	(42061.69, 69093.45)	0.12558044	0.34427291	-1.26593891
TAG(50:3/FA14:0)+NH4	7186.84488	(5247.09, 9126.6)	5015.13251	(3433.09, 6597.18)	0.12600388	0.34427291	-1.4330319
CE(18:2)+H	27986.4426	(20186.74, 35786.14)	38747.0712	(27475.37, 50018.78)	0.1276242	0.3468049	1.38449434
TAG(53:1/FA16:0)+NH4	9759.22153	(7545.43, 11973.02)	7415.5691	(5594.12, 9237.02)	0.12983129	0.35089539	-1.31604485
TAG(56:7/FA16:0)+NH4	12657.873	(9636.85, 15678.89)	9474.30113	(7021.7, 11926.91)	0.13221447	0.35541523	-1.33602181
TAG(46:3/FA16:1)+NH4	11307.2763	(8142.84, 14471.72)	8212.63719	(5851.87, 10573.4)	0.14037011	0.37167866	-1.3768143
DAG(18:2/18:3)+NH4	27520.5275	(20013.5, 35027.55)	20066.2063	(14373.78, 25758.63)	0.14077131	0.37167866	-1.37148632

TAG(54:0/FA18:0) +NH4	15160.7671	(10905.96, 19415.57)	11196.7873	(9025.72, 13367.85)	0.1409007	0.37167866	-1.35402832
TAG(47:1/FA16:1) +NH4	9215.35456	(6886.7, 11544.01)	7020.97079	(5564.97, 8476.97)	0.14123789	0.37167866	-1.31254706
PS(16:0/22:6)-H	885506.579	(622185.85, 1148827.3)	656210.676	(515116.05, 797305.3)	0.14367959	0.37612459	-1.34942422
PE(P-18:1/18:3)-H	872029.024	(410950.8, 1333107.25)	1360655.19	(937313.24, 1783997.15)	0.14531038	0.37841245	1.56033246
TAG(54:1/FA16:0) +NH4	14385.9867	(10653.88, 18118.09)	10530.9799	(7195.34, 13866.62)	0.14710261	0.38109485	-1.36606345
TAG(56:6/FA22:4) +NH4	11456.3656	(9014.66, 13898.07)	8850.48923	(6486.31, 11214.66)	0.15014503	0.38697172	-1.29443303
TAG(54:5/FA18:1) +NH4	13749.3317	(11396.63, 16102.03)	10752.0534	(7442.16, 14061.95)	0.15172127	0.3890289	-1.27876334
TAG(56:4/FA18:0) +NH4	16787.2372	(13327.1, 20247.38)	12726.2876	(8447.03, 17005.55)	0.15494549	0.39526911	-1.31909931
TAG(56:7/FA22:4) +NH4	10683.7215	(8517.6, 12849.85)	8497.40941	(6570.94, 10423.88)	0.16020143	0.40660262	-1.25729161
TAG(56:7/FA16:1) +NH4	36014.8182	(27143.38, 44886.25)	26783.9901	(17802.33, 35765.65)	0.16441557	0.41519082	-1.34463977
TAG(50:2/FA16:1) +NH4	8723.56226	(6630.41, 10816.71)	6275.47733	(3561.96, 8989.0)	0.16677604	0.41593346	-1.39010338
TAG(54:6/FA16:0) +NH4	16570.4568	(11247.51, 21893.4)	11350.3049	(8348.12, 14352.49)	0.16696278	0.41593346	-1.45991292
TAG(58:9/FA18:1) +NH4	16173.4001	(11707.32, 20639.48)	11111.4169	(5717.12, 16505.72)	0.16720525	0.41593346	-1.45556595
TAG(51:5/FA18:3) +NH4	9919.93924	(8107.91, 11731.96)	7920.24784	(5794.22, 10046.27)	0.1689443	0.41747308	-1.25247839
TAG(49:0/FA18:0) +NH4	9465.85274	(6718.64, 12213.07)	7081.11438	(5666.96, 8495.27)	0.16949407	0.41747308	-1.33677444
TAG(46:0/FA18:0) +NH4	10571.064	(7209.48, 13932.65)	7513.33278	(5046.34, 9980.32)	0.17187202	0.42125495	-1.40697402
TAG(42:0/FA16:0) +NH4	10107.1662	(7838.73, 12375.6)	7475.41976	(4414.27, 10536.57)	0.17822099	0.43411654	-1.35205333

TAG(52:5/FA18:2)+NH4	9859.55532	(7665.2, 12053.91)	7276.14111	(4245.94, 10306.34)	0.17941127	0.43411654	-1.35505279
TAG(56:10/FA18:2)+NH4	12376.0735	(7652.08, 17100.07)	8386.29053	(5936.32, 10836.26)	0.18139227	0.43411654	-1.47575062
HCER(d18:0/18:0)+H	2851.09315	(1441.74, 4260.45)	1798.98704	(1399.8, 2198.18)	0.18182345	0.43411654	-1.58483251
TAG(58:7/FA20:4)+NH4	18089.6507	(14484.49, 21694.81)	14795.0195	(11894.04, 17696.0)	0.18185321	0.43411654	-1.22268515
PS(18:2/20:1)-H	285263.8	(237571.97, 332955.63)	242792.361	(204157.03, 281427.69)	0.18232895	0.43411654	-1.17492906
TAG(54:6/FA16:1)+NH4	13543.1055	(10576.32, 16509.89)	10494.1909	(7325.95, 13662.43)	0.18520905	0.438884	-1.29053356
TAG(55:5/FA18:1)+NH4	18082.659	(14243.17, 21922.15)	14406.0263	(10789.23, 18022.82)	0.18645259	0.43974667	-1.25521491
TAG(48:5/FA18:2)+NH4	9321.35187	(7110.17, 11532.53)	12168.9687	(8479.33, 15858.6)	0.18738878	0.43987977	1.30549398
TAG(48:1/FA18:0)+NH4	10611.8621	(7186.71, 14037.01)	7600.06828	(5472.8, 9727.33)	0.18890641	0.44137012	-1.39628509
TAG(56:8/FA18:1)+NH4	11915.3792	(9587.4, 14243.36)	9613.62404	(7217.91, 12009.34)	0.19150828	0.44441376	-1.23942637
TAG(56:8/FA16:1)+NH4	15301.8326	(12149.55, 18454.11)	11310.1735	(6189.85, 16430.5)	0.19198674	0.44441376	-1.35292642
TAG(52:3/FA16:0)+NH4	15213.6444	(12142.23, 18285.06)	11525.2017	(6672.68, 16377.72)	0.19728685	0.454578	-1.32003281
TAG(54:5/FA22:5)+NH4	10126.308	(7663.09, 12589.52)	7957.26742	(5958.76, 9955.77)	0.19897339	0.45625095	-1.27258611
TAG(56:7/FA18:3)+NH4	23513.0039	(17320.38, 29705.63)	18264.4542	(13619.45, 22909.46)	0.19983792	0.45625095	-1.28736417
TAG(46:2/FA16:0)+NH4	7675.04756	(6034.26, 9315.84)	9619.31784	(7090.82, 12147.81)	0.20186265	0.45877874	1.25332355
TAG(50:3/FA18:0)+NH4	10760.9153	(8308.46, 13213.37)	8528.78487	(6389.69, 10667.88)	0.2080683	0.46645938	-1.26171729
TAG(52:3/FA14:0)+NH4	8084.49038	(6096.59, 10072.39)	6301.55706	(4552.0, 8051.11)	0.2087355	0.46645938	-1.28293536

PS(14:0/20:3)-H	154543.107	(115601.07, 193485.14)	123047.967	(95005.55, 151090.39)	0.2088492	0.46645938	-1.25595823
TAG(52:5/FA16:0)+NH4	8525.55643	(6796.83, 10254.28)	6758.69121	(4676.93, 8840.46)	0.20916686	0.46645938	-1.26142121
TAG(50:1/FA14:0)+NH4	8168.92737	(5902.57, 10435.28)	6240.42445	(4552.59, 7928.26)	0.20990672	0.46645938	-1.30903393
TAG(52:6/FA16:1)+NH4	7549.87534	(5420.95, 9678.8)	5765.29035	(4049.08, 7481.5)	0.21215543	0.46651377	-1.30953948
PS(18:1/20:1)-H	161288.208	(136755.54, 185820.87)	140948.065	(121100.54, 160795.59)	0.21296803	0.46651377	-1.14430949
TAG(56:8/FA18:3)+NH4	14892.3919	(10810.94, 18973.84)	11637.9149	(9019.52, 14256.31)	0.21333615	0.46651377	-1.27964434
TAG(52:2/FA16:1)+NH4	11546.5074	(8302.04, 14790.97)	8659.83176	(5773.14, 11546.53)	0.21392195	0.46651377	-1.33334085
TAG(54:7/FA16:1)+NH4	15930.5771	(7068.87, 24792.29)	9477.77255	(6253.18, 12702.37)	0.21459633	0.46651377	-1.68083556
TAG(54:3/FA16:0)+NH4	13795.994	(10560.19, 17031.79)	10968.8889	(7990.45, 13947.33)	0.22269313	0.48201977	-1.2577385
TAG(52:5/FA18:3)+NH4	8403.53369	(6781.73, 10025.34)	6697.21597	(4538.85, 8855.58)	0.2257182	0.48471181	-1.25478015
TAG(52:2/FA20:1)+NH4	10152.8149	(7640.04, 12665.59)	7992.02429	(5727.35, 10256.7)	0.22619065	0.48471181	-1.27036837
TAG(54:5/FA22:4)+NH4	11407.7751	(8501.32, 14314.23)	14666.1602	(10230.99, 19101.33)	0.22777076	0.48471181	1.28562844
TAG(54:3/FA18:3)+NH4	10127.9324	(7724.34, 12531.52)	7906.61248	(5317.52, 10495.71)	0.22781455	0.48471181	-1.28094458
DAG(14:0/14:0)+NH4	283272.121	(163123.55, 403420.69)	197071.739	(142817.1, 251326.38)	0.23211518	0.49176944	-1.4374061
TAG(56:9/FA20:4)+NH4	8959.34871	(6311.53, 11607.16)	6743.56865	(4346.98, 9140.16)	0.23412063	0.49392538	-1.32857678
TAG(50:5/FA18:3)+NH4	23434.9629	(15278.39, 31591.54)	17109.5123	(11889.39, 22329.64)	0.23549819	0.49474409	-1.36970373
CE(20:3)+H	14047.8996	(9656.22, 18439.58)	17990.7033	(13260.37, 22721.04)	0.24100721	0.50090214	1.28066855

TAG(54:2/FA18:1)+NH4	27710.984	(15218.56, 40203.41)	17357.7415	(7195.36, 27520.12)	0.24142899	0.50090214	-1.59646253
TAG(58:10/FA18:2)+NH4	13746.3828	(7784.42, 19708.34)	9233.30177	(5624.25, 12842.35)	0.24143483	0.50090214	-1.48878301
PE(18:2/20:4)-H	3579906.9	(3103048.58, 4056765.22)	2885047.78	(1653719.05, 4116376.52)	0.24279971	0.5016523	-1.24084839
TAG(58:8/FA20:4)+NH4	24621.8347	(18864.75, 30378.92)	19752.4513	(14365.15, 25139.75)	0.24487896	0.50386617	-1.24652046
TAG(54:4/FA22:4)+NH4	9588.47397	(7276.39, 11900.56)	7723.75697	(5680.89, 9766.62)	0.24613279	0.50437047	-1.24142616
TAG(54:4/FA20:4)+NH4	11330.6141	(8487.92, 14173.31)	8893.40354	(6022.18, 11764.63)	0.24884088	0.50783853	-1.274047
TAG(54:4/FA16:1)+NH4	13521.1388	(11707.09, 15335.19)	11051.6766	(6914.02, 15189.33)	0.25250192	0.51321527	-1.22344684
TAG(54:6/FA20:5)+NH4	15264.7395	(11057.07, 19472.41)	11888.3656	(8179.53, 15597.2)	0.25407433	0.51432051	-1.28400657
TAG(54:5/FA16:1)+NH4	20598.6615	(13364.1, 27833.22)	15413.8998	(10904.06, 19923.74)	0.25898222	0.52021445	-1.33636923
TAG(49:2/FA16:1)+NH4	8022.10351	(5838.8, 10205.41)	6237.62103	(4214.75, 8260.49)	0.2590668	0.52021445	-1.28608382
TAG(56:4/FA18:1)+NH4	11751.8583	(8902.15, 14601.57)	14504.2419	(10646.5, 18361.98)	0.26072561	0.52145123	1.23420837
TAG(52:6/FA18:3)+NH4	9696.39505	(8326.45, 11066.34)	8334.95082	(6353.63, 10316.27)	0.2646904	0.52727171	-1.1633416
TAG(54:7/FA18:3)+NH4	11399.9249	(7202.41, 15597.44)	8508.27414	(6186.35, 10830.19)	0.26585076	0.52748167	-1.33986338
DAG(16:0/20:5)+NH4	72603.1409	(52365.13, 92841.15)	57727.773	(41894.96, 73560.59)	0.2759222	0.54530039	-1.2576813
TAG(51:5/FA18:2)+NH4	8400.71212	(6662.26, 10139.16)	7117.53734	(5732.13, 8502.94)	0.28391137	0.55888064	-1.18028353
TAG(49:2/FA16:0)+NH4	8408.03992	(5430.42, 11385.66)	10607.0226	(8182.34, 13031.71)	0.28668137	0.56000949	1.26153333
TAG(49:2/FA17:0)+NH4	8957.56258	(6123.33, 11791.8)	6567.50364	(3279.05, 9855.96)	0.2878376	0.56000949	-1.36392198

TAG(56:6/FA22:6)+NH4	17495.5404	(9151.61, 25839.47)	12005.2813	(9504.85, 14505.71)	0.28784488	0.56000949	-1.45732032
TAG(52:6/FA20:4)+NH4	8709.40341	(6983.67, 10435.13)	7326.22972	(5503.8, 9148.66)	0.29059064	0.56253758	-1.18879748
PE(O-18:0/18:0)-H	1493405.39	(1157916.74, 1828894.05)	1821315.51	(1308285.34, 2334345.68)	0.292139	0.56253758	1.21957207
TAG(51:2/FA16:0)+NH4	11244.2026	(7331.57, 15156.84)	8658.56573	(6153.56, 11163.57)	0.29251954	0.56253758	-1.29862185
TAG(52:7/FA16:0)+NH4	12595.738	(9589.9, 15601.58)	10238.8105	(7190.91, 13286.71)	0.29461223	0.56439124	-1.23019545
TAG(54:5/FA20:3)+NH4	9426.65048	(6619.56, 12233.74)	7633.76384	(6121.41, 9146.12)	0.29920456	0.57100107	-1.23486273
TAG(53:0/FA16:0)+NH4	10193.1933	(8386.09, 12000.29)	8623.41412	(6279.71, 10967.12)	0.30153436	0.57174457	-1.18203686
TAG(51:1/FA17:0)+NH4	11211.0263	(8901.81, 13520.24)	9292.84768	(6534.42, 12051.27)	0.30188113	0.57174457	-1.20641451
TAG(47:2/FA18:2)+NH4	9801.78019	(7888.59, 11714.97)	11872.7932	(8176.57, 15569.02)	0.30491879	0.57523567	1.21128947
LCER(22:1)+H	20009.8718	(13887.41, 26132.34)	24958.8033	(17888.62, 32028.98)	0.30602538	0.57523567	1.2473245
DAG(16:1/20:2)+NH4	132841.833	(108402.77, 157280.89)	114568.692	(91076.87, 138060.51)	0.30732313	0.57551148	-1.15949506
TAG(45:0/FA14:0)+NH4	6333.37495	(3801.29, 8865.46)	4674.3344	(3147.46, 6201.21)	0.30872299	0.57597572	-1.35492552
LCER(26:1)+H	10765.2169	(7292.86, 14237.58)	8498.7705	(6171.83, 10825.71)	0.31124107	0.57851501	-1.26667932
TAG(48:3/FA18:1)+NH4	15864.9306	(11909.1, 19820.77)	12829.4815	(8587.19, 17071.77)	0.31371651	0.5809565	-1.23659952
CE(22:5)+H	17780.0462	(13315.98, 22244.12)	21782.4913	(15339.26, 28225.72)	0.31592169	0.58114236	1.22510881
PS(18:1/20:2)-H	61788.0105	(37695.52, 85880.5)	74384.7075	(65664.65, 83104.77)	0.31685144	0.58114236	1.2038696
TAG(58:7/FA22:4)+NH4	12891.375	(9727.13, 16055.62)	10357.8086	(6646.57, 14069.05)	0.31730373	0.58114236	-1.24460448
TAG(48:3/FA18:2)+NH4	9993.72588	(6781.07, 13206.38)	13055.5236	(7895.92, 18215.12)	0.31924685	0.58256725	1.306372

PS(14:0/22:4)-H	1224637.17	(1035181.15, 1414093.18)	1021840.99	(669065.42, 1374616.57)	0.32068811	0.58306929	-1.19846158
TAG(50:3/FA16:1)+NH4	8208.67708	(6513.15, 9904.21)	6921.87744	(5069.5, 8774.26)	0.3230985	0.58532337	-1.18590327
TAG(51:1/FA16:0)+NH4	10637.243	(8690.39, 12584.1)	8598.15901	(4875.74, 12320.58)	0.32449545	0.58573185	-1.23715355
TAG(47:1/FA17:0)+NH4	10439.2569	(8330.47, 12548.05)	12017.1994	(9804.41, 14229.99)	0.32767478	0.58664396	1.15115468
TAG(50:0/FA16:0)+NH4	18478.4938	(9662.24, 27294.75)	12557.6241	(6041.31, 19073.94)	0.32897552	0.58664396	-1.47149602
TAG(54:2/FA18:0)+NH4	19753.8174	(10914.49, 28593.14)	14507.6713	(9982.62, 19032.72)	0.32986695	0.58664396	-1.36161186
LCER(24:1)+H	41971.6687	(30026.46, 53916.87)	33610.5045	(25204.43, 42016.57)	0.32991901	0.58664396	-1.2487664
TAG(52:1/FA20:0)+NH4	10866.9548	(8727.8, 13006.11)	9022.2688	(6015.16, 12029.37)	0.33086719	0.58664396	-1.20445921
TAG(51:0/FA16:0)+NH4	10146.2522	(7844.04, 12448.46)	8373.23733	(5665.44, 11081.03)	0.33472072	0.58970096	-1.21174784
TAG(48:4/FA18:2)+NH4	10552.9434	(9195.4, 11910.48)	12904.1996	(7952.47, 17855.93)	0.33495014	0.58970096	1.22280572
TAG(48:3/FA14:0)+NH4	8844.15576	(6088.71, 11599.61)	7058.77287	(4879.33, 9238.21)	0.33734453	0.59183251	-1.25293106
LPG(20:1)-H	3148490.24	(2648646.94, 3648333.54)	3569732.66	(2861284.44, 4278180.89)	0.3401023	0.59458445	1.13379188
TAG(49:1/FA16:0)+NH4	9139.39469	(6417.62, 11861.17)	7423.03805	(5515.16, 9330.91)	0.34233565	0.5961667	-1.23122024
PG(18:1/22:5)-H	1074514.98	(959104.39, 1189925.58)	1477681.71	(665966.96, 2289396.46)	0.34339202	0.5961667	1.3752081
TAG(46:1/FA18:0)+NH4	11286.9278	(7016.5, 15557.35)	14086.9695	(10308.19, 17865.74)	0.34700705	0.59980019	1.24807829
PE(14:0/14:0)-H	312843.065	(270371.16, 355314.97)	344481.662	(294930.86, 394032.46)	0.34788411	0.59980019	1.10113249
TAG(48:2/FA18:2)+NH4	9954.24641	(7637.36, 12271.14)	12474.2559	(7453.98, 17494.53)	0.34986938	0.60115015	1.25315925
TAG(53:4/FA18:2)+NH4	8804.13773	(7000.8, 10607.47)	7512.25341	(5532.96, 9491.55)	0.35172065	0.60215961	-1.17197028
TAG(48:5/FA18:3)+NH4	8798.02196	(7013.09, 10582.95)	10460.6211	(7314.7, 13606.54)	0.35363056	0.60215961	1.18897419

DAG(16:0/18:1)+NH4	299053.793	(211644.08, 386463.5)	598900.199	(-61934.56, 1259734.96)	0.35406985	0.60215961	2.0026504
CE(16:0)+H	23136.0305	(16334.68, 29937.38)	28343.5402	(19685.25, 37001.83)	0.35830795	0.60619458	1.22508225
TAG(54:7/FA20:5)+NH4	10722.7721	(7432.14, 14013.4)	8652.26883	(5930.46, 11374.07)	0.35967297	0.60619458	-1.23930177
PG(14:1/14:1)-H	10986.7238	(6403.33, 15570.12)	14102.3969	(9421.17, 18783.62)	0.36073352	0.60619458	1.28358528
TAG(45:1/FA16:0)+NH4	6675.03178	(4923.63, 8426.43)	7837.67169	(6242.47, 9432.88)	0.36129197	0.60619458	1.17417743
TAG(46:4/FA18:2)+NH4	11140.1085	(7466.49, 14813.72)	15238.8276	(6085.88, 24391.78)	0.36377779	0.60832407	1.36792452
TAG(58:7/FA18:0)+NH4	15289.0767	(11672.05, 18906.11)	12692.9003	(8456.08, 16929.72)	0.3672081	0.6120135	-1.20453768
TAG(55:4/FA18:1)+NH4	11980.233	(9676.72, 14283.74)	13778.0119	(10544.65, 17011.37)	0.37369582	0.62075718	1.1500621
TAG(54:8/FA20:4)+NH4	9554.78192	(7156.26, 11953.31)	8093.48469	(6177.32, 10009.65)	0.37514414	0.62109957	-1.18055229
TAG(50:2/FA20:2)+NH4	9026.0373	(7018.53, 11033.54)	7660.03391	(5459.78, 9860.29)	0.37725992	0.62254112	-1.17832864
TAG(56:8/FA20:5)+NH4	10857.1926	(8179.49, 13534.9)	9090.24746	(6289.14, 11891.36)	0.37979856	0.62466868	-1.19437811
TAG(55:5/FA18:2)+NH4	11516.9152	(9279.63, 13754.2)	9952.94538	(7258.43, 12647.46)	0.38503656	0.63120747	-1.15713638
TAG(48:3/FA16:1)+NH4	9011.2463	(5813.83, 12208.66)	7172.84029	(4682.36, 9663.32)	0.38747812	0.6318937	-1.25630098
TAG(50:5/FA20:5)+NH4	16896.3483	(11979.14, 21813.56)	13864.0788	(9290.49, 18437.67)	0.38815935	0.6318937	-1.2187141
TAG(42:1/FA16:0)+NH4	7945.55334	(5351.58, 10539.53)	9557.95363	(7034.19, 12081.71)	0.39053529	0.6318937	1.20293115
TAG(50:4/FA16:0)+NH4	15433.4086	(11390.34, 19476.48)	12860.119	(8729.07, 16991.16)	0.3908154	0.6318937	-1.20009842
TAG(52:2/FA18:0)+NH4	11240.1319	(6726.37, 15753.9)	8660.29265	(5203.51, 12117.08)	0.3917741	0.6318937	-1.29789285
PE(P-18:0/22:6)-H	332576735	(297427220.9, 367726248.53)	313320586	(291480509.75, 335160661.32)	0.39607705	0.63238308	-1.0614583
TAG(54:1/FA18:0)+NH4	30564.9106	(1358.72, 59771.1)	16744.467	(7853.98, 25634.95)	0.39680997	0.63238308	-1.82537376

LCER(d18:0/24:1)+H	32361.9379	(24111.53, 40612.34)	37312.933	(29769.0, 44856.87)	0.39775548	0.63238308	1.15298821
TAG(46:1/FA18:1)+NH4	20958.5041	(16065.77, 25851.24)	26001.7992	(15269.15, 36734.45)	0.39898388	0.63238308	1.2406324
PG(18:2/16:1)-H	4182986.8	(2360590.3, 6005383.3)	5531605.2	(2969868.13, 8093342.26)	0.39901809	0.63238308	1.32240561
TAG(44:3/FA18:2)+NH4	8346.41357	(6103.64, 10589.18)	10000.6835	(6800.07, 13201.3)	0.401761	0.63238308	1.19820129
LPG(18:3)-H	56830.5916	(34036.21, 79624.97)	44651.1424	(29884.85, 59417.43)	0.40181025	0.63238308	-1.27276904
TAG(58:10/FA22:6)+NH4	11757.5934	(9082.95, 14432.23)	10131.5875	(7700.98, 12562.2)	0.40219564	0.63238308	-1.16048876
TAG(50:0/FA18:0)+NH4	16714.1691	(12087.1, 21341.24)	13522.2922	(7671.5, 19373.08)	0.4054129	0.63544342	-1.23604555
TAG(50:4/FA14:0)+NH4	12422.0656	(10050.08, 14794.05)	14231.3127	(10498.07, 17964.56)	0.40948539	0.63982093	1.14564784
TAG(48:1/FA18:1)+NH4	13015.1095	(5262.14, 20768.08)	9051.75794	(4412.92, 13690.6)	0.41392713	0.64388585	-1.43785434
TAG(56:5/FA18:1)+NH4	18173.3781	(15105.03, 21241.73)	15624.0215	(10085.83, 21162.21)	0.41466249	0.64388585	-1.16316903
TAG(54:3/FA20:3)+NH4	10257.3029	(7725.38, 12789.23)	8614.60867	(5586.54, 11642.68)	0.41988599	0.64997831	-1.19068704
TAG(48:2/FA14:0)+NH4	6558.27454	(5218.36, 7898.19)	5754.65176	(4371.61, 7137.69)	0.42140213	0.65031193	-1.13964751
TAG(48:0/FA18:0)+NH4	10182.7032	(8024.67, 12340.73)	11909.7817	(7956.03, 15863.53)	0.430179	0.66181384	1.16960904
TAG(48:4/FA20:4)+NH4	11646.8096	(9039.68, 14253.94)	10100.8498	(7336.23, 12865.47)	0.43219378	0.66182812	-1.15305245
TAG(44:0/FA16:0)+NH4	6141.04091	(4880.24, 7401.84)	6937.46839	(5422.61, 8452.32)	0.43283559	0.66182812	1.12968933
CE(20:5)+H	13983.222	(11185.15, 16781.3)	15932.3001	(11866.16, 19998.44)	0.43613621	0.66241753	1.13938691
TAG(54:1/FA18:1)+NH4	20127.2264	(8294.64, 31959.81)	14473.2379	(9104.75, 19841.72)	0.43636452	0.66241753	-1.39065125
TAG(50:4/FA18:2)+NH4	15050.1496	(11761.77, 18338.53)	13133.2473	(9736.34, 16530.16)	0.43719557	0.66241753	-1.14595799
TAG(56:7/FA20:3)+NH4	10529.973	(7701.43, 13358.51)	8948.5886	(6330.92, 11566.26)	0.43863301	0.66258763	-1.17671886

TAG(48:0/FA16:0)+NH4	27554.7044	(17866.96, 37242.45)	21181.6823	(8095.71, 34267.65)	0.44043379	0.6633039	-1.30087422
TAG(58:6/FA18:0)+NH4	20772.0483	(15153.74, 26390.36)	17736.4713	(12608.32, 22864.62)	0.44556669	0.66744418	-1.17114887
TAG(50:5/FA20:4)+NH4	22494.6366	(17489.77, 27499.5)	25826.1687	(18834.36, 32817.98)	0.44585271	0.66744418	1.1481034
TAG(54:3/FA20:1)+NH4	10865.6902	(8541.86, 13189.53)	9436.56625	(6594.26, 12278.88)	0.44772533	0.66824676	-1.15144533
TAG(52:3/FA16:1)+NH4	11728.3481	(8140.52, 15316.18)	9997.50555	(7655.69, 12339.32)	0.44936534	0.66869842	-1.17312744
TAG(52:7/FA20:5)+NH4	10065.9619	(7661.04, 12470.88)	8566.4948	(5504.51, 11628.48)	0.45131024	0.66959977	-1.17503858
TAG(55:2/FA18:1)+NH4	9441.05306	(7680.93, 11201.18)	10633.1052	(8001.1, 13265.11)	0.45679639	0.67573431	1.12626262
TAG(58:7/FA16:0)+NH4	10315.9006	(8041.23, 12590.57)	9138.95083	(7109.89, 11168.01)	0.45881835	0.67635541	-1.12878391
TAG(56:7/FA18:0)+NH4	10459.1137	(7892.62, 13025.61)	11879.942	(9223.13, 14536.75)	0.46190197	0.67635541	1.13584596
CE(18:1)+H	27494.3864	(18204.23, 36784.54)	32974.2813	(21794.55, 44154.01)	0.46251689	0.67635541	1.19930959
TAG(52:3/FA20:1)+NH4	8109.45144	(6213.92, 10004.98)	7182.8508	(5750.77, 8614.94)	0.4626271	0.67635541	-1.12900179
TAG(53:2/FA16:0)+NH4	12049.9753	(9738.85, 14361.1)	10019.2985	(4696.91, 15341.69)	0.46954096	0.6843191	-1.20267656
TAG(58:9/FA22:6)+NH4	10827.8347	(8754.21, 12901.46)	9411.00066	(6089.08, 12732.92)	0.47253476	0.6843191	-1.15055083
DAG(16:1/18:3)+NH4	50941.4486	(34222.61, 67660.29)	43297.8286	(32409.67, 54185.99)	0.47296178	0.6843191	-1.17653587
TAG(53:4/FA18:3)+NH4	8335.90253	(6791.87, 9879.94)	9750.5273	(5956.58, 13544.48)	0.47354882	0.6843191	1.16970265
TAG(56:4/FA20:4)+NH4	11291.1034	(9095.97, 13486.24)	10073.6579	(7543.82, 12603.49)	0.48054337	0.6924256	-1.12085436
TAG(58:10/FA20:5)+NH4	12003.5217	(9665.95, 14341.1)	10480.3723	(6827.35, 14133.39)	0.48612823	0.6984601	-1.14533352
TAG(44:2/FA16:1)+NH4	5883.16497	(4091.88, 7674.45)	6860.74637	(4740.28, 8981.21)	0.49293934	0.70621681	1.1661659
TAG(52:3/FA20:2)+NH4	8517.57319	(6608.52, 10426.62)	9818.7995	(6430.58, 13207.02)	0.49767374	0.70953632	1.15276961

PE(P-18:1/18:2)-H	52656286.9	(43392413.81, 61920159.91)	48226679.9	(40079781.62, 56373578.09)	0.49809449	0.70953632	-1.09184972
TAG(58:8/FA18:1)+NH4	14039.9394	(10097.44, 17982.44)	12032.9766	(8070.76, 15995.19)	0.50290694	0.71435645	-1.16678856
TAG(49:3/FA16:0)+NH4	8035.75411	(6180.34, 9891.17)	7079.8202	(4923.11, 9236.53)	0.5136609	0.72460533	-1.13502234
TAG(54:4/FA20:1)+NH4	12509.2262	(9449.75, 15568.7)	11086.0995	(8244.84, 13927.36)	0.51446953	0.72460533	-1.12837037
TAG(48:0/FA14:0)+NH4	8945.06212	(5421.79, 12468.34)	7495.57628	(5025.79, 9965.36)	0.51446979	0.72460533	-1.19337884
TAG(52:1/FA18:0)+NH4	24166.4068	(3649.24, 44683.57)	16537.7164	(9754.47, 23320.96)	0.51868111	0.7284847	-1.46129043
TAG(56:4/FA18:2)+NH4	10417.1626	(7383.63, 13450.69)	9074.02872	(6357.56, 11790.5)	0.52230116	0.73034527	-1.14801957
TAG(46:2/FA14:0)+NH4	7252.51404	(5899.23, 8605.8)	6370.07276	(3911.12, 8829.02)	0.52292721	0.73034527	-1.13852923
TAG(52:2/FA20:2)+NH4	10271.0469	(8179.88, 12362.22)	11821.1244	(7097.05, 16545.2)	0.53521525	0.74542514	1.15091718
TAG(53:4/FA16:0)+NH4	15157.6562	(9189.1, 21126.22)	13087.9538	(10217.32, 15958.59)	0.54514743	0.7568429	-1.15813796
TAG(58:7/FA22:6)+NH4	15938.8926	(11607.86, 20269.93)	14043.2085	(10054.57, 18031.85)	0.54677571	0.7568429	-1.13498939
PE(P-18:2/22:6)-H	600648.386	(508178.4, 693118.37)	563486.28	(495420.45, 631552.11)	0.54795426	0.7568429	-1.06595033
TAG(53:1/FA18:0)+NH4	11166.1079	(8187.52, 14144.7)	9628.87408	(5327.42, 13930.33)	0.55698453	0.76719633	-1.15964835
TAG(51:1/FA18:0)+NH4	10605.2564	(7602.82, 13607.69)	9491.5355	(7213.8, 11769.27)	0.5707449	0.77997773	-1.11733833
TAG(56:6/FA18:1)+NH4	14008.5828	(10698.37, 17318.8)	12326.5129	(7457.81, 17195.22)	0.57184916	0.77997773	-1.13645951
TAG(44:2/FA16:0)+NH4	8161.80665	(5806.51, 10517.1)	9515.72559	(5237.12, 13794.33)	0.57292748	0.77997773	1.16588471
PE(18:2/20:3)-H	2028442.25	(1597103.8, 2459780.7)	1805360	(1121698.37, 2489021.64)	0.57505536	0.77997773	-1.12356663

TAG(56:6/FA16:0) +NH4	19132.1202	(11833.66, 26430.58)	21724.0365	(17064.08, 26383.99)	0.57607235	0.77997773	1.1354746
TAG(49:2/FA18:1) +NH4	8171.24956	(5548.68, 10793.82)	7138.3515	(4753.7, 9523.0)	0.57667527	0.77997773	-1.144697
TAG(46:2/FA18:2) +NH4	14350.671	(10618.16, 18083.19)	16179.6474	(10849.54, 21509.75)	0.57876344	0.77997773	1.12744884
TAG(52:2/FA14:0) +NH4	7059.49404	(5557.54, 8561.45)	7725.60078	(5902.58, 9548.62)	0.58139343	0.77997773	1.09435616
TAG(52:2/FA18:1) +NH4	35595.24	(16257.58, 54932.9)	27233.9179	(13106.43, 41361.41)	0.5819518	0.77997773	-1.3070187
TAG(51:4/FA18:2) +NH4	8276.81998	(6590.73, 9962.91)	7334.41949	(4377.52, 10291.32)	0.58540898	0.77997773	-1.12849013
TAG(46:3/FA16:0) +NH4	9407.11218	(7122.16, 11692.06)	8406.63192	(5643.42, 11169.84)	0.58550938	0.77997773	-1.11901083
TAG(52:2/FA16:0) +NH4	23991.9556	(17030.86, 30953.05)	20508.9579	(9890.91, 31127.0)	0.58648665	0.77997773	-1.16982811
TAG(58:6/FA22:4) +NH4	16599.6907	(12455.23, 20744.15)	15063.7081	(11546.49, 18580.93)	0.58990922	0.77997773	-1.10196577
TAG(58:8/FA22:6) +NH4	19693.631	(15218.96, 24168.3)	21978.6548	(14582.49, 29374.82)	0.59019148	0.77997773	1.11602857
TAG(56:6/FA18:0) +NH4	13679.6907	(10370.47, 16988.91)	12160.2393	(7604.6, 16715.88)	0.59192	0.77997773	-1.12495242
LCER(d18:0/18:0) +H	27910.7691	(20036.96, 35784.58)	30731.7553	(24254.58, 37208.93)	0.59222684	0.77997773	1.10107161
TAG(52:1/FA16:0) +NH4	28524.4916	(10047.48, 47001.51)	21718.6127	(12583.58, 30853.65)	0.59278307	0.77997773	-1.31336619
TAG(56:3/FA18:0) +NH4	14971.8311	(11625.24, 18318.42)	13562.2376	(9536.34, 17588.14)	0.59889912	0.78505801	-1.10393517
TAG(46:1/FA16:0) +NH4	8348.67974	(6342.38, 10354.98)	9223.59322	(6618.15, 11829.03)	0.60024059	0.78505801	1.10479663
TAG(56:3/FA18:1) +NH4	19001.6284	(15764.3, 22238.96)	20759.6108	(14842.05, 26677.17)	0.60135443	0.78505801	1.09251746
TAG(55:3/FA18:1) +NH4	13332.195	(10310.46, 16353.93)	12039.1679	(8131.36, 15946.97)	0.60732141	0.7878492	-1.1074017
TAG(54:1/FA20:1) +NH4	12144.4772	(10326.87, 13962.09)	10985.712	(6790.83, 15180.59)	0.60747826	0.7878492	-1.1054793
TAG(52:4/FA18:2) +NH4	9998.44867	(7666.53, 12330.37)	8990.7591	(5906.83, 12074.69)	0.60832759	0.7878492	-1.11208059

TAG(54:6/FA18:2)+NH4	13741.6957	(10477.91, 17005.48)	12323.9718	(7937.05, 16710.89)	0.60979528	0.7878492	-1.11503791
TAG(42:1/FA16:1)+NH4	5940.06197	(4029.12, 7851.0)	7015.37695	(3255.91, 10774.84)	0.61419937	0.78976751	1.18102757
CE(22:4)+H	34684.1447	(25152.9, 44215.39)	31097.8381	(21183.88, 41011.8)	0.61585367	0.78976751	-1.11532334
TAG(48:4/FA18:1)+NH4	13450.2887	(10643.13, 16257.45)	12495.8574	(10310.83, 14680.88)	0.61624493	0.78976751	-1.07637981
TAG(52:5/FA20:3)+NH4	9807.42456	(7618.15, 11996.7)	8810.29613	(5367.13, 12253.46)	0.61895655	0.78976751	-1.11317763
DAG(18:2/22:5)+NH4	9547.50593	(8006.83, 11088.18)	10316.4767	(7687.08, 12945.88)	0.61917773	0.78976751	1.08054153
TAG(50:0/FA14:0)+NH4	8165.13941	(7038.74, 9291.54)	7582.30945	(5422.91, 9741.71)	0.62469871	0.79478208	-1.07686708
TAG(53:6/FA20:4)+NH4	10304.2375	(8029.13, 12579.34)	9266.18938	(5602.36, 12930.02)	0.62796902	0.796915	-1.11202535
TAG(54:1/FA20:0)+NH4	17370.313	(13369.93, 21370.7)	15873.5174	(11289.22, 20457.81)	0.63238837	0.80049161	-1.09429515
TAG(54:5/FA16:0)+NH4	13417.8341	(10103.73, 16731.94)	12189.872	(8272.04, 16107.71)	0.64125282	0.80966265	-1.10073625
TAG(52:0/FA18:0)+NH4	16976.8675	(5939.29, 28014.45)	13967.2587	(9042.08, 18892.44)	0.6455732	0.81306449	-1.21547598
TAG(50:2/FA14:0)+NH4	7470.52588	(6208.6, 8732.45)	6776.83021	(3794.94, 9758.72)	0.64916281	0.81553117	-1.10236285
TAG(54:4/FA16:0)+NH4	19986.725	(15887.53, 24085.92)	18586.7793	(14208.49, 22965.06)	0.65229896	0.81741725	-1.07531943
LPG(20:0)-H	989891.501	(594440.49, 1385342.51)	876640.405	(618244.47, 1135036.35)	0.65908351	0.82385438	-1.12918763
TAG(44:1/FA18:1)+NH4	12077.9205	(9043.4, 15112.44)	13333.0999	(8571.81, 18094.39)	0.66637514	0.8308917	1.10392348
TAG(46:2/FA16:1)+NH4	10104.8727	(6751.78, 13457.97)	11161.9087	(7610.33, 14713.49)	0.67468432	0.8391596	1.10460656
TAG(49:1/FA18:1)+NH4	7226.64268	(4877.52, 9575.77)	6552.60446	(4738.71, 8366.5)	0.67748148	0.83919796	-1.1028657
TAG(50:5/FA16:1)+NH4	12407.694	(10324.08, 14491.31)	13337.6597	(9333.1, 17342.22)	0.67807196	0.83919796	1.07495073

TAG(54:6/FA18:1)+NH4	10965.4381	(9041.38, 12889.49)	10243.9922	(7354.05, 13133.93)	0.67997191	0.83947149	-1.07042625
TAG(56:5/FA16:0)+NH4	17035.0488	(13144.79, 20925.31)	15530.1463	(9329.37, 21730.92)	0.68202461	0.83993178	-1.09690201
TAG(52:4/FA16:1)+NH4	9155.05527	(5759.24, 12550.87)	8187.33916	(5106.93, 11267.75)	0.69283668	0.84996945	-1.11819666
TAG(42:0/FA14:0)+NH4	5462.32084	(2926.59, 7998.06)	4756.98821	(2809.69, 6704.28)	0.69357507	0.84996945	-1.14827294
TAG(52:3/FA18:2)+NH4	13152.1347	(10923.98, 15380.29)	12249.4872	(7855.18, 16643.8)	0.6982152	0.85278993	-1.0736886
LCER(d18:0/26:1)+H	14630.0778	(11202.8, 18057.36)	13599.3545	(9689.03, 17509.68)	0.69928774	0.85278993	-1.07579208
TAG(47:0/FA17:0)+NH4	6778.38903	(5620.31, 7936.47)	7186.84458	(5320.86, 9052.83)	0.707809	0.85881511	1.0602585
TAG(40:0/FA14:0)+NH4	4585.31718	(3065.54, 6105.09)	5137.48073	(2612.63, 7662.33)	0.71200456	0.85881511	1.12041993
TAG(44:0/FA18:0)+NH4	8031.90848	(4174.41, 11889.41)	7008.40834	(3277.38, 10739.44)	0.71213637	0.85881511	-1.14603888
TAG(48:4/FA18:3)+NH4	7953.6526	(5689.56, 10217.74)	8612.7693	(5943.4, 11282.14)	0.71300511	0.85881511	1.08286969
TAG(48:2/FA16:0)+NH4	8267.60686	(6145.49, 10389.72)	8945.67409	(5938.64, 11952.71)	0.71388002	0.85881511	1.08201493
TAG(47:1/FA14:0)+NH4	8806.11484	(5960.27, 11651.96)	8128.3284	(5999.74, 10256.91)	0.71453417	0.85881511	-1.08338571
CE(20:1)+H	10873.5763	(6824.22, 14922.93)	11885.3292	(8513.5, 15257.16)	0.71778494	0.8606534	1.09304693
TAG(52:4/FA20:3)+NH4	9205.52923	(7705.41, 10705.65)	8617.32984	(5449.77, 11784.89)	0.73439219	0.87641126	-1.06825773
TAG(54:2/FA20:2)+NH4	10123.0727	(7566.97, 12679.18)	9479.05509	(6838.1, 12120.01)	0.73443264	0.87641126	-1.06794112
TAG(54:7/FA18:2)+NH4	8031.79005	(6452.74, 9610.84)	8842.97072	(3979.11, 13706.83)	0.73809472	0.8777023	1.10099625
CE(22:0)+H	8436.50276	(6438.42, 10434.58)	7940.7927	(5894.66, 9986.93)	0.7412349	0.8777023	-1.06242577
PE(14:0/20:5)-H	11883.5003	(8559.14, 15207.86)	12558.9558	(10490.85, 14627.06)	0.74159455	0.8777023	1.05683977
TAG(51:2/FA18:1)+NH4	11985.567	(8126.38, 15844.76)	10947.2206	(6082.21, 15812.23)	0.74253614	0.8777023	-1.09485023

TAG(50:3/FA16:0)+NH4	6700.21127	(4759.52, 8640.91)	7195.05224	(4885.94, 9504.17)	0.749078	0.8812428	1.07385453
TAG(54:8/FA22:6)+NH4	9097.80313	(6630.99, 11564.62)	8586.87951	(6928.66, 10245.1)	0.75098066	0.8812428	-1.0595005
PS(14:0/20:1)-H	703706.526	(564542.88, 842870.18)	656113.265	(386128.74, 926097.79)	0.75233985	0.8812428	-1.07253818
TAG(44:1/FA16:1)+NH4	5903.19653	(4685.66, 7120.73)	6302.54973	(3997.44, 8607.66)	0.75408311	0.8812428	1.06765033
DAG(18:1/22:4)+NH4	31531.6737	(21921.79, 41141.56)	29668.1964	(24039.83, 35296.56)	0.75434384	0.8812428	-1.0628106
TAG(50:4/FA18:1)+NH4	20003.052	(15728.69, 24277.41)	18946.1808	(13803.34, 24089.02)	0.75713213	0.88243838	-1.05578281
TAG(44:1/FA16:0)+NH4	5122.5874	(4060.21, 6184.97)	5470.06553	(3334.84, 7605.29)	0.76396116	0.88832693	1.06783254
DAG(14:0/18:2)+NH4	39747.1261	(27554.69, 51939.56)	37397.8031	(28646.79, 46148.81)	0.76721879	0.890045	-1.06281981
DCER(16:0)+H	221823.811	(160953.49, 282694.13)	208409.222	(142135.41, 274683.03)	0.77201884	0.89183151	-1.06436658
TAG(48:1/FA14:0)+NH4	12922.2412	(8437.73, 17406.76)	11906.5114	(6748.77, 17064.25)	0.77524107	0.89183151	-1.08530877
TAG(42:1/FA14:0)+NH4	4906.51392	(3888.09, 5924.94)	4660.05836	(3281.42, 6038.69)	0.77567191	0.89183151	-1.0528868
LPG(17:1)-Hstd.IS	489152.269	(382069.77, 596234.76)	464962.506	(336562.15, 593362.86)	0.77710064	0.89183151	-1.05202519
TAG(50:5/FA16:0)+NH4	15759.8601	(12434.98, 19084.74)	16582.4362	(11817.83, 21347.04)	0.77767707	0.89183151	1.05219438
TAG(52:4/FA22:4)+NH4	8000.23331	(6566.21, 9434.26)	8431.30179	(5624.03, 11238.58)	0.7824984	0.89449859	1.05388199
TAG(56:5/FA20:2)+NH4	13490.001	(9959.57, 17020.44)	12822.5916	(10018.44, 15626.74)	0.78536509	0.89449859	-1.0520495
LCER(24:0)+H	16605.6541	(12390.96, 20820.35)	15769.9508	(11683.97, 19855.94)	0.78536976	0.89449859	-1.0529934
TAG(52:5/FA18:1)+NH4	10931.0925	(8883.94, 12978.25)	11449.0636	(7973.52, 14924.61)	0.79728338	0.8985763	1.04738512
TAG(47:1/FA16:0)+NH4	6096.42373	(4230.83, 7962.02)	5795.25818	(4484.39, 7106.13)	0.79761673	0.8985763	-1.05196758
LPG(20:3)-H	4451920.48	(3711575.48, 5192265.49)	4585535.7	(3924359.88, 5246711.52)	0.79846695	0.8985763	1.03001294

TAG(49:0/FA17:0) +NH4	7643.273 6	(6088.12, 9198.43)	7177.733 44	(3718.86, 10636.61)	0.79935 489	0.89857 63	-1.064858 94
TAG(56:8/FA18:2) +NH4	11759.95 97	(8620.99, 14898.93)	11245.69 45	(9233.91, 13257.48)	0.79969 374	0.89857 63	-1.045729 96
LPG(22:5)-H	117109.4 68	(98201.74, 136017.19)	121430.4 5	(93032.7, 149828.2)	0.79973 291	0.89857 63	1.036896 95
TAG(52:6/FA20:5) +NH4	8181.856 87	(6054.31, 10309.41)	7865.221 51	(6669.59, 9060.86)	0.81830 432	0.91695 535	-1.040257 65
PE(14:0/20:1)-H	1398200. 61	(1178192.41, 1618208.8)	1433874. 27	(1233449.24, 1634299.3)	0.81975 808	0.91695 535	1.025513 98
TAG(50:4/FA18:3) +NH4	18086.30 39	(13545.32, 22627.28)	18992.36 83	(12235.23, 25749.51)	0.82372 826	0.91747 749	1.050096 72
TAG(53:3/FA18:2) +NH4	8849.036 08	(6363.6, 11334.47)	8444.788 34	(5945.66, 10943.91)	0.82389 479	0.91747 749	-1.047869 49
PS(16:0/20:2)-H	252651.1 15	(162800.84, 342501.39)	240750.7 55	(181215.79, 300285.72)	0.82625 738	0.91806 375	-1.049430 21
TAG(48:3/FA18:3) +NH4	12332.10 1	(8676.92, 15987.28)	12876.56 13	(9518.54, 16234.59)	0.83158 652	0.92193 628	1.044149 84
TAG(54:4/FA18:2) +NH4	14085.03 87	(10652.48, 17517.59)	13532.88 51	(9588.2, 17477.57)	0.83776 432	0.92673 044	-1.040800 88
TAG(44:2/FA14:0) +NH4	5947.795 91	(4773.05, 7122.54)	5758.701 21	(4335.26, 7182.14)	0.84147 586	0.92868 228	-1.032836 34
PE(O-16:0/18:0)-H	1390746. 75	(1043312.51, 1738181.0)	1464214. 38	(785986.76, 2142442.01)	0.84324 351	0.92868 228	1.052826 03
TAG(52:4/FA20:4) +NH4	8790.050 9	(7081.74, 10498.36)	8421.555 7	(4950.97, 11892.14)	0.85008 31	0.93324 123	-1.043756 19
TAG(52:2/FA18:2) +NH4	11256.95 88	(8314.63, 14199.28)	10743.78 46	(6164.81, 15322.76)	0.85111 601	0.93324 123	-1.047764 74
TAG(51:3/FA16:1) +NH4	8459.898 51	(6553.72, 10366.08)	8093.303 52	(4573.76, 11612.85)	0.85370 89	0.93403 599	-1.045296 09
TAG(52:6/FA18:2) +NH4	8087.254 35	(5971.01, 10203.49)	7807.850 4	(5681.58, 9934.12)	0.85707 285	0.93566 905	-1.035785
TAG(54:8/FA18:3) +NH4	8768.755 77	(7218.25, 10319.26)	8493.444 07	(5657.72, 11329.17)	0.86629 47	0.94367 614	-1.032414 61
TAG(54:5/FA18:3) +NH4	17549.16 54	(12213.35, 22884.98)	18212.60 52	(12419.68, 24005.53)	0.86991 025	0.94451 656	1.037804 63
TAG(52:3/FA18:3) +NH4	8560.963 59	(6116.22, 11005.7)	8256.774 14	(5548.57, 10964.97)	0.87109 934	0.94451 656	-1.036841 2

TAG(47:2/FA14:0)+NH4	8047.29878	(6182.81, 9911.79)	8277.50289	(6196.49, 10358.51)	0.8727333	0.94451656	1.02860638
TAG(54:3/FA18:2)+NH4	12036.6266	(8858.79, 15214.46)	11680.6004	(8774.44, 14586.76)	0.87503785	0.94496528	-1.03048013
TAG(55:5/FA20:4)+NH4	9385.53463	(7806.24, 10964.82)	9153.88127	(6439.69, 11868.08)	0.87961519	0.94786119	-1.02530657
TAG(54:3/FA18:1)+NH4	32441.4535	(21871.69, 43011.22)	34024.6671	(14339.01, 53710.32)	0.88318088	0.94965686	1.04880218
CE(20:0)+H	16462.7093	(10849.45, 22075.97)	15908.4558	(10752.69, 21064.22)	0.88883447	0.95292603	-1.03484018
TAG(54:3/FA20:2)+NH4	12135.5804	(9841.06, 14430.1)	12465.0284	(8142.38, 16787.67)	0.89047309	0.95292603	1.02714728
TAG(50:2/FA18:1)+NH4	8573.58323	(7158.05, 9989.12)	8773.36048	(6115.17, 11431.55)	0.89208933	0.95292603	1.02330149
DAG(18:2/22:6)+NH4	15173.5669	(10619.21, 19727.92)	14774.4944	(11620.95, 17928.04)	0.89384461	0.95292603	-1.02701091
DAG(18:0/18:2)+NH4	473282.152	(361525.16, 585039.15)	461680.28	(326805.44, 596555.12)	0.89678325	0.95293737	-1.02512967
TAG(52:4/FA20:2)+NH4	8178.00101	(6383.43, 9972.57)	7983.58365	(5605.29, 10361.87)	0.897667	0.95293737	-1.02435214
CE(20:2)+H	16537.5313	(11587.68, 21487.39)	16918.2635	(13603.35, 20233.18)	0.90412473	0.95652174	1.02302231
TAG(54:6/FA20:4)+NH4	9551.29812	(7285.44, 11817.15)	9264.85132	(5007.46, 13522.24)	0.90486957	0.95652174	-1.03091758
DAG(18:2/20:3)+NH4	23190.6649	(17372.85, 29008.48)	22718.1208	(17590.06, 27846.18)	0.90701521	0.9567671	-1.02080032
TAG(46:3/FA18:2)+NH4	21991.9588	(17112.88, 26871.03)	22471.2224	(15579.46, 29362.98)	0.91587599	0.96407999	1.02179267
TAG(54:8/FA20:5)+NH4	9320.38184	(6895.0, 11745.76)	8981.41245	(2444.99, 15517.83)	0.92088364	0.96574735	-1.03774121
TAG(54:6/FA22:6)+NH4	8362.34693	(6373.83, 10350.87)	8193.75151	(5469.79, 10917.71)	0.92132297	0.96574735	-1.0205761
TAG(44:0/FA14:0)+NH4	6401.1235	(4318.08, 8484.17)	6549.45476	(4411.61, 8687.3)	0.92376333	0.96627963	1.02317269
TAG(46:1/FA16:1)+NH4	7939.00879	(5974.48, 9903.54)	8086.88454	(5613.22, 10560.55)	0.92677001	0.96692073	1.01862648
TAG(56:6/FA20:5)+NH4	18056.7117	(15480.51, 20632.91)	17786.1003	(12273.81, 23298.39)	0.9282439	0.96692073	-1.01521476

CE(22:1)+H	10542.3236	(8244.65, 12840.0)	10705.7036	(6945.64, 14465.77)	0.94097081	0.97814014	1.01549753
TAG(48:2/FA18:0)+NH4	11502.1443	(8442.69, 14561.6)	11350.1492	(8352.61, 14347.69)	0.94545278	0.97895722	-1.01339147
TAG(47:2/FA16:1)+NH4	10484.9996	(7711.53, 13258.47)	10351.9416	(7630.34, 13073.54)	0.9476732	0.97895722	-1.01285343
TAG(46:0/FA14:0)+NH4	9789.73397	(6386.6, 13192.87)	9949.6588	(6614.01, 13285.31)	0.94833091	0.97895722	1.01633597
DCER(20:0)+H	229214.509	(181642.28, 276786.74)	231387.949	(185148.06, 277627.84)	0.94958851	0.97895722	1.00948212
TAG(56:5/FA20:1)+NH4	10028.5077	(7441.03, 12615.99)	10133.2036	(7468.02, 12798.39)	0.95635963	0.98390909	1.01043983
TAG(56:5/FA18:2)+NH4	12369.569	(8819.17, 15919.97)	12171.5614	(5381.74, 18961.38)	0.95918571	0.98479026	-1.01626806
TAG(52:1/FA18:1)+NH4	20894.2176	(6873.87, 34914.56)	21395.598	(6442.58, 36348.62)	0.9622751	0.9859376	1.02399613
TAG(56:5/FA20:4)+NH4	16274.69	(11847.17, 20702.21)	16427.1032	(10538.59, 22315.62)	0.96733189	0.98721077	1.00936504
TAG(50:4/FA20:4)+NH4	13794.5973	(10419.48, 17169.72)	13685.6449	(9696.84, 17674.45)	0.96746655	0.98721077	-1.00796107
TAG(50:6/FA20:4)+NH4	10844.198	(8366.05, 13322.35)	10769.7524	(7647.05, 13892.45)	0.97067618	0.98792393	-1.00691247
TAG(44:2/FA18:2)+NH4	10807.698	(7088.36, 14527.04)	10897.9361	(7575.35, 14220.52)	0.97211715	0.98792393	1.00834943
TAG(53:2/FA18:2)+NH4	8286.51587	(6122.05, 10450.99)	8230.02015	(5472.03, 10988.01)	0.97472618	0.9885661	-1.00686459
TAG(52:4/FA18:0)+NH4	7066.90962	(5617.4, 8516.42)	7091.43331	(5105.76, 9077.11)	0.98419617	0.99491969	1.00347021
TAG(48:1/FA16:1)+NH4	9533.45537	(6004.98, 13061.93)	9572.24331	(6825.63, 12318.86)	0.98669262	0.99491969	1.00406861
TAG(55:4/FA18:2)+NH4	11361.7375	(9142.77, 13580.71)	11410.1839	(5429.3, 17391.07)	0.98696033	0.99491969	1.004264
TAG(48:1/FA16:0)+NH4	14661.8103	(9903.87, 19419.75)	14635.4449	(11167.52, 18103.37)	0.99335059	0.99741623	-1.00180148
TAG(49:0/FA16:0)+NH4	10334.0866	(5769.53, 14898.64)	10362.7167	(5375.36, 15350.07)	0.99342657	0.99741623	1.00277046
TAG(58:10/FA22:5)+NH4	8925.21654	(6720.28, 11130.15)	8933.53635	(6505.68, 11361.39)	0.99606009	0.9980562	1.00093217
PG(18:2/18:3)-H	131755.419	(92854.42, 170656.42)	131860.231	(46992.91, 216727.55)	0.99818452	0.99818452	1.00079551

Table S4: The list of lipids showing statistically significant concentration change ($p<0.05$; $q<0.05$) when Mild-AD sufferers compared to AD patients. rp0, Mild-AD; Grp1, AD.

AD	Grp0_Mean	Grp0_Mean-95CI	Grp1_Mean	Grp1_Mean-95CI	Ttest P value	Q value	Fold Change
PE(P-18:0/18:1)-H	310063474	(257939930.78, 362187016.69)	885322154	(823446292.93, 947198014.43)	6.0104E-14	3.0052E-11	2.85529328
DAG(14:0/14:0)+NH4	197071.739	(142817.1, 251326.38)	1064956.99	(935410.11, 1194503.87)	3.9287E-12	8.0362E-10	5.40390518
PS(18:1/20:4)-H	232909.511	(139887.65, 325931.37)	830760.351	(780446.06, 881074.64)	4.8217E-12	8.0362E-10	3.56688032
SM(26:0)+H	2644497.15	(1882090.01, 3406904.29)	34533553.5	(28721794.56, 40345312.35)	3.1833E-11	3.9791E-09	13.0586465
PS(18:1/18:2)-H	120039.891	(84567.29, 155512.49)	492963.939	(434691.99, 551235.88)	4.5407E-11	4.0149E-09	4.10666768
PS(18:1/20:5)-H	42429.2965	(28666.74, 56191.85)	469422.634	(407236.96, 531608.31)	4.8179E-11	4.0149E-09	11.0636441
PE(P-18:0/20:3)-H	32462195.7	(25969733.0, 38954658.49)	72933837.3	(67979933.83, 77887740.75)	1.3451E-10	9.6077E-09	2.24673149
PS(14:0/22:6)-H	751688.158	(650581.51, 852794.81)	60558.9266	(26412.5, 94705.35)	2.1182E-10	1.3238E-08	-12.412508
TAG(56:7/FA20:4)+NH4	8414.82118	(6296.73, 10532.91)	29795.3837	(26138.78, 33451.99)	2.6326E-10	1.4626E-08	3.54082197
TAG(51:3/FA17:0)+NH4	5607.66839	(4335.48, 6879.86)	24229.1248	(20732.24, 27726.01)	4.8048E-10	2.4024E-08	4.32071285
PE(P-18:0/20:1)-H	81478524.3	(63715787.13, 99241261.49)	210066447	(190216080.42, 229916814.3)	7.3703E-10	3.3501E-08	2.57818179
TAG(56:9/FA20:5)+NH4	7050.70674	(5184.29, 8917.12)	27200.4941	(23053.46, 31347.53)	2.3591E-09	9.8294E-08	3.85783938
TAG(51:2/FA17:0)+NH4	6068.06483	(4403.79, 7732.34)	29582.0114	(24501.06, 34662.97)	2.5631E-09	9.8579E-08	4.8750322
DAG(16:1/18:3)+NH4	43297.8286	(32409.67, 54185.99)	125728.722	(110770.31, 140687.13)	2.952E-09	1.0543E-07	2.90381125
PS(16:0/22:5)-H	455560.987	(249103.57, 662018.4)	3938038.42	(3192243.99, 4683832.86)	4.9063E-09	1.5695E-07	8.64437153
TAG(56:7/FA18:1)+NH4	8497.22736	(5651.35, 11343.1)	36341.7988	(30986.19, 41697.41)	5.0223E-09	1.5695E-07	4.27690084
TAG(58:5/FA18:1)+NH4	7660.7731	(5653.14, 9668.4)	32299.7676	(27025.55, 37573.99)	7.8476E-09	2.3081E-07	4.21625431
PS(18:0/18:3)-H	194764.211	(147103.89, 242424.53)	686286.981	(587853.66, 784720.31)	8.5429E-09	2.373E-07	3.52368117
PE(P-18:0/20:2)-H	25224862	(18924944.92, 31524779.04)	63031870.8	(56207610.98, 69856130.64)	2.0565E-08	5.4119E-07	2.49879943
TAG(58:10/FA20:5)+NH4	10480.3723	(6827.35, 14133.39)	51161.7831	(41871.01, 60452.55)	2.9626E-08	7.4064E-07	4.88167611
TAG(58:6/FA18:1)+NH4	10731.4659	(7497.18, 13965.76)	34262.7863	(29214.7, 39310.88)	4.1326E-08	9.7851E-07	3.19274055
TAG(58:8/FA20:3)+NH4	7591.7812	(5560.54, 9623.02)	25353.7469	(21354.64, 29352.85)	4.3055E-08	9.7851E-07	3.33963088
TAG(58:9/FA22:5)+NH4	5750.42499	(4395.61, 7105.24)	19451.6886	(16253.86, 22649.51)	5.2401E-08	1.1392E-06	3.38265235

TAG(52:7/FA18:1)+NH4	7760.41074	(6016.32, 9504.5)	31529.6644	(25751.94, 37307.39)	6.3451E-08	1.2926E-06	4.06288603
PE(P-18:0/18:3)-H	119241.055	(69034.07, 169448.05)	682081.863	(533689.88, 830473.85)	6.4632E-08	1.2926E-06	5.72019311
TAG(58:7/FA18:1)+NH4	14609.6718	(11183.8, 18035.54)	81613.7682	(64957.33, 98270.21)	6.9384E-08	1.3343E-06	5.58628349
DAG(18:0/18:3)+NH4	107800.366	(80086.5, 135514.23)	313850.206	(267407.85, 360292.56)	7.7239E-08	1.4304E-06	2.91140205
DAG(14:0/18:2)+NH4	37397.8031	(28646.79, 46148.81)	104825.123	(89396.07, 120254.18)	8.4291E-08	1.5052E-06	2.80297543
TAG(56:2/FA20:0)+NH4	8007.67449	(5828.75, 10186.6)	54504.2199	(42214.75, 66793.69)	8.7577E-08	1.5099E-06	6.80649793
TAG(48:4/FA18:3)+NH4	8612.7693	(5943.4, 11282.14)	28750.2472	(24119.93, 33380.56)	9.7795E-08	1.6299E-06	3.33809559
TAG(50:4/FA20:3)+NH4	14716.1162	(10713.32, 18718.92)	63241.5512	(51144.24, 75338.86)	1.1393E-07	1.8375E-06	4.29743489
TAG(49:2/FA16:1)+NH4	6237.62103	(4214.75, 8260.49)	20225.1497	(17161.0, 23289.3)	1.2535E-07	1.9279E-06	3.24244606
TAG(58:7/FA18:2)+NH4	12720.6685	(10027.1, 15414.24)	33156.8072	(28359.9, 37953.72)	1.2961E-07	1.9279E-06	2.60653024
TAG(49:3/FA18:3)+NH4	3893.66632	(2821.2, 4966.13)	12969.5104	(10788.14, 15150.88)	1.3109E-07	1.9279E-06	3.33092498
TAG(54:6/FA16:0)+NH4	11350.3049	(8348.12, 14352.49)	51345.1984	(43076.6, 59613.8)	1.6833E-07	2.4047E-06	4.5236845
TAG(52:6/FA18:1)+NH4	7135.35408	(5675.93, 8594.78)	25764.2095	(20927.03, 30601.38)	2.1109E-07	2.9318E-06	3.6107822
PS(18:2/18:2)-H	129801.903	(101257.55, 158346.25)	491886.079	(426027.78, 557744.38)	2.2181E-07	2.9974E-06	3.78951362
PE(O-18:0/20:1)-H	14964407.4	(10905571.77, 19023242.94)	33522055.8	(29907542.42, 37136569.14)	2.3118E-07	3.0418E-06	2.24011917
TAG(49:1/FA16:0)+NH4	7423.03805	(5515.16, 9330.91)	28158.3997	(23026.96, 33289.84)	2.5504E-07	3.1057E-06	3.79337941
TAG(56:6/FA20:5)+NH4	17786.1003	(12273.81, 23298.39)	57974.7788	(48182.77, 67766.79)	2.5507E-07	3.1057E-06	3.25955537
PS(18:0/16:1)-H	1448291.34	(1299054.13, 1597528.55)	5288121.63	(4302575.95, 6273667.32)	2.5881E-07	3.1057E-06	3.65128306
PS(18:1/20:3)-H	77814.8576	(57035.11, 98594.61)	198171.036	(171927.93, 224414.14)	2.6088E-07	3.1057E-06	2.5466992
TAG(58:8/FA20:4)+NH4	19752.4513	(14365.15, 25139.75)	65236.6502	(54207.15, 76266.15)	2.9673E-07	3.4503E-06	3.3027116
PS(16:0/20:4)-H	161359.011	(88519.97, 234198.06)	2751902.68	(2260779.8, 3243025.57)	3.1692E-07	3.5624E-06	17.0545336
TAG(49:2/FA18:1)+NH4	7138.3515	(4753.7, 9523.0)	34566.6262	(27318.05, 41815.2)	3.2062E-07	3.5624E-06	4.8423822
TAG(58:6/FA22:4)+NH4	15063.7081	(11546.49, 18580.93)	43653.322	(36369.52, 50937.12)	3.5202E-07	3.8263E-06	2.89791344
TAG(52:6/FA20:4)+NH4	7326.22972	(5503.8, 9148.66)	23069.5281	(19177.49, 26961.56)	3.7656E-07	4.006E-06	3.148895
TAG(50:1/FA20:1)+NH4	4171.49496	(2927.24, 5415.75)	22142.4746	(17457.79, 26827.16)	4.3779E-07	4.5603E-06	5.30804299

TAG(58:10/FA20:4)+NH4	7880.89804	(5384.01, 10377.78)	43395.6764	(33230.69, 53560.66)	4.6068E-07	4.7009E-06	5.506438
TAG(47:0/FA14:0)+NH4	4406.33063	(3223.11, 5589.55)	13566.0532	(11175.38, 15956.73)	5.6997E-07	5.6997E-06	3.07876425
TAG(51:2/FA16:0)+NH4	8658.56573	(6153.56, 11163.57)	32572.123	(26038.9, 39105.34)	7.1504E-07	6.938E-06	3.76183817
TAG(50:3/FA18:3)+NH4	5540.97981	(4339.87, 6742.09)	16616.3252	(13733.08, 19499.57)	7.2155E-07	6.938E-06	2.99880631
TAG(48:2/FA16:1)+NH4	5564.96946	(3917.05, 7212.89)	24321.1239	(19293.82, 29348.43)	8.4449E-07	7.9668E-06	4.37039665
TAG(56:8/FA20:5)+NH4	9090.24746	(6289.14, 11891.36)	23116.2503	(19831.23, 26401.27)	8.8563E-07	8.2003E-06	2.54297261
TAG(48:3/FA16:1)+NH4	7172.84029	(4682.36, 9663.32)	19813.2196	(16891.28, 22735.15)	1.0224E-06	9.2945E-06	2.76225579
LCER(26:1)+H	8498.7705	(6171.83, 10825.71)	37307.3542	(29057.53, 45557.17)	1.0598E-06	9.4625E-06	4.38973545
TAG(58:3/FA18:1)+NH4	6195.18011	(4300.16, 8090.2)	19425.1185	(15871.87, 22978.37)	1.1367E-06	9.9707E-06	3.13552119
TAG(60:11/FA22:6)+NH4	8335.89434	(6169.5, 10502.28)	30085.0778	(23648.05, 36522.1)	1.1797E-06	1.017E-05	3.60910019
PE(O-18:0/22:5)-H	9698441.67	(8171143.65, 11225739.69)	15284484.2	(14267826.08, 16301142.39)	1.3836E-06	1.1532E-05	1.57597321
TAG(54:7/FA16:1)+NH4	9477.77255	(6253.18, 12702.37)	28901.6647	(23796.64, 34006.69)	1.4041E-06	1.1532E-05	3.04941531
TAG(58:6/FA18:0)+NH4	17736.4713	(12608.32, 22864.62)	98931.1181	(74986.02, 122876.22)	1.4069E-06	1.1532E-05	5.57783543
TAG(52:4/FA22:4)+NH4	8431.30179	(5624.03, 11238.58)	22155.355	(18910.66, 25400.05)	1.5934E-06	1.285E-05	2.62775021
TAG(48:1/FA18:0)+NH4	7600.06828	(5472.8, 9727.33)	23986.3676	(19778.34, 28194.4)	1.6333E-06	1.2913E-05	3.15607264
TAG(49:0/FA17:0)+NH4	7177.73344	(3718.86, 10636.61)	21962.0582	(18726.48, 25197.63)	1.6529E-06	1.2913E-05	3.05974837
TAG(58:7/FA22:5)+NH4	14793.4778	(11887.59, 17699.36)	70882.5426	(54828.08, 86937.0)	1.6847E-06	1.2941E-05	4.79147254
TAG(51:4/FA20:4)+NH4	5745.84182	(4343.55, 7148.14)	15932.286	(13208.49, 18656.08)	1.7081E-06	1.2941E-05	2.77283756
TAG(57:2/FA18:1)+NH4	6266.58764	(4564.75, 7968.43)	18134.4458	(14824.95, 21443.94)	1.8947E-06	1.4139E-05	2.89383104
TAG(53:2/FA18:1)+NH4	8034.60825	(6332.46, 9736.76)	23629.0451	(19262.64, 27995.45)	2.123E-06	1.5496E-05	2.94090818
TAG(53:6/FA20:4)+NH4	9266.18938	(5602.36, 12930.02)	29147.87	(24112.34, 34183.4)	2.1385E-06	1.5496E-05	3.14561562
TAG(52:5/FA22:5)+NH4	5709.14193	(4156.27, 7262.01)	16936.7944	(13751.56, 20122.03)	2.1773E-06	1.5552E-05	2.96660944
TAG(47:1/FA16:0)+NH4	5795.25818	(4484.39, 7106.13)	18859.2314	(14828.36, 22890.1)	2.2269E-06	1.5683E-05	3.25425215
TAG(48:2/FA18:0)+NH4	11350.1492	(8352.61, 14347.69)	29702.4663	(24816.74, 34588.19)	2.559E-06	1.7771E-05	2.61692299
SM(18:0)+H	1347643.34	(918308.85, 1776977.82)	9024497.68	(6550965.05, 11498030.3)	2.7043E-06	1.8522E-05	6.69650303

TAG(52:3/FA16:1)+NH4	9997.50555	(7655.69, 12339.32)	49843.8383	(37260.5, 62427.17)	2.749E-06	1.8574E-05	4.98562747
HCER(d18:0/18:0)+H	1798.98704	(1399.8, 2198.18)	26362.7952	(18056.87, 34668.72)	3.2267E-06	2.1512E-05	14.6542441
DCER(18:1)+H	31763.5472	(22749.81, 40777.28)	102054.953	(81240.43, 122869.48)	3.3101E-06	2.1777E-05	3.21295831
TAG(54:8/FA20:4)+NH4	8093.48469	(6177.32, 10009.65)	21270.5066	(17594.13, 24946.89)	3.4249E-06	2.224E-05	2.62810241
TAG(54:2/FA18:0)+NH4	14507.6713	(9982.62, 19032.72)	44233.8917	(35641.32, 52826.46)	3.5688E-06	2.2877E-05	3.04900012
TAG(52:5/FA16:1)+NH4	5273.6472	(3959.28, 6588.02)	21033.8911	(15934.99, 26132.8)	3.6761E-06	2.3266E-05	3.98849038
TAG(53:2/FA17:0)+NH4	8466.75833	(5358.45, 11575.06)	40880.5524	(31120.73, 50640.38)	3.8322E-06	2.3792E-05	4.82835943
TAG(52:6/FA16:1)+NH4	5765.29035	(4049.08, 7481.5)	16000.3547	(13090.8, 18909.91)	3.8543E-06	2.3792E-05	2.77529034
TAG(52:2/FA18:0)+NH4	8660.29265	(5203.51, 12117.08)	24011.6177	(20088.77, 27934.46)	4.416E-06	2.6927E-05	2.77261043
TAG(56:2/FA20:1)+NH4	9382.32826	(6578.12, 12186.54)	23605.8595	(19808.16, 27403.56)	4.6952E-06	2.796E-05	2.51599164
TAG(48:3/FA18:2)+NH4	13055.5236	(7895.92, 18215.12)	44081.0473	(35098.97, 53063.13)	4.6973E-06	2.796E-05	3.3764289
TAG(58:6/FA16:0)+NH4	6568.25081	(4488.3, 8648.2)	22102.3761	(17568.96, 26635.79)	5.2306E-06	3.0768E-05	3.3650323
TAG(54:5/FA20:3)+NH4	7633.76384	(6121.41, 9146.12)	23214.8309	(18299.89, 28129.77)	5.3739E-06	3.1243E-05	3.04107271
TAG(54:0/FA16:0)+NH4	9345.55721	(6197.22, 12493.89)	27989.101	(22317.22, 33660.98)	5.8188E-06	3.3204E-05	2.99490981
TAG(50:1/FA14:0)+NH4	6240.42445	(4552.59, 7928.26)	18540.2649	(14932.76, 22147.77)	5.8439E-06	3.3204E-05	2.9709942
DAG(16:1/20:2)+NH4	114568.692	(91076.87, 138060.51)	229661.887	(198665.97, 260657.8)	6.4677E-06	3.6335E-05	2.00457806
TAG(56:8/FA18:3)+NH4	11637.9149	(9019.52, 14256.31)	33977.8627	(26763.81, 41191.91)	7.0084E-06	3.8936E-05	2.91958337
TAG(48:4/FA18:1)+NH4	12495.8574	(10310.83, 14680.88)	40459.8737	(31741.02, 49178.72)	7.2631E-06	3.9907E-05	3.23786295
TAG(60:11/FA22:5)+NH4	11973.9223	(8928.07, 15019.77)	41720.7771	(32097.15, 51344.4)	8.0895E-06	4.3604E-05	3.4843033
TAG(51:1/FA18:0)+NH4	9491.5355	(7213.8, 11769.27)	21478.7384	(17818.9, 25138.58)	8.1103E-06	4.3604E-05	2.2629361
DCER(16:0)+H	208409.222	(142135.41, 274683.03)	496542.592	(418723.9, 574361.29)	8.3312E-06	4.4315E-05	2.38253656
TAG(49:1/FA18:1)+NH4	6552.60446	(4738.71, 8366.5)	30646.8943	(23394.85, 37898.94)	8.6018E-06	4.4615E-05	4.67705544
TAG(58:7/FA16:0)+NH4	9138.95083	(7109.89, 11168.01)	20517.3233	(17240.34, 23794.3)	8.6487E-06	4.4615E-05	2.24504144
HCER(d18:0/20:0)+H	12202.4176	(9500.32, 14904.52)	225288.39	(148370.33, 302206.45)	8.6552E-06	4.4615E-05	18.4626029
TAG(58:6/FA22:5)+NH4	13975.7358	(9347.68, 18603.79)	46100.333	(36037.88, 56162.79)	8.7724E-06	4.4757E-05	3.29859792

TAG(56:1/FA18:1)+NH4	8584.16427	(4816.74, 12351.59)	31064.1271	(24202.28, 37925.97)	9.1302E-06	4.6112E-05	3.61877127
TAG(54:1/FA16:0)+NH4	10530.9799	(7195.34, 13866.62)	28350.7872	(23046.38, 33655.19)	9.5527E-06	4.7763E-05	2.69213193
TAG(60:10/FA22:5)+NH4	9104.39331	(7105.15, 11103.63)	36190.9506	(27149.35, 45232.55)	9.7853E-06	4.8442E-05	3.97510843
TAG(55:5/FA18:1)+NH4	14406.0263	(10789.23, 18022.82)	37307.2302	(30127.5, 44486.96)	1.0851E-05	5.2965E-05	2.58969611
TAG(52:3/FA20:0)+NH4	7268.36689	(5108.62, 9428.11)	22590.3814	(17691.58, 27489.18)	1.0911E-05	5.2965E-05	3.10804088
TAG(52:7/FA22:6)+NH4	9379.57285	(6590.7, 12168.45)	27030.6656	(21919.43, 32141.91)	1.1491E-05	5.4364E-05	2.88186531
TAG(55:5/FA18:2)+NH4	9952.94538	(7258.43, 12647.46)	28003.7252	(22488.34, 33519.11)	1.1554E-05	5.4364E-05	2.81361186
TAG(48:5/FA18:2)+NH4	12168.9687	(8479.33, 15858.6)	28745.2465	(24181.11, 33309.38)	1.1688E-05	5.4364E-05	2.36217606
TAG(49:0/FA18:0)+NH4	7081.11438	(5666.96, 8495.27)	30161.7308	(22654.3, 37669.16)	1.1863E-05	5.4364E-05	4.25946104
TAG(55:1/FA16:0)+NH4	7322.91042	(5807.01, 8838.81)	21417.2596	(16440.88, 26393.64)	1.188E-05	5.4364E-05	2.92469229
TAG(60:10/FA22:6)+NH4	11227.6651	(7402.01, 15053.32)	45344.0934	(34550.28, 56137.91)	1.1886E-05	5.4364E-05	4.03860401
TAG(56:10/FA18:2)+NH4	8386.29053	(5936.32, 10836.26)	23021.6707	(18627.42, 27415.92)	1.196E-05	5.4364E-05	2.74515539
TAG(50:3/FA18:0)+NH4	8528.78487	(6389.69, 10667.88)	22925.7214	(18698.01, 27153.43)	1.2168E-05	5.4811E-05	2.68804076
TAG(52:3/FA14:0)+NH4	6301.55706	(4552.0, 8051.11)	17949.1691	(14371.18, 21527.15)	1.2494E-05	5.5567E-05	2.84837047
TAG(52:8/FA16:1)+NH4	5318.33261	(3183.27, 7453.39)	14888.4888	(12150.63, 17626.34)	1.2558E-05	5.5567E-05	2.79946552
TAG(54:5/FA18:1)+NH4	10752.0534	(7442.16, 14061.95)	28640.8059	(23157.57, 34124.04)	1.2809E-05	5.618E-05	2.66375221
TAG(58:9/FA20:4)+NH4	9879.0507	(7689.61, 12068.49)	35579.9678	(26872.6, 44287.34)	1.3097E-05	5.6946E-05	3.60155736
TAG(52:2/FA20:0)+NH4	7780.98163	(5725.16, 9836.8)	18920.1157	(15617.98, 22222.25)	1.3326E-05	5.7441E-05	2.43158467
TAG(54:4/FA20:2)+NH4	7519.79624	(5559.64, 9479.96)	20375.5587	(16407.8, 24343.32)	1.3629E-05	5.8243E-05	2.7095892
TAG(49:1/FA14:0)+NH4	3876.04299	(3112.04, 4640.05)	13566.6088	(10257.13, 16876.09)	1.395E-05	5.8666E-05	3.50011825
TAG(48:3/FA18:1)+NH4	12829.4815	(8587.19, 17071.77)	30782.779	(25736.03, 35829.53)	1.3963E-05	5.8666E-05	2.39937827
DAG(16:0/20:5)+NH4	57727.773	(41894.96, 73560.59)	171473.594	(134227.58, 208719.61)	1.4426E-05	6.0106E-05	2.97038298
TAG(52:3/FA18:0)+NH4	6823.2558	(5139.88, 8506.63)	17611.0244	(14129.16, 21092.88)	1.5048E-05	6.2183E-05	2.58102949
TAG(56:5/FA18:1)+NH4	15624.0215	(10085.83, 21162.21)	43317.9885	(35150.17, 51485.81)	1.6075E-05	6.5882E-05	2.77252488
TAG(54:5/FA18:3)+NH4	18212.6052	(12419.68, 24005.53)	60923.6345	(46738.14, 75109.12)	1.6519E-05	6.7151E-05	3.34513563

TAG(52:4/FA18:3)+NH4	8490.69876	(5641.02, 11340.38)	30585.7835	(23191.93, 37979.64)	1.684E-05	6.7905E-05	3.60226931
TAG(47:1/FA16:1)+NH4	7020.97079	(5564.97, 8476.97)	19319.534	(15155.25, 23483.82)	1.7502E-05	7.0009E-05	2.75168985
TAG(54:2/FA18:2)+NH4	6076.21698	(4556.85, 7595.59)	19200.0958	(14741.93, 23658.26)	1.7781E-05	7.0561E-05	3.1598766
TAG(56:1/FA16:0)+NH4	7821.44259	(5539.08, 10103.8)	19192.1219	(15807.96, 22576.29)	1.7942E-05	7.0639E-05	2.45378288
TAG(56:3/FA18:2)+NH4	8336.84245	(5783.91, 10889.78)	20032.7719	(16407.63, 23657.91)	1.8808E-05	7.347E-05	2.40292077
TAG(52:2/FA16:1)+NH4	8659.83176	(5773.14, 11546.53)	27075.6985	(21253.38, 32898.01)	1.9241E-05	7.4579E-05	3.12658481
TAG(52:3/FA18:1)+NH4	12036.1817	(8785.48, 15286.89)	30253.559	(24397.51, 36109.61)	1.9782E-05	7.5549E-05	2.5135512
TAG(56:4/FA22:4)+NH4	6549.64413	(4752.23, 8347.06)	13722.1187	(11694.93, 15749.31)	1.9794E-05	7.5549E-05	2.09509378
TAG(52:1/FA20:1)+NH4	8781.00143	(6140.31, 11421.69)	19678.5887	(16618.24, 22738.94)	2.0548E-05	7.7833E-05	2.24104151
CE(20:0)+H	15908.4558	(10752.69, 21064.22)	38824.2999	(31935.15, 45713.45)	2.1217E-05	7.9602E-05	2.44048199
TAG(54:1/FA20:0)+NH4	15873.5174	(11289.22, 20457.81)	43764.5139	(34935.63, 52593.4)	2.1451E-05	7.9602E-05	2.75707727
TAG(56:6/FA16:0)+NH4	21724.0365	(17064.08, 26383.99)	121577.136	(83507.87, 159646.4)	2.1492E-05	7.9602E-05	5.59643398
TAG(53:4/FA20:4)+NH4	5659.4884	(4559.0, 6759.98)	16699.88	(13182.86, 20216.9)	2.1713E-05	7.9828E-05	2.95077554
TAG(58:9/FA18:2)+NH4	9881.35467	(7063.76, 12698.95)	24308.1105	(19729.02, 28887.2)	2.2278E-05	8.1308E-05	2.45999778
TAG(50:2/FA18:1)+NH4	8773.36048	(6115.17, 11431.55)	27009.1566	(21097.34, 32920.97)	2.2925E-05	8.3063E-05	3.07854176
TAG(50:3/FA20:3)+NH4	7110.01334	(5336.55, 8883.47)	34259.144	(24999.05, 43519.24)	2.3194E-05	8.3431E-05	4.81843597
TAG(54:6/FA20:5)+NH4	11888.3656	(8179.53, 15597.2)	44070.957	(32812.07, 55329.84)	2.581E-05	9.1799E-05	3.7070661
HCER(18:1)+H	228234.637	(130349.62, 326119.65)	3757975.52	(2651065.43, 4864885.61)	2.5887E-05	9.1799E-05	16.4654041
TAG(52:5/FA18:3)+NH4	6697.21597	(4538.85, 8855.58)	24164.6447	(17662.63, 30666.66)	2.7937E-05	9.8054E-05	3.60816267
TAG(52:1/FA16:1)+NH4	5819.87147	(4285.56, 7354.19)	16639.8543	(12764.3, 20515.41)	2.81E-05	9.8054E-05	2.85914464
DAG(18:2/18:3)+NH4	20066.2063	(14373.78, 25758.63)	52284.2971	(41572.57, 62996.03)	2.8239E-05	9.8054E-05	2.60558953
TAG(58:8/FA22:5)+NH4	7899.31771	(5540.27, 10258.36)	20863.7692	(16732.98, 24994.56)	2.8923E-05	9.9558E-05	2.64121155
TAG(50:2/FA20:2)+NH4	7660.03391	(5459.78, 9860.29)	25655.68	(19595.13, 31716.23)	2.9071E-05	9.9558E-05	3.34929066
TAG(46:1/FA16:0)+NH4	9223.59322	(6618.15, 11829.03)	23864.6012	(19165.4, 28563.8)	2.9817E-05	0.00010142	2.5873432
TAG(54:5/FA20:2)+NH4	9174.40139	(6434.67, 11914.13)	24284.8965	(19234.75, 29335.04)	3.188E-05	0.00010743	2.64702791

TAG(52:4/FA20:0)+NH4	6951.11083	(4944.52, 8957.7)	19731.3484	(15513.48, 23949.22)	3.206E-05	0.00010743	2.83858924
TAG(52:8/FA18:2)+NH4	6360.59749	(5033.18, 7688.01)	17678.2735	(13995.85, 21360.69)	3.223E-05	0.00010743	2.77934165
LCER(24:0)+H	15769.9508	(11683.97, 19855.94)	60652.7049	(45112.25, 76193.16)	3.2629E-05	0.00010804	3.84609347
TAG(49:3/FA16:1)+NH4	4372.18725	(3331.39, 5412.99)	22320.3942	(15967.92, 28672.87)	3.6603E-05	0.00012004	5.10508653
TAG(58:8/FA18:1)+NH4	12032.9766	(8070.76, 15995.19)	33603.721	(27181.52, 40025.92)	3.6733E-05	0.00012004	2.79263579
TAG(52:4/FA16:1)+NH4	8187.33916	(5106.93, 11267.75)	39790.9061	(29182.67, 50399.14)	4.1513E-05	0.00013478	4.86005347
TAG(51:4/FA16:1)+NH4	4942.68975	(3773.59, 6111.79)	40441.5685	(28218.33, 52664.8)	4.4475E-05	0.00014347	8.18209731
TAG(55:2/FA18:1)+NH4	10633.1052	(8001.1, 13265.11)	26074.2415	(20659.69, 31488.79)	4.7373E-05	0.00015021	2.45217564
TAG(50:2/FA16:0)+NH4	10532.4627	(6547.92, 14517.01)	32296.9175	(24357.03, 40236.81)	4.7666E-05	0.00015021	3.06641652
TAG(58:7/FA18:0)+NH4	12692.9003	(8456.08, 16929.72)	38823.5634	(30315.84, 47331.28)	4.7711E-05	0.00015021	3.05868341
TAG(52:3/FA16:0)+NH4	11525.2017	(6672.68, 16377.72)	44495.3461	(33932.9, 55057.79)	4.7889E-05	0.00015021	3.86069999
TAG(56:9/FA18:3)+NH4	11773.2916	(8737.35, 14809.23)	43761.2569	(32357.32, 55165.2)	4.8284E-05	0.00015021	3.71699422
TAG(56:4/FA20:3)+NH4	9881.59279	(5209.8, 14553.38)	26868.2934	(21718.35, 32018.24)	4.8366E-05	0.00015021	2.71902455
HCER(d18:0/26:1)+H	1355.81381	(930.25, 1781.38)	12101.0382	(7721.49, 16480.58)	4.9742E-05	0.00015352	8.92529503
TAG(48:2/FA14:0)+NH4	5754.65176	(4371.61, 7137.69)	16112.055	(12319.55, 19904.56)	5.3217E-05	0.00016324	2.79983145
TAG(56:7/FA18:3)+NH4	18264.4542	(13619.45, 22909.46)	44967.8871	(35454.45, 54481.32)	5.8752E-05	0.00017912	2.46204384
TAG(54:5/FA18:2)+NH4	8435.94618	(6239.9, 10631.99)	18196.595	(15155.32, 21237.87)	6.015E-05	0.00018227	2.15703071
TAG(53:4/FA18:2)+NH4	7512.25341	(5532.96, 9491.55)	22283.7435	(16815.55, 27751.94)	6.0763E-05	0.00018302	2.96631947
TAG(54:6/FA22:6)+NH4	8193.75151	(5469.79, 10917.71)	20172.8236	(16300.31, 24045.34)	6.1684E-05	0.00018468	2.46197649
TAG(58:2/FA18:1)+NH4	7131.03603	(5226.7, 9035.37)	17834.9201	(13876.26, 21793.58)	6.2651E-05	0.00018646	2.50102791
TAG(54:4/FA18:0)+NH4	7712.07927	(6102.63, 9321.53)	19970.0132	(15401.19, 24538.84)	6.4327E-05	0.00018783	2.58944605
TAG(56:6/FA22:5)+NH4	13059.2715	(8460.07, 17658.47)	43803.8652	(32503.89, 55103.84)	6.4496E-05	0.00018783	3.35423496
TAG(56:6/FA18:3)+NH4	11739.11	(9868.23, 13609.99)	36169.6111	(26782.79, 45556.43)	6.4545E-05	0.00018783	3.08112038
TAG(40:0/FA14:0)+NH4	5137.48073	(2612.63, 7662.33)	15774.3637	(12256.55, 19292.18)	6.4614E-05	0.00018783	3.07044727
TAG(56:8/FA18:2)+NH4	11245.6945	(9233.91, 13257.48)	42237.3142	(30774.05, 53700.58)	6.5871E-05	0.00019001	3.75586533

TAG(54:5/FA16:1)+NH4	15413.8998	(10904.06, 19923.74)	40414.6175	(31470.95, 49358.28)	6.6122E-05	0.00019001	2.62195927
TAG(52:7/FA20:5)+NH4	8566.4948	(5504.51, 11628.48)	21191.4976	(17014.54, 25368.46)	7.0133E-05	0.00020038	2.4737653
TAG(44:1/FA14:0)+NH4	3653.52511	(2184.49, 5122.56)	9867.74045	(7849.56, 11885.93)	7.2855E-05	0.00020697	2.70088206
PS(18:2/16:1)-H	1080205.79	(840209.1, 1320202.47)	3014856.47	(2309961.55, 3719751.39)	7.5988E-05	0.00021405	2.79100196
TAG(54:8/FA18:3)+NH4	8493.44407	(5657.72, 11329.17)	18345.7606	(15287.54, 21403.98)	7.6203E-05	0.00021405	2.15999074
TAG(51:4/FA18:2)+NH4	7334.41949	(4377.52, 10291.32)	19272.7629	(15276.87, 23268.66)	7.7383E-05	0.00021585	2.62771483
TAG(56:4/FA18:0)+NH4	12726.2876	(8447.03, 17005.55)	41790.8503	(31344.74, 52236.96)	7.7704E-05	0.00021585	3.28382098
TAG(50:1/FA18:1)+NH4	12315.9593	(6433.65, 18198.27)	43800.7316	(34759.39, 52842.07)	8.1212E-05	0.00022434	3.5564206
TAG(58:9/FA22:6)+NH4	9411.00066	(6089.08, 12732.92)	19505.6555	(16714.98, 22296.33)	8.4039E-05	0.00023088	2.07264415
PE(P-18:2/20:4)-H	2709126.14	(2123814.58, 3294437.7)	4824055.78	(4153112.89, 5494998.67)	8.4633E-05	0.00023124	1.78066857
TAG(52:2/FA18:1)+NH4	27233.9179	(13106.43, 41361.41)	78919.284	(65784.53, 92054.03)	8.8704E-05	0.00024104	2.89783072
TAG(46:3/FA18:3)+NH4	18023.7032	(14100.5, 21946.9)	33267.4931	(28180.28, 38354.71)	9.4655E-05	0.00025539	1.84576348
TAG(58:7/FA22:6)+NH4	14043.2085	(10054.57, 18031.85)	35973.813	(28494.91, 43452.71)	9.5006E-05	0.00025539	2.561652
TAG(56:7/FA22:6)+NH4	10852.13	(6253.01, 15451.25)	36783.1639	(27326.82, 46239.51)	9.5924E-05	0.00025648	3.38948796
TAG(52:6/FA18:2)+NH4	7807.8504	(5681.58, 9934.12)	18267.7799	(14482.85, 22052.71)	9.7115E-05	0.00025768	2.33966828
TAG(50:3/FA16:1)+NH4	6921.87744	(5069.5, 8774.26)	23613.5805	(16917.2, 30309.96)	9.7976E-05	0.00025768	3.41144157
TAG(56:9/FA20:4)+NH4	6743.56865	(4346.98, 9140.16)	16479.8221	(13058.13, 19901.52)	9.8219E-05	0.00025768	2.44378354
TAG(47:1/FA17:0)+NH4	12017.1994	(9804.41, 14229.99)	24375.9128	(20136.9, 28614.93)	9.8435E-05	0.00025768	2.02841876
TAG(52:5/FA20:4)+NH4	6150.68586	(4416.32, 7885.05)	27769.4714	(17769.67, 37769.27)	9.9896E-05	0.00026015	4.5148577
TAG(53:4/FA16:0)+NH4	13087.9538	(10217.32, 15958.59)	35966.4484	(26708.06, 45224.84)	0.00010178	0.00026277	2.7480574
TAG(53:0/FA16:0)+NH4	8623.41412	(6279.71, 10967.12)	22449.6197	(17339.73, 27559.51)	0.00010195	0.00026277	2.60333314
TAG(53:1/FA17:0)+NH4	9198.158	(5359.13, 13037.19)	34559.5922	(24471.55, 44647.64)	0.00010442	0.00026659	3.75722967
TAG(48:4/FA16:1)+NH4	7844.63477	(5372.45, 10316.82)	19719.2901	(15269.73, 24168.85)	0.00010464	0.00026659	2.51372953
TAG(52:4/FA18:2)+NH4	8990.7591	(5906.83, 12074.69)	19711.9759	(16294.58, 23129.37)	0.00010504	0.00026659	2.1924707
TAG(58:7/FA22:4)+NH4	10357.8086	(6646.57, 14069.05)	25257.1021	(19996.35, 30517.86)	0.00010694	0.00027006	2.43846002

TAG(56:4/FA16:0)+NH4	10878.9143	(6870.14, 14887.69)	32838.6417	(24629.72, 41047.56)	0.00010767	0.00027052	3.01855873
TAG(49:1/FA16:1)+NH4	6029.4677	(5010.1, 7048.83)	17500.2352	(13107.94, 21892.53)	0.00011073	0.00027684	2.90245111
TAG(49:2/FA17:0)+NH4	6567.50364	(3279.05, 9855.96)	25325.1837	(18508.8, 32141.56)	0.00011196	0.00027691	3.85613547
TAG(50:4/FA18:1)+NH4	18946.1808	(13803.34, 24089.02)	35634.5898	(30515.95, 40753.23)	0.00011206	0.00027691	1.88083236
TAG(51:2/FA16:1)+NH4	5752.63052	(4455.05, 7050.21)	17376.8524	(12965.23, 21788.48)	0.00011242	0.00027691	3.02067938
TAG(58:6/FA20:4)+NH4	11328.4093	(8451.24, 14205.58)	58009.6078	(39859.0, 76160.22)	0.00011936	0.00029254	5.12071962
TAG(54:3/FA18:2)+NH4	11680.6004	(8774.44, 14586.76)	29908.8962	(23139.31, 36678.48)	0.00012311	0.00030026	2.56056155
TAG(46:1/FA14:0)+NH4	6224.32255	(4573.83, 7874.81)	13740.8774	(11007.02, 16474.74)	0.00012386	0.00030064	2.20761011
TAG(52:4/FA18:1)+NH4	7666.50376	(5686.03, 9646.98)	19905.2241	(15361.9, 24448.55)	0.00012496	0.0003018	2.59638874
TAG(58:7/FA20:4)+NH4	14795.0195	(11894.04, 17696.0)	33092.9715	(26002.3, 40183.65)	0.00012555	0.0003018	2.2367643
TAG(54:7/FA18:1)+NH4	7197.28829	(4950.85, 9443.72)	23845.7352	(17520.62, 30170.85)	0.00013161	0.00031486	3.31315549
TAG(56:7/FA16:1)+NH4	26783.9901	(17802.33, 35765.65)	77885.3223	(58264.7, 97505.95)	0.00013298	0.00031662	2.90790587
TAG(44:2/FA18:1)+NH4	13512.3753	(10667.29, 16357.47)	26176.2087	(21713.76, 30638.66)	0.00013569	0.00032154	1.93720261
TAG(56:3/FA20:1)+NH4	13652.9963	(9252.36, 18053.64)	36455.7184	(27635.47, 45275.96)	0.00013915	0.00032819	2.67016248
TAG(52:6/FA16:0)+NH4	6287.59851	(4914.61, 7660.58)	23572.818	(16761.65, 30383.99)	0.00014844	0.00034673	3.7490972
TAG(58:10/FA22:6)+NH4	10131.5875	(7700.98, 12562.2)	24307.0943	(19218.86, 29395.33)	0.00014886	0.00034673	2.39913976
CE(20:1)+H	11885.3292	(8513.5, 15257.16)	22477.1218	(19236.32, 25717.93)	0.00014909	0.00034673	1.89116528
TAG(54:6/FA20:3)+NH4	6116.88631	(4233.28, 8000.5)	13997.1561	(11191.49, 16802.82)	0.00015351	0.00035443	2.28828122
TAG(50:2/FA18:2)+NH4	6973.59211	(4397.04, 9550.15)	17239.9399	(13577.05, 20902.83)	0.00015382	0.00035443	2.47217497
TAG(44:0/FA16:0)+NH4	6937.46839	(5422.61, 8452.32)	16562.4455	(13048.97, 20075.93)	0.00016037	0.00036782	2.38739041
TAG(54:8/FA18:2)+NH4	6321.26968	(4994.78, 7647.76)	15104.8271	(11590.54, 18619.11)	0.00016624	0.00037955	2.38952424
TAG(53:3/FA16:0)+NH4	7749.16584	(6156.15, 9342.18)	32479.6981	(22041.4, 42917.99)	0.00018899	0.00042951	4.19137992
TAG(51:0/FA17:0)+NH4	6259.33357	(4542.23, 7976.44)	16365.8974	(12452.79, 20279.0)	0.00020642	0.00046511	2.61463896
TAG(50:2/FA14:0)+NH4	6776.83021	(3794.94, 9758.72)	17913.5607	(14152.32, 21674.8)	0.00020651	0.00046511	2.64335392
TAG(54:3/FA18:3)+NH4	7906.61248	(5317.52, 10495.71)	18193.0941	(14396.4, 21989.79)	0.00020768	0.00046564	2.30099732

TAG(57:3/FA18:2)+NH4	5690.94315	(3980.77, 7401.12)	13606.9293	(10489.31, 16724.55)	0.00021215	0.00047354	2.39097966
TAG(53:3/FA17:0)+NH4	6292.54949	(4399.58, 8185.52)	33356.5375	(22535.93, 44177.14)	0.0002138	0.00047475	5.30095751
TAG(51:0/FA18:0)+NH4	6809.68822	(5095.2, 8524.18)	16139.8615	(12699.22, 19580.51)	0.00021476	0.00047475	2.37013223
TAG(53:1/FA16:0)+NH4	7415.5691	(5594.12, 9237.02)	18610.3416	(14418.51, 22802.18)	0.00021641	0.00047475	2.50963093
TAG(51:2/FA18:2)+NH4	6559.24907	(5214.48, 7904.02)	15942.1251	(12073.11, 19811.14)	0.00021649	0.00047475	2.43048022
TAG(54:7/FA20:4)+NH4	7095.99522	(5348.47, 8843.52)	17448.2462	(13248.5, 21647.99)	0.00022041	0.00048124	2.45888642
TAG(52:4/FA18:0)+NH4	7091.43331	(5105.76, 9077.11)	18090.9507	(13836.81, 22345.09)	0.00022143	0.00048138	2.55109932
TAG(52:2/FA20:1)+NH4	7992.02429	(5727.35, 10256.7)	18871.8475	(14572.41, 23171.29)	0.00024025	0.00052002	2.36133511
TAG(54:7/FA18:3)+NH4	8508.27414	(6186.35, 10830.19)	20767.4685	(15825.43, 25709.51)	0.00024532	0.00052871	2.44085559
TAG(54:4/FA18:2)+NH4	13532.8851	(9588.2, 17477.57)	43781.677	(32070.57, 55492.79)	0.00025389	0.00054482	3.23520644
TAG(48:1/FA18:1)+NH4	9051.75794	(4412.92, 13690.6)	26472.1378	(19975.89, 32968.39)	0.00026898	0.00057442	2.92453001
TAG(44:1/FA16:0)+NH4	5470.06553	(3334.84, 7605.29)	16498.6241	(12366.14, 20631.11)	0.00026998	0.00057442	3.01616571
TAG(48:0/FA14:0)+NH4	7495.57628	(5025.79, 9965.36)	16520.7731	(13091.88, 19949.67)	0.00027463	0.00058184	2.20406977
LCER(22:1)+H	24958.8033	(17888.62, 32028.98)	54191.2105	(42862.23, 65520.19)	0.0002827	0.00059642	2.17122632
TAG(56:4/FA18:1)+NH4	14504.2419	(10646.5, 18361.98)	28304.9815	(23344.56, 33265.4)	0.00029725	0.00062395	1.95149679
TAG(56:6/FA18:0)+NH4	12160.2393	(7604.6, 16715.88)	40242.7184	(29384.87, 51100.57)	0.00029825	0.00062395	3.30936894
TAG(52:4/FA16:0)+NH4	8078.51159	(5958.27, 10198.76)	26601.0408	(18588.15, 34613.93)	0.00030378	0.00063288	3.29281459
TAG(48:4/FA14:0)+NH4	4983.58382	(3765.51, 6201.65)	11519.1538	(8805.0, 14233.31)	0.00031881	0.00066143	2.3114197
TAG(52:3/FA18:3)+NH4	8256.77414	(5548.57, 10964.97)	18132.1584	(14398.08, 21866.23)	0.00032956	0.0006809	2.1960342
TAG(44:1/FA18:1)+NH4	13333.0999	(8571.81, 18094.39)	31142.8735	(24050.65, 38235.09)	0.00033315	0.00068549	2.3357564
TAG(52:1/FA20:0)+NH4	9022.2688	(6015.16, 12029.37)	18539.4261	(15169.58, 21909.27)	0.00033941	0.00069552	2.05485189
TAG(58:8/FA18:2)+NH4	26076.8164	(18531.43, 33622.2)	113181.185	(74660.74, 151701.63)	0.00034108	0.00069608	4.34029917
TAG(54:3/FA16:0)+NH4	10968.8889	(7990.45, 13947.33)	30661.141	(22202.35, 39119.93)	0.00034669	0.00070465	2.79528229
TAG(52:5/FA16:0)+NH4	6758.69121	(4676.93, 8840.46)	25889.4514	(18152.01, 33626.9)	0.00036377	0.00073637	3.83054213
TAG(56:5/FA22:5)+NH4	7459.778	(4948.03, 9971.52)	17613.8633	(13449.23, 21778.49)	0.00037314	0.0007523	2.36117794

TAG(51:3/FA16:1)+NH4	8093.30352	(4573.76, 11612.85)	25108.0481	(18285.29, 31930.81)	0.00038357	0.00077021	3.10232379
TAG(56:4/FA18:2)+NH4	9074.02872	(6357.56, 11790.5)	21323.9439	(16112.19, 26535.7)	0.00039456	0.00078911	2.3499974
TAG(56:6/FA18:1)+NH4	12326.5129	(7457.81, 17195.22)	27768.7847	(22153.47, 33384.1)	0.00040381	0.0008044	2.25276889
TAG(55:2/FA18:2)+NH4	6917.2734	(5086.74, 8747.81)	15309.1508	(11949.43, 18668.87)	0.00041279	0.00081902	2.21317706
TAG(52:5/FA18:1)+NH4	11449.0636	(7973.52, 14924.61)	22027.5618	(18229.97, 25825.15)	0.00043746	0.00086454	1.92396187
TAG(56:7/FA22:4)+NH4	8497.40941	(6570.94, 10423.88)	15812.7534	(12993.22, 18632.29)	0.00044133	0.00086875	1.86089108
TAG(51:4/FA18:3)+NH4	6518.58457	(4674.53, 8362.64)	14629.9645	(11505.68, 17754.25)	0.00044632	0.00087513	2.24434681
TAG(52:4/FA20:3)+NH4	8617.32984	(5449.77, 11784.89)	29645.7651	(20371.07, 38920.46)	0.00044868	0.00087633	3.44024955
TAG(52:6/FA18:3)+NH4	8334.95082	(6353.63, 10316.27)	19122.9037	(14633.86, 23611.94)	0.000459	0.00089299	2.29430312
TAG(54:4/FA16:1)+NH4	11051.6766	(6914.02, 15189.33)	29843.5922	(22535.27, 37151.92)	0.00046143	0.00089425	2.70036786
TAG(52:5/FA20:3)+NH4	8810.29613	(5367.13, 12253.46)	26884.6899	(20013.16, 33756.22)	0.00046699	0.00090152	3.05150808
LCER(24:1)+H	33610.5045	(25204.43, 42016.57)	115246.227	(82954.35, 147538.1)	0.00050047	0.00096244	3.42887527
TAG(54:4/FA20:1)+NH4	11086.0995	(8244.84, 13927.36)	25467.6653	(19465.17, 31470.16)	0.00051711	0.00099063	2.29726113
TAG(48:1/FA16:1)+NH4	9572.24331	(6825.63, 12318.86)	20754.673	(16100.32, 25409.03)	0.00053583	0.00102258	2.16821411
CE(20:4)+H	45307.5727	(32282.71, 58332.44)	120421.987	(88416.82, 152427.16)	0.00053789	0.00102261	2.65787771
TAG(54:6/FA16:1)+NH4	10494.1909	(7325.95, 13662.43)	23390.7023	(18374.61, 28406.79)	0.00055643	0.00105384	2.22891907
HCER(14:0)+H	258565.679	(-15866.1, 532997.45)	3708712.36	(2513701.02, 4903723.71)	0.00056431	0.00106474	14.3434054
TAG(48:4/FA20:4)+NH4	10100.8498	(7336.23, 12865.47)	25155.2904	(18481.1, 31829.48)	0.00057689	0.00108437	2.49041328
DAG(18:1/22:4)+NH4	29668.1964	(24039.83, 35296.56)	65653.851	(49384.59, 81923.11)	0.00058807	0.00110126	2.21293705
TAG(52:5/FA20:5)+NH4	6978.78684	(5271.37, 8686.21)	16475.1129	(12297.61, 20652.61)	0.00060304	0.00112507	2.36074166
TAG(52:3/FA20:1)+NH4	7182.8508	(5750.77, 8614.94)	19562.9565	(14058.73, 25067.19)	0.00061444	0.00114208	2.72356436
TAG(44:2/FA14:0)+NH4	5758.70121	(4335.26, 7182.14)	12080.2565	(9334.2, 14826.31)	0.00064719	0.0011985	2.09773976
TAG(56:3/FA16:0)+NH4	19602.7454	(16589.12, 22616.38)	32721.556	(27219.55, 38223.56)	0.00067442	0.00124432	1.66923333
TAG(54:5/FA16:0)+NH4	12189.872	(8272.04, 16107.71)	32464.1176	(23149.96, 41778.27)	0.00068692	0.00126272	2.66320413
TAG(46:3/FA18:2)+NH4	22471.2224	(15579.46, 29362.98)	53199.8974	(38507.3, 67892.49)	0.00071358	0.00130693	2.36746789

TAG(52:7/FA16:0)+NH4	10238.8105	(7190.91, 13286.71)	24084.521	(18187.17, 29981.88)	0.00071945	0.00131286	2.35227726
TAG(56:2/FA16:0)+NH4	8697.07535	(6681.97, 10712.18)	13424.6981	(11963.75, 14885.65)	0.00074357	0.00135194	1.54358764
TAG(53:2/FA18:2)+NH4	8230.02015	(5472.03, 10988.01)	16614.4404	(13347.52, 19881.36)	0.00075015	0.00135897	2.0187606
TAG(53:5/FA20:4)+NH4	7920.67699	(5782.76, 10058.59)	16890.5992	(13088.42, 20692.78)	0.00078355	0.00141434	2.13246913
TAG(52:1/FA18:0)+NH4	16537.7164	(9754.47, 23320.96)	36902.7994	(28958.31, 44847.28)	0.00079007	0.00142099	2.23143259
TAG(47:0/FA17:0)+NH4	7186.84458	(5320.86, 9052.83)	17550.7539	(12960.43, 22141.08)	0.00079516	0.00142501	2.44206671
TAG(44:0/FA18:0)+NH4	7008.40834	(3277.38, 10739.44)	21286.0315	(15017.27, 27554.79)	0.00081559	0.00145641	3.03721337
PE(18:2/20:5)-H	23703.8036	(17537.85, 29869.75)	41010.6317	(34387.4, 47633.86)	0.00082106	0.00146096	1.73012874
TAG(51:5/FA18:3)+NH4	7920.24784	(5794.22, 10046.27)	21282.4901	(15026.68, 27538.3)	0.00082504	0.00146283	2.687099
TAG(51:5/FA18:2)+NH4	7117.53734	(5732.13, 8502.94)	20025.7374	(14074.64, 25976.84)	0.0008468	0.00149612	2.81357673
TAG(50:0/FA16:0)+NH4	12557.6241	(6041.31, 19073.94)	30975.9792	(24260.18, 37691.78)	0.0008702	0.00153204	2.466707
LCER(d18:0/20:0)+H	158869.048	(114872.78, 202865.32)	77718.9465	(63573.42, 91864.48)	0.00087362	0.00153267	-2.04414824
DAG(18:0/18:2)+NH4	461680.28	(326805.44, 596555.12)	799671.774	(681690.9, 917652.65)	0.00089961	0.00156918	1.73208995
TAG(46:0/FA18:0)+NH4	7513.33278	(5046.34, 9980.32)	19556.7035	(13898.22, 25215.18)	0.00090071	0.00156918	2.60293323
TAG(54:3/FA16:1)+NH4	9334.20525	(7106.92, 11561.49)	20235.9901	(15224.09, 25247.89)	0.00090697	0.0015746	2.16793926
TAG(50:1/FA16:1)+NH4	7252.86171	(5215.79, 9289.93)	15399.9631	(11792.5, 19007.43)	0.00091553	0.00158397	2.12329474
CE(20:2)+H	16918.2635	(13603.35, 20233.18)	29334.9881	(23924.17, 34745.8)	0.00092421	0.00159346	1.7339243
TAG(56:8/FA18:1)+NH4	9613.62404	(7217.91, 12009.34)	25772.0251	(18365.98, 33178.07)	0.00093115	0.00159991	2.68078146
DAG(18:2/22:6)+NH4	14774.4944	(11620.95, 17928.04)	26824.7004	(21708.61, 31940.79)	0.00093937	0.00160851	1.81560869
TAG(54:4/FA18:3)+NH4	18729.9665	(12901.75, 24558.18)	43429.731	(32302.24, 54557.22)	0.00094733	0.0016166	2.31872977
TAG(55:3/FA18:1)+NH4	12039.1679	(8131.36, 15946.97)	24132.5606	(19194.73, 29070.39)	0.00096087	0.00163414	2.00450402
TAG(40:0/FA16:0)+NH4	8650.85776	(6635.13, 10666.59)	17657.663	(13544.12, 21771.21)	0.00097371	0.00165036	2.04114592
TAG(50:3/FA16:0)+NH4	7195.05224	(4885.94, 9504.17)	18586.9433	(13290.5, 23883.39)	0.00098811	0.0016691	2.58329512
CE(22:1)+H	10705.7036	(6945.64, 14465.77)	24953.5457	(18659.38, 31247.71)	0.00100076	0.00168464	2.33086462

TAG(58:8/FA22:6)+NH4	21978.6548	(14582.49, 29374.82)	56408.33	(41699.66, 71117.0)	0.00100404	0.00168464	2.56650511
TAG(46:3/FA16:0)+NH4	8406.63192	(5643.42, 11169.84)	20928.0818	(15377.49, 26478.67)	0.00100957	0.00168824	2.48947284
TAG(55:4/FA18:1)+NH4	13778.0119	(10544.65, 17011.37)	23709.7763	(19634.3, 27785.25)	0.00101626	0.00169377	1.72084161
TAG(48:3/FA14:0)+NH4	7058.77287	(4879.33, 9238.21)	15300.5066	(11581.27, 19019.74)	0.00102788	0.00170744	2.16758733
CER(14:0)+H	60214.0923	(46724.29, 73703.89)	102510.787	(84792.74, 120228.83)	0.00109416	0.00181152	1.70243847
TAG(49:2/FA14:0)+NH4	5544.43284	(4390.04, 6698.82)	14428.4832	(10073.48, 18783.48)	0.00110437	0.00182239	2.60233708
TAG(47:2/FA18:2)+NH4	11872.7932	(8176.57, 15569.02)	29682.5235	(21933.83, 37431.22)	0.001126	0.00185197	2.50004553
TAG(42:2/FA18:2)+NH4	8424.00504	(5666.38, 11181.63)	18344.4589	(13950.01, 22738.91)	0.00114045	0.00186959	2.17764102
TAG(56:7/FA20:3)+NH4	8948.5886	(6330.92, 11566.26)	19844.9345	(15190.48, 24499.39)	0.00115653	0.00188976	2.21766084
TAG(56:5/FA20:3)+NH4	13514.7503	(9831.37, 17198.13)	31737.9188	(23097.95, 40377.89)	0.00116088	0.00189068	2.34839106
TAG(49:3/FA16:0)+NH4	7079.8202	(4923.11, 9236.53)	18109.4144	(13037.08, 23181.74)	0.00119351	0.00193752	2.55789186
PI(20:0/20:4)-H	175516.065	(102793.51, 248238.62)	326505.186	(282587.22, 370423.16)	0.00126613	0.00204876	1.86025813
TAG(56:5/FA16:0)+NH4	15530.1463	(9329.37, 21730.92)	54553.8738	(36105.7, 73002.05)	0.00127967	0.00206399	3.51277268
TAG(56:6/FA18:2)+NH4	12126.7081	(8418.22, 15835.19)	56756.3871	(35087.85, 78424.92)	0.00128761	0.00207012	4.68027981
TAG(54:0/FA18:0)+NH4	11196.7873	(9025.72, 13367.85)	21737.7667	(16857.42, 26618.11)	0.00133082	0.00213273	1.94142892
TAG(45:0/FA14:0)+NH4	4674.3344	(3147.46, 6201.21)	14753.5599	(9935.21, 19571.91)	0.00136974	0.00218809	3.15629107
TAG(54:4/FA22:4)+NH4	7723.75697	(5680.89, 9766.62)	14809.804	(11521.8, 18097.8)	0.00140027	0.00222973	1.91743527
TAG(56:5/FA20:1)+NH4	10133.2036	(7468.02, 12798.39)	22145.4837	(16390.69, 27900.27)	0.00141564	0.00224705	2.18543756
TAG(54:2/FA18:1)+NH4	17357.7415	(7195.36, 27520.12)	48016.4621	(35574.9, 60458.02)	0.00145407	0.00230074	2.76628512
TAG(52:2/FA20:2)+NH4	11821.1244	(7097.05, 16545.2)	24257.0585	(19351.31, 29162.81)	0.00153023	0.00241361	2.05200941
TAG(52:5/FA18:2)+NH4	7276.14111	(4245.94, 10306.34)	21360.929	(14502.59, 28219.27)	0.00155093	0.00243857	2.93574969
PS(16:0/20:5)-H	5237820.01	(4683727.42, 5791912.6)	7283175.77	(6361401.24, 8204950.3)	0.00156926	0.00245966	1.39049753
TAG(46:1/FA18:0)+NH4	14086.9695	(10308.19, 17865.74)	24723.8335	(20229.73, 29217.94)	0.00160376	0.00250588	1.75508533
TAG(50:2/FA16:1)+NH4	6275.47733	(3561.96, 8989.0)	24069.2529	(15758.57, 32379.94)	0.00163136	0.00254107	3.83544575
TAG(54:5/FA22:5)+NH4	7957.26742	(5958.76, 9955.77)	14592.0198	(11573.55, 17610.49)	0.00164576	0.00255553	1.83379784

TAG(54:3/FA20:3)+NH4	8614.60867	(5586.54, 11642.68)	18420.2039	(14109.23, 22731.18)	0.00169911	0.00263021	2.13825197
TAG(51:1/FA16:0)+NH4	8598.15901	(4875.74, 12320.58)	20438.5706	(15175.65, 25701.49)	0.00171273	0.00264077	2.37708684
TAG(50:3/FA18:2)+NH4	5616.21739	(4111.79, 7120.64)	16848.3466	(11297.87, 22398.82)	0.0017165	0.00264077	2.9999456
TAG(50:3/FA14:0)+NH4	5015.13251	(3433.09, 6597.18)	10940.7364	(8428.93, 13452.54)	0.00174428	0.00267078	2.18154483
TAG(56:3/FA18:0)+NH4	13562.2376	(9536.34, 17588.14)	31065.3265	(22304.64, 39826.01)	0.00174669	0.00267078	2.2905753
TAG(56:7/FA22:5)+NH4	8715.74739	(7110.76, 10320.74)	18537.6709	(13727.29, 23348.05)	0.00175774	0.00267948	2.12691696
TAG(51:1/FA17:0)+NH4	9292.84768	(6534.42, 12051.27)	26069.765	(17363.49, 34776.04)	0.00182219	0.00276346	2.80535805
TAG(54:5/FA20:5)+NH4	6055.00518	(4737.97, 7372.04)	15082.0033	(10608.95, 19555.06)	0.0018241	0.00276346	2.49083244
TAG(54:6/FA18:1)+NH4	10243.9922	(7354.05, 13133.93)	20019.302	(15617.85, 24420.75)	0.00182941	0.00276346	1.95424808
TAG(54:4/FA20:4)+NH4	8893.40354	(6022.18, 11764.63)	18803.968	(14130.6, 23477.33)	0.00193684	0.00291693	2.11437251
TAG(56:6/FA22:6)+NH4	12005.2813	(9504.85, 14505.71)	22455.5018	(17669.47, 27241.53)	0.00197768	0.00296949	1.8704686
TAG(48:2/FA16:0)+NH4	8945.67409	(5938.64, 11952.71)	18238.4909	(14088.5, 22388.48)	0.00208817	0.003126	2.03880565
TAG(52:4/FA20:2)+NH4	7983.58365	(5605.29, 10361.87)	17010.798	(12575.97, 21445.62)	0.00218466	0.00326069	2.13072209
TAG(48:4/FA16:0)+NH4	23409.019	(18479.22, 28338.82)	38370.8266	(31675.06, 45066.59)	0.00220371	0.00327933	1.63914715
TAG(56:3/FA20:0)+NH4	16216.7234	(13040.58, 19392.87)	30795.74	(23641.27, 37950.21)	0.00223288	0.00331287	1.89901125
TAG(44:3/FA18:2)+NH4	10000.6835	(6800.07, 13201.3)	19221.1218	(15154.0, 23288.25)	0.0022761	0.00336701	1.92198081
TAG(56:8/FA16:1)+NH4	11310.1735	(6189.85, 16430.5)	21985.7105	(18232.89, 25738.53)	0.00228389	0.00336857	1.94388799
TAG(49:2/FA16:0)+NH4	10607.0226	(8182.34, 13031.71)	22540.6878	(16612.94, 28468.43)	0.00230049	0.00337889	2.1250721
TAG(46:1/FA16:1)+NH4	8086.88454	(5613.22, 10560.55)	16621.9331	(12769.16, 20474.7)	0.0023044	0.00337889	2.05541862
DCER(20:0)+H	231387.949	(185148.06, 277627.84)	351392.194	(299418.41, 403365.98)	0.0023897	0.00349371	1.51862789
TAG(46:3/FA16:1)+NH4	8212.63719	(5851.87, 10573.4)	21737.923	(14614.99, 28860.85)	0.00239944	0.00349379	2.64688705
CE(20:5)+H	15932.3001	(11866.16, 19998.44)	28887.8108	(22730.51, 35045.11)	0.00240373	0.00349379	1.8131601
CE(22:0)+H	7940.7927	(5894.66, 9986.93)	23675.9311	(15904.98, 31446.89)	0.00243327	0.00352648	2.98155763
TAG(56:5/FA20:4)+NH4	16427.1032	(10538.59, 22315.62)	30833.0442	(24901.83, 36764.25)	0.00246782	0.00356621	1.87696174
TAG(56:3/FA18:1)+NH4	20759.6108	(14842.05, 26677.17)	34089.9634	(28854.04, 39325.88)	0.00251264	0.00362051	1.64212921

TAG(46:3/FA14:0)+NH4	5840.72689	(4014.91, 7666.55)	15878.5674	(10433.52, 21323.62)	0.00253435	0.0036413	2.71859439
TAG(52:3/FA18:2)+NH4	12249.4872	(7855.18, 16643.8)	24341.8388	(19311.29, 29372.39)	0.00262027	0.00375397	1.98717206
TAG(45:0/FA16:0)+NH4	5648.49636	(3588.71, 7708.28)	15364.9844	(10643.92, 20086.04)	0.00275337	0.00393338	2.7201902
TAG(44:2/FA16:0)+NH4	9515.72559	(5237.12, 13794.33)	20733.1944	(15817.91, 25648.48)	0.00279806	0.00398584	2.17883484
TAG(56:6/FA22:4)+NH4	8850.48923	(6486.31, 11214.66)	19450.8078	(14087.48, 24814.14)	0.00287	0.00407671	2.1977099
TAG(50:4/FA18:2)+NH4	13133.2473	(9736.34, 16530.16)	26768.4547	(19929.15, 33607.76)	0.00302101	0.00427905	2.03822057
TAG(54:1/FA20:1)+NH4	10985.712	(6790.83, 15180.59)	20174.8607	(16492.23, 23857.5)	0.00307105	0.00433764	1.83646365
TAG(54:6/FA20:4)+NH4	9264.85132	(5007.46, 13522.24)	19687.7666	(15099.54, 24275.99)	0.00313743	0.00441892	2.12499542
TAG(54:2/FA20:2)+NH4	9479.05509	(6838.1, 12120.01)	16973.7363	(13401.17, 20546.3)	0.00318112	0.00446786	1.790657
TAG(48:1/FA16:0)+NH4	14635.4449	(11167.52, 18103.37)	30823.7215	(22239.78, 39407.66)	0.00325061	0.00454442	2.10610075
TAG(56:5/FA18:2)+NH4	12171.5614	(5381.74, 18961.38)	25479.2574	(20919.14, 30039.37)	0.00325381	0.00454442	2.09334338
TAG(54:7/FA20:5)+NH4	8652.26883	(5930.46, 11374.07)	19754.3264	(14073.19, 25435.47)	0.00341729	0.00475946	2.28313831
TAG(54:4/FA16:0)+NH4	18586.7793	(14208.49, 22965.06)	65227.2022	(39851.03, 90603.38)	0.00345522	0.00479892	3.50933322
TAG(51:2/FA18:1)+NH4	10947.2206	(6082.21, 15812.23)	27754.8117	(19675.88, 35833.74)	0.0035232	0.00487978	2.53532953
TAG(49:2/FA18:2)+NH4	4443.84049	(3211.79, 5675.89)	15629.8254	(9755.9, 21503.75)	0.00379696	0.00524442	3.51718866
TAG(46:2/FA14:0)+NH4	6370.07276	(3911.12, 8829.02)	13599.7386	(10090.81, 17108.67)	0.00388408	0.00534998	2.13494242
PE(14:0/16:1)-H	930272.585	(741643.48, 1118901.7)	1295004.29	(1160312.97, 1429695.61)	0.00395197	0.00542853	1.39206971
TAG(54:8/FA22:6)+NH4	8586.87951	(6928.66, 10245.1)	14066.533	(11308.32, 16824.74)	0.00398984	0.00546554	1.63814259
TAG(45:1/FA16:0)+NH4	7837.67169	(6242.47, 9432.88)	16315.9994	(11911.25, 20720.75)	0.00417544	0.00569428	2.0817406
TAG(48:5/FA18:3)+NH4	10460.6211	(7314.7, 13606.54)	19865.4626	(15220.72, 24510.21)	0.0041796	0.00569428	1.89907105
TAG(58:10/FA22:5)+NH4	8933.53635	(6505.68, 11361.39)	14703.6988	(12044.55, 17362.85)	0.00430236	0.0058456	1.64589903
TAG(42:0/FA16:0)+NH4	7475.41976	(4414.27, 10536.57)	15723.2719	(11748.62, 19697.92)	0.00444792	0.00602699	2.10332963
TAG(52:2/FA14:0)+NH4	7725.60078	(5902.58, 9548.62)	14591.4661	(10975.04, 18207.89)	0.0045281	0.00611906	1.88871604
TAG(54:3/FA20:1)+NH4	9436.56625	(6594.26, 12278.88)	21434.7808	(14724.42, 28145.14)	0.00473521	0.00638169	2.2714598
TAG(52:1/FA16:0)+NH4	21718.6127	(12583.58, 30853.65)	50202.2412	(37265.82, 63138.66)	0.00480235	0.00644158	2.31148471

TAG(48:0/FA16:0)+NH4	21181.6823	(8095.71, 34267.65)	46755.994	(36726.33, 56785.66)	0.00480542	0.00644158	2.20737868
TAG(50:4/FA18:3)+NH4	18992.3683	(12235.23, 25749.51)	32932.3246	(27056.15, 38808.5)	0.00492188	0.00658005	1.73397673
PE(P-18:1/18:2)-H	48226679.9	(40079781.62, 56373578.09)	68155523.7	(58728579.6, 77582467.85)	0.00503326	0.00671101	1.41323276
TAG(47:1/FA14:0)+NH4	8128.3284	(5999.74, 10256.91)	15768.2297	(11361.83, 20174.63)	0.00508562	0.00676279	1.93991051
TAG(54:1/FA18:1)+NH4	14473.2379	(9104.75, 19841.72)	27980.2307	(21488.76, 34471.71)	0.00510639	0.00677241	1.93323918
TAG(47:1/FA18:1)+NH4	19025.1764	(14952.23, 23098.13)	28255.8457	(23996.01, 32515.69)	0.00544954	0.00720839	1.4851818
TAG(56:8/FA22:5)+NH4	7029.56419	(5306.7, 8752.43)	11956.6859	(9434.59, 14478.78)	0.00556788	0.00734549	1.70091425
TAG(50:4/FA16:0)+NH4	12860.119	(8729.07, 16991.16)	21517.3514	(17658.48, 25376.23)	0.00559093	0.00735649	1.67318447
TAG(52:6/FA20:5)+NH4	7865.22151	(6669.59, 9060.86)	19301.6552	(12565.96, 26037.35)	0.00567774	0.0074511	2.45405107
TAG(53:4/FA18:3)+NH4	9750.5273	(5956.58, 13544.48)	20226.5914	(14901.37, 25551.81)	0.00569821	0.00745838	2.07441001
TAG(50:6/FA20:4)+NH4	10769.7524	(7647.05, 13892.45)	19855.3349	(15155.11, 24555.56)	0.00574524	0.00750032	1.84362036
TAG(54:4/FA20:3)+NH4	13077.1825	(10672.74, 15481.62)	27224.6115	(19146.18, 35303.04)	0.00577132	0.00751475	2.08184076
TAG(48:4/FA18:2)+NH4	12904.1996	(7952.47, 17855.93)	27711.5896	(19975.1, 35448.08)	0.00582266	0.0075619	2.14748613
TAG(56:7/FA16:0)+NH4	9474.30113	(7021.7, 11926.91)	40029.1605	(21463.14, 58595.18)	0.00623776	0.00807999	4.22502515
TAG(55:5/FA20:4)+NH4	9153.88127	(6439.69, 11868.08)	18200.9376	(13454.25, 22947.62)	0.00641784	0.00829178	1.98833009
TAG(50:0/FA18:0)+NH4	13522.2922	(7671.5, 19373.08)	27654.6139	(20233.23, 35076.0)	0.00665737	0.00857909	2.04511288
TAG(50:5/FA14:0)+NH4	6802.07974	(4960.64, 8643.52)	11222.7885	(9013.71, 13431.87)	0.00691242	0.00888486	1.64990546
CE(22:4)+H	31097.8381	(21183.88, 41011.8)	58927.9737	(44183.24, 73672.71)	0.00695929	0.00892216	1.89492188
TAG(46:2/FA16:1)+NH4	11161.9087	(7610.33, 14713.49)	24857.9857	(16526.23, 33189.74)	0.00714007	0.00913053	2.22703719
LCER(d18:0/24:1)+H	37312.933	(29769.0, 44856.87)	56159.1387	(46212.68, 66105.6)	0.00732018	0.00933696	1.50508508
TAG(58:9/FA18:1)+NH4	11111.4169	(5717.12, 16505.72)	20114.5356	(16670.07, 23559.0)	0.00773124	0.00983618	1.81025838
CE(20:3)+H	17990.7033	(13260.37, 22721.04)	31640.7136	(23914.39, 39367.04)	0.00807616	0.01024893	1.75872578
TAG(50:0/FA14:0)+NH4	7582.30945	(5422.91, 9741.71)	12855.9879	(10096.91, 15615.06)	0.00835103	0.01054539	1.69552404
CE(18:2)+H	38747.0712	(27475.37, 50018.78)	62665.697	(50776.5, 74554.9)	0.00835195	0.01054539	1.61730152
TAG(54:1/FA18:0)+NH4	16744.467	(7853.98, 25634.95)	34018.5148	(25789.21, 42247.82)	0.00922642	0.01162018	2.03162722

TAG(55:4/FA18:2)+NH4	11410.1839	(5429.3, 17391.07)	22557.1061	(17502.23, 27611.98)	0.00952283	0.01196336	1.97692748
TAG(53:2/FA16:0)+NH4	10019.2985	(4696.91, 15341.69)	21723.5878	(15585.31, 27861.86)	0.00997401	0.01249876	2.16817454
TAG(46:0/FA16:0)+NH4	7543.3546	(5597.16, 9489.55)	18993.8707	(12811.64, 25176.1)	0.01001459	0.01251824	2.51796073
TAG(44:2/FA16:1)+NH4	6860.74637	(4740.28, 8981.21)	13732.3087	(9704.41, 17760.21)	0.01013928	0.01263563	2.00157649
DAG(18:2/22:5)+NH4	10316.4767	(7687.08, 12945.88)	16996.2407	(13200.73, 20791.75)	0.01015904	0.01263563	1.64748501
TAG(54:3/FA20:2)+NH4	12465.0284	(8142.38, 16787.67)	26689.2907	(18304.52, 35074.06)	0.01030524	0.01278565	2.14113356
TAG(46:3/FA18:1)+NH4	18149.7213	(12593.36, 23706.08)	31325.2471	(23933.05, 38717.44)	0.01087743	0.01346216	1.72593543
TAG(47:2/FA14:0)+NH4	8277.50289	(6196.49, 10358.51)	15441.8243	(11244.95, 19638.7)	0.01184777	0.01462688	1.86551724
TAG(42:1/FA16:0)+NH4	9557.95363	(7034.19, 12081.71)	16463.1854	(12300.16, 20626.21)	0.01209655	0.01487398	1.72245923
PE(P-18:0/22:6)-H	313320586	(291480509.75, 335160661.32)	376246824	(339012406.33, 413481241.1)	0.01210742	0.01487398	1.20083659
PE(P-18:2/18:2)-H	350988.423	(282091.3, 419885.55)	533858.708	(423119.73, 644597.68)	0.01229245	0.01506427	1.52101515
TAG(53:1/FA18:0)+NH4	9628.87408	(5327.42, 13930.33)	18192.2807	(13851.29, 22533.27)	0.01244871	0.01521847	1.88934662
TAG(48:3/FA18:3)+NH4	12876.5613	(9518.54, 16234.59)	20854.6776	(16320.41, 25388.94)	0.01299183	0.0158437	1.61958438
TAG(44:1/FA16:1)+NH4	6302.54973	(3997.44, 8607.66)	12524.1246	(8842.66, 16205.59)	0.01310317	0.0159406	1.98715205
TAG(48:1/FA14:0)+NH4	11906.5114	(6748.77, 17064.25)	23928.8414	(17254.42, 30603.26)	0.01486969	0.0180122	2.00972734
PE(P-18:2/22:6)-H	563486.28	(495420.45, 631552.11)	761373.423	(639214.6, 883532.25)	0.01487808	0.0180122	1.35118361
TAG(54:7/FA18:2)+NH4	8842.97072	(3979.11, 13706.83)	17701.9654	(13158.58, 22245.35)	0.01497359	0.01808405	2.00181206
TAG(54:3/FA18:1)+NH4	34024.6671	(14339.01, 53710.32)	67707.6762	(51176.71, 84238.64)	0.01588274	0.01913583	1.98995852
TAG(58:10/FA18:2)+NH4	9233.30177	(5624.25, 12842.35)	15057.8518	(12282.77, 17832.94)	0.01674634	0.02012781	1.63081985
PG(18:2/16:1)-H	5531605.2	(2969868.13, 8093342.26)	2310085.49	(1675820.13, 2944350.84)	0.01687778	0.02023715	-2.39454567
TAG(56:7/FA18:0)+NH4	11879.942	(9223.13, 14536.75)	19800.0818	(14933.87, 24666.29)	0.01706892	0.02041737	1.66668169
TAG(51:0/FA16:0)+NH4	8373.23733	(5665.44, 11081.03)	20586.0959	(11779.67, 29392.52)	0.0178944	0.0213537	2.45855875
TAG(53:3/FA18:2)+NH4	8444.78834	(5945.66, 10943.91)	13709.4289	(10556.78, 16862.08)	0.01796346	0.02138507	1.62341888
TAG(42:0/FA14:0)+NH4	4756.98821	(2809.69, 6704.28)	11262.3814	(7208.63, 15316.13)	0.0183755	0.02177299	2.36754453

TAG(44:0/FA14:0)+NH4	6549.45476	(4411.61, 8687.3)	13649.3081	(8976.64, 18321.98)	0.01837641	0.02177299	2.08403731
TAG(57:10/FA22:6)+NH4	11187.641	(7909.9, 14465.38)	16402.1293	(13846.68, 18957.58)	0.01985327	0.02345886	1.46609364
TAG(54:8/FA20:5)+NH4	8981.41245	(2444.99, 15517.83)	19613.4982	(14230.76, 24996.23)	0.01989312	0.02345886	2.18378772
TAG(56:4/FA20:4)+NH4	10073.6579	(7543.82, 12603.49)	17855.3551	(12582.55, 23128.16)	0.02235578	0.02630091	1.77247978
TAG(42:1/FA14:0)+NH4	4660.05836	(3281.42, 6038.69)	7101.2134	(5708.65, 8493.78)	0.02292368	0.02690573	1.52384645
TAG(47:2/FA16:1)+NH4	10351.9416	(7630.34, 13073.54)	17971.3443	(12849.59, 23093.1)	0.0231635	0.02712354	1.73603609
TAG(50:4/FA14:0)+NH4	14231.3127	(10498.07, 17964.56)	23383.952	(17330.83, 29437.08)	0.02571176	0.03003711	1.64313388
TAG(50:5/FA16:1)+NH4	13337.6597	(9333.1, 17342.22)	23010.7217	(16394.97, 29626.47)	0.0261009	0.03042063	1.72524432
TAG(52:0/FA18:0)+NH4	13967.2587	(9042.08, 18892.44)	24005.3282	(17364.0, 30646.65)	0.02768428	0.03219103	1.71868572
TAG(46:0/FA14:0)+NH4	9949.6588	(6614.01, 13285.31)	17172.8367	(12182.53, 22163.14)	0.02788433	0.03234842	1.72597243
TAG(50:4/FA20:4)+NH4	13685.6449	(9696.84, 17674.45)	28212.3311	(17114.98, 39309.69)	0.03007116	0.03480458	2.06145426
TAG(52:2/FA16:0)+NH4	20508.9579	(9890.91, 31127.0)	66834.9549	(38755.1, 94914.81)	0.03231346	0.03731346	3.25881769
LCER(d18:0/18:0)+H	30731.7553	(24254.58, 37208.93)	42490.5786	(34722.16, 50259.0)	0.0331624	0.03820553	1.38262778
TAG(52:4/FA20:4)+NH4	8421.5557	(4950.97, 11892.14)	14447.1127	(10634.54, 18259.68)	0.03328643	0.03826027	1.71549215
TAG(56:5/FA20:2)+NH4	12822.5916	(10018.44, 15626.74)	17591.9856	(14646.96, 20537.01)	0.03424135	0.0392676	1.37195242
PE(O-18:0/20:2)-H	5140462.47	(3992318.74, 6288606.2)	3399536.06	(2312185.09, 4486887.02)	0.03982444	0.04556572	-1.51210706
CE(16:0)+H	28343.5402	(19685.25, 37001.83)	41310.2356	(33298.71, 49321.76)	0.03995314	0.04560861	1.45748327
TAG(52:3/FA20:2)+NH4	9818.7995	(6430.58, 13207.02)	14240.0365	(11792.37, 16687.7)	0.04313638	0.04913027	1.45028285
PS(18:2/20:5)-H	196566.088	(110522.08, 282610.1)	305348.974	(246782.72, 363915.23)	0.04558027	0.05179576	1.55341634
LCER(d18:0/26:1)+H	13599.3545	(9689.03, 17509.68)	24369.9958	(15547.79, 33192.2)	0.04644918	0.05266347	1.79199651
CE(22:5)+H	21782.4913	(15339.26, 28225.72)	31888.8802	(24984.82, 38792.94)	0.04698294	0.05314812	1.46396845
PS(14:0/20:3)-H	123047.967	(95005.55, 151090.39)	178059.849	(135059.8, 221059.9)	0.04741802	0.05351921	1.44707672
PE(18:2/22:5)-H	381041.786	(258653.67, 503429.9)	594520.999	(445992.26, 743049.73)	0.04758279	0.05358423	1.56025145
CE(22:2)+H	23508.0019	(18416.91, 28599.09)	32769.3044	(26004.26, 39534.35)	0.04909065	0.05515803	1.39396383

DAG(18:2/20:3)+NH4	22718.1208	(17590.06, 27846.18)	31606.3435	(24929.79, 38282.89)	0.05257786	0.05894379	1.39123934
TAG(44:2/FA18:2)+NH4	10897.9361	(7575.35, 14220.52)	16384.1474	(12298.03, 20470.26)	0.05477437	0.06126887	1.50341746
TAG(52:2/FA18:2)+NH4	10743.7846	(6164.81, 15322.76)	16967.6658	(12844.54, 21090.79)	0.05702265	0.06364135	1.57930063
TAG(50:5/FA18:3)+NH4	17109.5123	(11889.39, 22329.64)	27236.9606	(19266.07, 35207.85)	0.05819849	0.064809	1.59191917
CE(18:3)+H	48518.7439	(34496.88, 62540.61)	72457.6202	(53490.37, 91424.87)	0.06234358	0.06927065	1.49339439
TAG(45:1/FA18:1)+NH4	17271.4356	(12864.31, 21678.56)	23807.0226	(19011.07, 28602.98)	0.06445746	0.0714606	1.37840439
LPG(20:1)-H	3569732.66	(2861284.44, 4278180.89)	4477068.77	(3860256.79, 5093880.76)	0.06742137	0.07451521	1.25417481
TAG(50:5/FA20:5)+NH4	13864.0788	(9290.49, 18437.67)	21091.0476	(15394.2, 26787.9)	0.06751078	0.07451521	1.52127292
CE(18:1)+H	32974.2813	(21794.55, 44154.01)	50412.9517	(36293.69, 64532.21)	0.06886757	0.07584534	1.52885672
TAG(46:2/FA16:0)+NH4	9619.31784	(7090.82, 12147.81)	14660.7443	(10398.5, 18922.99)	0.07112836	0.07816303	1.52409397
PS(14:0/22:4)-H	1021840.99	(669065.42, 1374616.57)	1641477.81	(1091893.52, 2191062.09)	0.07346836	0.08055742	1.6063926
TAG(46:1/FA18:1)+NH4	26001.7992	(15269.15, 36734.45)	38522.6984	(30502.0, 46543.4)	0.07766849	0.08497647	1.48153972
PS(18:1/20:1)-H	140948.065	(121100.54, 160795.59)	191328.039	(137756.35, 244899.73)	0.0796024	0.08690218	1.35743644
TAG(54:6/FA18:2)+NH4	12323.9718	(7937.05, 16710.89)	19801.5084	(13078.88, 26524.14)	0.08777561	0.09561614	1.6067473
TAG(42:1/FA16:1)+NH4	7015.37695	(3255.91, 10774.84)	11293.3755	(8238.11, 14348.64)	0.09148947	0.09944508	1.60980309
LCER(d18:0/24:0)+H	59337.7484	(40815.89, 77859.6)	41830.2524	(31440.8, 52219.71)	0.11120857	0.12061667	-1.4185367
LPG(20:3)-H	4585535.7	(3924359.88, 5246711.52)	5629989.78	(4645851.6, 6614127.95)	0.11198212	0.12119277	1.22777144
TAG(54:5/FA22:4)+NH4	14666.1602	(10230.99, 19101.33)	19170.0817	(15931.6, 22408.56)	0.11331586	0.12237134	1.30709616
TAG(48:0/FA18:0)+NH4	11909.7817	(7956.03, 15863.53)	16548.983	(12760.99, 20336.97)	0.1138816	0.12271724	1.38952867
PS(18:0/20:5)-H	6731898.25	(5308959.13, 8154837.37)	5433059.46	(4622637.61, 6243481.32)	0.1197717	0.12839085	-1.23906213
TAG(52:1/FA18:1)+NH4	21395.598	(6442.58, 36348.62)	34315.8761	(26883.88, 41747.88)	0.1198603	0.12839085	1.60387553
TAG(49:0/FA16:0)+NH4	10362.7167	(5375.36, 15350.07)	15150.7022	(11841.24, 18460.16)	0.11991705	0.12839085	1.46203959
PE(18:2/20:4)-H	2885047.78	(1653719.05, 4116376.52)	3834739.78	(3273750.48, 4395729.08)	0.13334696	0.1424647	1.32917722
LPG(20:2)-H	2183955.72	(1886222.52, 2481688.92)	2679610.56	(2151878.18, 3207342.94)	0.14388043	0.15339065	1.22695279

PE(18:2/20:3)-H	1805360	(1121698.37, 2489021.64)	2458449.09	(1937863.17, 2979035.0)	0.15163444	0.16131323	1.36175006
LPG(22:5)-H	121430.45	(93032.7, 149828.2)	158276.267	(120253.35, 196299.18)	0.15539278	0.16496049	1.30343144
PS(18:1/20:2)-H	74384.7075	(65664.65, 83104.77)	61377.2904	(45851.22, 76903.36)	0.16414378	0.17388112	-1.21192557
TAG(46:2/FA18:2)+NH4	16179.6474	(10849.54, 21509.75)	21607.269	(16407.99, 26806.54)	0.16469528	0.17409649	1.33545982
TAG(46:2/FA18:1)+NH4	107056.123	(73037.19, 141075.06)	81589.1173	(63795.85, 99382.39)	0.18842386	0.19873622	-1.31213729
PE(O-16:0/18:0)-H	1464214.38	(785986.76, 2142442.01)	1931375.84	(1660836.17, 2201915.5)	0.18879941	0.19873622	1.31905263
PE(14:0/20:5)-H	12558.9558	(10490.85, 14627.06)	10735.195	(9007.88, 12462.51)	0.19903875	0.20907432	-1.16988614
PS(18:2/20:1)-H	242792.361	(204157.03, 281427.69)	293744.379	(227895.96, 359592.8)	0.20148657	0.21120185	1.20985841
LPG(22:4)-H	963474.022	(819582.75, 1107365.3)	1131151.14	(924961.3, 1337340.98)	0.22281071	0.2330656	1.17403387
PE(P-18:1/18:3)-H	1360655.19	(937313.24, 1783997.15)	966630.509	(522633.53, 1410627.49)	0.22610059	0.23601314	-1.40762699
TAG(50:5/FA20:4)+NH4	25826.1687	(18834.36, 32817.98)	32119.81	(24568.88, 39670.74)	0.24522529	0.25544301	1.24369241
TAG(48:2/FA18:2)+NH4	12474.2559	(7453.98, 17494.53)	15676.4619	(12543.63, 18809.29)	0.28039492	0.29147081	1.25670517
TAG(46:4/FA18:2)+NH4	15238.8276	(6085.88, 24391.78)	20120.9648	(16325.32, 23916.61)	0.29387328	0.3048478	1.32037485
TAG(42:1/FA18:1)+NH4	17392.0918	(11876.18, 22908.01)	22829.5299	(15187.38, 30471.68)	0.29823557	0.30873247	1.31263853
PG(18:1/22:5)-H	1477681.71	(665966.96, 2289396.46)	1070004.65	(891690.14, 1248319.16)	0.32993535	0.34084231	-1.38100494
TAG(50:5/FA16:0)+NH4	16582.4362	(11817.83, 21347.04)	19662.9235	(15404.91, 23920.93)	0.35562651	0.36662527	1.18576808
LPG(20:0)-H	876640.405	(618244.47, 1135036.35)	1066414.36	(774697.36, 1358131.36)	0.35955592	0.3699135	1.21647867
DAG(16:0/16:0)+NH4	146922.878	(48397.62, 245448.13)	192718.171	(152649.67, 232786.67)	0.36176648	0.37142349	1.31169614
LPG(18:3)-H	44651.1424	(29884.85, 59417.43)	53786.4712	(40153.53, 67419.41)	0.38013457	0.38948214	1.2045934
PG(18:2/18:3)-H	131860.231	(46992.91, 216727.55)	95643.0271	(76749.9, 114536.16)	0.39248204	0.40131087	-1.37867061
PS(16:0/20:2)-H	240750.755	(181215.79, 300285.72)	277308.444	(201239.25, 353377.64)	0.46134043	0.47075554	1.1518487

TAG(47:2/FA18:1)+NH4	41970.2611	(31390.04, 52550.48)	37003.0457	(28348.1, 45658.0)	0.47958595	0.48837673	-1.13423801
PE(14:0/14:0)-H	344481.662	(294930.86, 394032.46)	364529.566	(332939.96, 396119.17)	0.49419896	0.50223472	1.0581973
PE(14:0/20:1)-H	1433874.27	(1233449.24, 1634299.3)	1330967.98	(1102784.97, 1559150.99)	0.52246792	0.52988632	-1.07731688
PS(16:0/22:6)-H	656210.676	(515116.05, 797305.3)	593243.903	(456480.07, 730007.74)	0.53488996	0.5413866	-1.10613977
LPG(17:1)-Hstd.IS	464962.506	(336562.15, 593362.86)	497254.382	(397675.48, 596833.28)	0.69535702	0.70238082	1.0694505
LPG(22:6)-H	55577.5682	(42061.69, 69093.45)	58654.0447	(47028.62, 70279.47)	0.73625149	0.742189	1.05535465
PG(14:1/14:1)-H	14102.3969	(9421.17, 18783.62)	15259.8999	(10373.78, 20146.02)	0.74179512	0.74627276	1.08207846
PS(14:0/20:1)-H	656113.265	(386128.74, 926097.79)	621659.756	(548669.82, 694649.69)	0.7998057	0.80301777	-1.05542181
PE(O-18:0/18:0)-H	1821315.51	(1308285.34, 2334345.68)	1766046.87	(1552818.72, 1979275.02)	0.83964507	0.84132772	-1.03129511
DAG(16:0/18:1)+NH4	598900.199	(-61934.56, 1259734.96)	664343.517	(520201.55, 808485.49)	0.84153177	0.84153177	1.10927249

Table S5: The list of lipids showing statistically significant concentration change (p<0.05; q<0.05) when AD patients compared to cognitively healthy controls. Grp0, Control; Grp1, AD.

Metabolite name	Grp0_Mean	Grp0_Mean-95CI	Grp1_Mean	Grp1_Mean-95CI	Ttest P value	Q value	Fold Change
DAG(14:0/14:0)+NH4	283272.121	(163123.55, 403420.69)	1064956.99	(935410.11, 1194503.87)	1.1337E-09	5.6686E-07	3.75948395
TAG(58:10/FA20:5)+NH4	12003.5217	(9665.95, 14341.1)	51161.7831	(41871.01, 60452.55)	6.0863E-09	1.2584E-06	4.26223108
TAG(48:4/FA18:3)+NH4	7953.6526	(5689.56, 10217.74)	28750.2472	(24119.93, 33380.56)	7.9485E-09	1.2584E-06	3.61472253
TAG(49:0/FA17:0)+NH4	7643.2736	(6088.12, 9198.43)	21962.0582	(18726.48, 25197.63)	1.0067E-08	1.2584E-06	2.87338375
TAG(56:6/FA20:5)+NH4	18056.7117	(15480.51, 20632.91)	57974.7788	(48182.77, 67766.79)	1.276E-08	1.276E-06	3.21070524
TAG(52:4/FA22:4)+NH4	8000.23331	(6566.21, 9434.26)	22155.355	(18910.66, 25400.05)	1.9943E-08	1.4261E-06	2.76933861
TAG(51:2/FA17:0)+NH4	8960.90893	(6690.55, 11231.26)	29582.0114	(24501.06, 34662.97)	2.1422E-08	1.4261E-06	3.30122889
TAG(48:5/FA18:2)+NH4	9321.35187	(7110.17, 11532.53)	28745.2465	(24181.11, 33309.38)	2.2818E-08	1.4261E-06	3.08380661
TAG(51:3/FA17:0)+NH4	8474.39483	(6199.86, 10748.93)	24229.1248	(20732.24, 27726.01)	3.0107E-08	1.6726E-06	2.85909794
DCER(18:1)+H	23538.6348	(19261.49, 27815.78)	102054.953	(81240.43, 122869.48)	4.6191E-08	2.2534E-06	4.33563602
SM(26:0)+H	10063140.1	(8052971.76, 12073308.36)	34533553.5	(28721794.56, 40345312.35)	4.9575E-08	2.2534E-06	3.43168765
TAG(48:3/FA18:2)+NH4	9993.72588	(6781.07, 13206.38)	44081.0473	(35098.97, 53063.13)	8.7802E-08	3.6584E-06	4.41087216
TAG(54:6/FA16:0)+NH4	16570.4568	(11247.51, 21893.4)	51345.1984	(43076.6, 59613.8)	1.0693E-07	4.1127E-06	3.09859885
TAG(46:3/FA18:3)+NH4	12400.4033	(9066.06, 15734.74)	33267.4931	(28180.28, 38354.71)	1.8797E-07	6.4851E-06	2.68277509
TAG(49:2/FA18:1)+NH4	8171.24956	(5548.68, 10793.82)	34566.6262	(27318.05, 41815.2)	1.9455E-07	6.4851E-06	4.2302742
TAG(53:6/FA20:4)+NH4	10304.2375	(8029.13, 12579.34)	29147.87	(24112.34, 34183.4)	2.0974E-07	6.5544E-06	2.82872654
TAG(56:7/FA20:4)+NH4	12881.9188	(9505.43, 16258.41)	29795.3837	(26138.78, 33451.99)	2.2366E-07	6.5781E-06	2.31296161
DAG(16:1/18:3)+NH4	50941.4486	(34222.61, 67660.29)	125728.722	(110770.31, 140687.13)	3.1667E-07	8.7964E-06	2.4681026
DAG(14:0/18:2)+NH4	39747.1261	(27554.69, 51939.56)	104825.123	(89396.07, 120254.18)	3.612E-07	9.5052E-06	2.63730069
TAG(44:2/FA18:1)+NH4	9763.15804	(7510.15, 12016.17)	26176.2087	(21713.76, 30638.66)	4.1583E-07	1.0013E-05	2.68112106
TAG(49:1/FA16:0)+NH4	9139.39469	(6417.62, 11861.17)	28158.3997	(23026.96, 33289.84)	4.3662E-07	1.0013E-05	3.08099175

TAG(52:6/FA20:4)+NH4	8709.40341	(6983.67, 10435.13)	23069.5281	(19177.49, 26961.56)	4.5366E-07	1.0013E-05	2.64880693
TAG(58:8/FA20:4)+NH4	24621.8347	(18864.75, 30378.92)	65236.6502	(54207.15, 76266.15)	4.6058E-07	1.0013E-05	2.64954464
TAG(49:2/FA16:1)+NH4	8022.10351	(5838.8, 10205.41)	20225.1497	(17161.0, 23289.3)	5.1622E-07	1.0755E-05	2.52117786
TAG(58:5/FA18:1)+NH4	12210.7431	(8926.79, 15494.7)	32299.7676	(27025.55, 37573.99)	5.4512E-07	1.078E-05	2.64519262
TAG(58:6/FA22:4)+NH4	16599.6907	(12455.23, 20744.15)	43653.322	(36369.52, 50937.12)	5.6057E-07	1.078E-05	2.6297672
TAG(58:6/FA18:0)+NH4	20772.0483	(15153.74, 26390.36)	98931.1181	(74986.02, 122876.22)	7.375E-07	1.3445E-05	4.76270402
TAG(50:4/FA20:3)+NH4	21368.1357	(16104.97, 26631.3)	63241.5512	(51144.24, 75338.86)	7.5292E-07	1.3445E-05	2.95961951
TAG(48:2/FA18:0)+NH4	11502.1443	(8442.69, 14561.6)	29702.4663	(24816.74, 34588.19)	1.2327E-06	2.1254E-05	2.58234164
TAG(49:1/FA18:1)+NH4	7226.64268	(4877.52, 9575.77)	30646.8943	(23394.85, 37898.94)	1.3122E-06	2.187E-05	4.24082049
TAG(50:1/FA20:1)+NH4	7059.42311	(5435.14, 8683.71)	22142.4746	(17457.79, 26827.16)	1.5493E-06	2.47E-05	3.13658414
TAG(46:1/FA16:0)+NH4	8348.67974	(6342.38, 10354.98)	23864.6012	(19165.4, 28563.8)	1.5955E-06	2.47E-05	2.85848804
TAG(50:2/FA18:1)+NH4	8573.58323	(7158.05, 9989.12)	27009.1566	(21097.34, 32920.97)	1.6302E-06	2.47E-05	3.15027637
CER(14:0)+H	41243.5298	(31458.22, 51028.84)	102510.787	(84792.74, 120228.83)	1.6825E-06	2.4743E-05	2.48549985
TAG(56:3/FA16:0)+NH4	14531.9076	(12010.58, 17053.24)	32721.556	(27219.55, 38223.56)	1.8932E-06	2.7046E-05	2.25170411
TAG(58:8/FA20:3)+NH4	12278.0519	(10514.94, 14041.16)	25353.7469	(21354.64, 29352.85)	2.0407E-06	2.8344E-05	2.06496496
TAG(58:7/FA18:1)+NH4	27808.3296	(20785.27, 34831.39)	81613.7682	(64957.33, 98270.21)	2.2193E-06	2.9795E-05	2.9348677
TAG(52:3/FA16:1)+NH4	11728.3481	(8140.52, 15316.18)	49843.8383	(37260.5, 62427.17)	2.2644E-06	2.9795E-05	4.24986009
LCER(26:1)+H	10765.2169	(7292.86, 14237.58)	37307.3542	(29057.53, 45557.17)	2.3603E-06	3.026E-05	3.46554599
TAG(48:4/FA18:1)+NH4	13450.2887	(10643.13, 16257.45)	40459.8737	(31741.02, 49178.72)	2.5855E-06	3.167E-05	3.00810448
TAG(56:7/FA18:1)+NH4	16734.3942	(12786.73, 20682.05)	36341.7988	(30986.19, 41697.41)	2.6107E-06	3.167E-05	2.17168297
TAG(47:1/FA17:0)+NH4	10439.2569	(8330.47, 12548.05)	24375.9128	(20136.9, 28614.93)	2.6603E-06	3.167E-05	2.33502375
TAG(56:9/FA20:5)+NH4	11530.6384	(8305.97, 14755.31)	27200.4941	(23053.46, 31347.53)	2.816E-06	3.2745E-05	2.35897555
TAG(55:2/FA18:1)+NH4	9441.05306	(7680.93, 11201.18)	26074.2415	(20659.69, 31488.79)	3.0051E-06	3.4148E-05	2.76179376
TAG(56:4/FA18:1)+NH4	11751.8583	(8902.15, 14601.57)	28304.9815	(23344.56, 33265.4)	3.505E-06	3.8298E-05	2.40855367
TAG(56:8/FA20:5)+NH4	10857.1926	(8179.49, 13534.9)	23116.2503	(19831.23, 26401.27)	3.5234E-06	3.8298E-05	2.12911857

TAG(56:5/FA18:1)+NH4	18173.3781	(15105.03, 21241.73)	43317.9885	(35150.17, 51485.81)	3.7381E-06	3.9768E-05	2.38359585
TAG(47:1/FA18:1)+NH4	12666.4825	(9282.85, 16050.11)	28255.8457	(23996.01, 32515.69)	4.0886E-06	4.2395E-05	2.2307571
TAG(54:5/FA18:3)+NH4	17549.1654	(12213.35, 22884.98)	60923.6345	(46738.14, 75109.12)	4.1732E-06	4.2395E-05	3.47159726
HCER(d18:0/18:0)+H	2851.09315	(1441.74, 4260.45)	26362.7952	(18056.87, 34668.72)	4.2395E-06	4.2395E-05	9.24655698
TAG(56:2/FA20:0)+NH4	15449.8556	(10206.92, 20692.79)	54504.2199	(42214.75, 66793.69)	4.8598E-06	4.7357E-05	3.5278142
TAG(40:0/FA14:0)+NH4	4585.31718	(3065.54, 6105.09)	15774.3637	(12256.55, 19292.18)	4.9252E-06	4.7357E-05	3.4401903
TAG(54:8/FA18:3)+NH4	8768.75577	(7218.25, 10319.26)	18345.7606	(15287.54, 21403.98)	5.0751E-06	4.7878E-05	2.09217374
TAG(56:6/FA16:0)+NH4	19132.1202	(11833.66, 26430.58)	121577.136	(83507.87, 159646.4)	5.2877E-06	4.8961E-05	6.35460861
TAG(44:0/FA16:0)+NH4	6141.04091	(4880.24, 7401.84)	16562.4455	(13048.97, 20075.93)	6.146E-06	5.5207E-05	2.69700947
TAG(47:1/FA16:0)+NH4	6096.42373	(4230.83, 7962.02)	18859.2314	(14828.36, 22890.1)	6.1832E-06	5.5207E-05	3.09349091
DCER(16:0)+H	221823.811	(160953.49, 282694.13)	496542.592	(418723.9, 574361.29)	6.538E-06	5.7351E-05	2.23845488
TAG(50:3/FA18:3)+NH4	7822.67542	(6786.01, 8859.34)	16616.3252	(13733.08, 19499.57)	6.6849E-06	5.7628E-05	2.12412306
TAG(55:5/FA18:2)+NH4	11516.9152	(9279.63, 13754.2)	28003.7252	(22488.34, 33519.11)	6.9329E-06	5.8754E-05	2.43153003
TAG(48:4/FA16:0)+NH4	17230.7129	(13525.51, 20935.92)	38370.8266	(31675.06, 45066.59)	7.2277E-06	6.0231E-05	2.22688561
TAG(52:4/FA16:1)+NH4	9155.05527	(5759.24, 12550.87)	39790.9061	(29182.67, 50399.14)	7.7253E-06	6.3322E-05	4.34633161
TAG(51:2/FA16:0)+NH4	11244.2026	(7331.57, 15156.84)	32572.123	(26038.9, 39105.34)	8.3681E-06	6.6587E-05	2.89679261
LCER(24:0)+H	16605.6541	(12390.96, 20820.35)	60652.7049	(45112.25, 76193.16)	8.3899E-06	6.6587E-05	3.65253332
TAG(54:1/FA20:0)+NH4	17370.313	(13369.93, 21370.7)	43764.5139	(34935.63, 52593.4)	8.9899E-06	7.0234E-05	2.51950059
TAG(52:3/FA16:0)+NH4	15213.6444	(12142.23, 18285.06)	44495.3461	(33932.9, 55057.79)	9.1999E-06	7.0768E-05	2.92470002
TAG(58:7/FA22:5)+NH4	24582.8245	(18705.19, 30460.46)	70882.5426	(54828.08, 86937.0)	9.7633E-06	7.3964E-05	2.88341735
TAG(56:2/FA20:1)+NH4	12216.4723	(10221.77, 14211.18)	23605.8595	(19808.16, 27403.56)	1.1033E-05	8.1864E-05	1.93229755
TAG(52:6/FA18:1)+NH4	10892.9417	(8518.04, 13267.84)	25764.2095	(20927.03, 30601.38)	1.1133E-05	8.1864E-05	2.36522054
TAG(58:7/FA18:2)+NH4	16859.8087	(13089.77, 20629.85)	33156.8072	(28359.9, 37953.72)	1.1981E-05	8.6619E-05	1.96661823
TAG(54:8/FA20:4)+NH4	9554.78192	(7156.26, 11953.31)	21270.5066	(17594.13, 24946.89)	1.2127E-05	8.6619E-05	2.22616349
DAG(16:0/16:0)+NH4	70756.6561	(48548.18, 92965.13)	192718.171	(152649.67, 232786.67)	1.2586E-05	8.8637E-05	2.72367551

LCER(22:1)+H	20009.8718	(13887.41, 26132.34)	54191.2105	(42862.23, 65520.19)	1.3149E-05	9.1313E-05	2.70822378
PE(P-18:0/20:3)-H	55618784.2	(51357994.33, 59879574.16)	72933837.3	(67979933.83, 77887740.75)	1.3475E-05	9.1596E-05	1.31131664
CE(20:4)+H	32978.6172	(24802.86, 41154.37)	120421.987	(88416.82, 152427.16)	1.3683E-05	9.1596E-05	3.65151719
TAG(52:7/FA18:1)+NH4	12861.9138	(8814.13, 16909.7)	31529.6644	(25751.94, 37307.39)	1.3755E-05	9.1596E-05	2.45139757
TAG(54:6/FA22:6)+NH4	8362.34693	(6373.83, 10350.87)	20172.8236	(16300.31, 24045.34)	1.4088E-05	9.1596E-05	2.41233995
TAG(58:6/FA18:1)+NH4	16469.5249	(12010.19, 20928.86)	34262.7863	(29214.7, 39310.88)	1.4106E-05	9.1596E-05	2.08037491
TAG(54:4/FA18:3)+NH4	13005.1114	(9883.98, 16126.25)	43429.731	(32302.24, 54557.22)	1.4832E-05	9.4031E-05	3.33943553
TAG(50:2/FA14:0)+NH4	7470.52588	(6208.6, 8732.45)	17913.5607	(14152.32, 21674.8)	1.4857E-05	9.4031E-05	2.39789822
TAG(52:2/FA20:2)+NH4	10271.0469	(8179.88, 12362.22)	24257.0585	(19351.31, 29162.81)	1.5674E-05	9.7966E-05	2.36169289
TAG(42:2/FA18:2)+NH4	5233.42293	(3551.98, 6914.86)	18344.4589	(13950.01, 22738.91)	1.7048E-05	0.00010523	3.50525061
TAG(50:2/FA20:2)+NH4	9026.0373	(7018.53, 11033.54)	25655.68	(19595.13, 31716.23)	1.7305E-05	0.00010552	2.84240794
TAG(58:8/FA18:1)+NH4	14039.9394	(10097.44, 17982.44)	33603.721	(27181.52, 40025.92)	1.815E-05	0.00010934	2.39343775
TAG(48:2/FA16:1)+NH4	9745.18598	(7224.6, 12265.78)	24321.1239	(19293.82, 29348.43)	1.8587E-05	0.00011063	2.49570649
TAG(49:0/FA18:0)+NH4	9465.85274	(6718.64, 12213.07)	30161.7308	(22654.3, 37669.16)	1.8897E-05	0.00011116	3.18637229
TAG(44:1/FA16:0)+NH4	5122.5874	(4060.21, 6184.97)	16498.6241	(12366.14, 20631.11)	2.0653E-05	0.00011917	3.22075991
TAG(52:5/FA18:1)+NH4	10931.0925	(8883.94, 12978.25)	22027.5618	(18229.97, 25825.15)	2.0735E-05	0.00011917	2.01512904
TAG(56:8/FA18:2)+NH4	11759.9597	(8620.99, 14898.93)	42237.3142	(30774.05, 53700.58)	2.1646E-05	0.00012299	3.59162066
TAG(47:0/FA14:0)+NH4	6496.6155	(5100.12, 7893.11)	13566.0532	(11175.38, 15956.73)	2.2996E-05	0.00012919	2.08817241
HCER(d18:0/20:0)+H	27432.2665	(19638.08, 35226.45)	225288.39	(148370.33, 302206.45)	2.6545E-05	0.00014747	8.21253284
CE(20:0)+H	16462.7093	(10849.45, 22075.97)	38824.2999	(31935.15, 45713.45)	2.822E-05	0.00015478	2.35831777
HCER(18:1)+H	924946.228	(715296.68, 1134595.77)	3757975.52	(2651065.43, 4864885.61)	2.8482E-05	0.00015478	4.06291242
HCER(14:0)+H	699199.717	(422662.68, 975736.75)	3708712.36	(2513701.02, 4903723.71)	2.879E-05	0.00015478	5.30422464
TAG(55:4/FA18:1)+NH4	11980.233	(9676.72, 14283.74)	23709.7763	(19634.3, 27785.25)	2.996E-05	0.00015876	1.97907471
TAG(51:4/FA18:2)+NH4	8276.81998	(6590.73, 9962.91)	19272.7629	(15276.87, 23268.66)	3.0164E-05	0.00015876	2.32852266
TAG(46:3/FA18:1)+NH4	11716.7259	(9086.57, 14346.88)	31325.2471	(23933.05, 38717.44)	3.1044E-05	0.00016135	2.67354952

TAG(58:9/FA22:6)+NH4	10827.8347	(8754.21, 12901.46)	19505.6555	(16714.98, 22296.33)	3.1592E-05	0.00016135	1.8014364
TAG(54:5/FA18:1)+NH4	13749.3317	(11396.63, 16102.03)	28640.8059	(23157.57, 34124.04)	3.1624E-05	0.00016135	2.08306895
TAG(40:0/FA16:0)+NH4	5756.02468	(3948.15, 7563.9)	17657.663	(13544.12, 21771.21)	3.24E-05	0.00016245	3.06768369
TAG(47:2/FA18:2)+NH4	9801.78019	(7888.59, 11714.97)	29682.5235	(21933.83, 37431.22)	3.2491E-05	0.00016245	3.02827883
TAG(50:3/FA18:0)+NH4	10760.9153	(8308.46, 13213.37)	22925.7214	(18698.01, 27153.43)	3.2842E-05	0.00016258	2.13046201
TAG(58:10/FA20:4)+NH4	15413.6733	(11340.46, 19486.89)	43395.6764	(33230.69, 53560.66)	3.4772E-05	0.00017045	2.81540134
TAG(58:7/FA18:0)+NH4	15289.0767	(11672.05, 18906.11)	38823.5634	(30315.84, 47331.28)	3.6119E-05	0.00017533	2.53930072
TAG(48:3/FA16:1)+NH4	9011.2463	(5813.83, 12208.66)	19813.2196	(16891.28, 22735.15)	3.687E-05	0.00017726	2.19872135
TAG(48:1/FA18:0)+NH4	10611.8621	(7186.71, 14037.01)	23986.3676	(19778.34, 28194.4)	3.7492E-05	0.00017853	2.26033541
TAG(58:7/FA16:0)+NH4	10315.9006	(8041.23, 12590.57)	20517.3233	(17240.34, 23794.3)	3.918E-05	0.00018481	1.98890276
DAG(16:1/20:2)+NH4	132841.833	(108402.77, 157280.89)	229661.887	(198665.97, 260657.8)	4.0082E-05	0.00018505	1.72883709
TAG(52:4/FA18:0)+NH4	7066.90962	(5617.4, 8516.42)	18090.9507	(13836.81, 22345.09)	4.0084E-05	0.00018505	2.55995218
TAG(56:3/FA18:1)+NH4	19001.6284	(15764.3, 22238.96)	34089.9634	(28854.04, 39325.88)	4.0497E-05	0.00018505	1.79405484
TAG(56:1/FA18:1)+NH4	12805.249	(9897.84, 15712.66)	31064.1271	(24202.28, 37925.97)	4.0712E-05	0.00018505	2.42589012
TAG(44:1/FA18:1)+NH4	12077.9205	(9043.4, 15112.44)	31142.8735	(24050.65, 38235.09)	4.2486E-05	0.00019138	2.57849632
CE(18:2)+H	27986.4426	(20186.74, 35786.14)	62665.697	(50776.5, 74554.9)	4.3307E-05	0.00019333	2.23914479
TAG(54:5/FA20:3)+NH4	9426.65048	(6619.56, 12233.74)	23214.8309	(18299.89, 28129.77)	4.3993E-05	0.00019409	2.46268077
TAG(50:1/FA14:0)+NH4	8168.92737	(5902.57, 10435.28)	18540.2649	(14932.76, 22147.77)	4.4395E-05	0.00019409	2.26960824
TAG(54:4/FA18:2)+NH4	14085.0387	(10652.48, 17517.59)	43781.677	(32070.57, 55492.79)	4.4641E-05	0.00019409	3.10838174
TAG(51:4/FA16:1)+NH4	10125.066	(7349.0, 12901.14)	40441.5685	(28218.33, 52664.8)	4.8428E-05	0.00020874	3.99420297
CE(22:2)+H	14484.5215	(11071.22, 17897.82)	32769.3044	(26004.26, 39534.35)	4.9938E-05	0.00021341	2.26236707
TAG(52:3/FA14:0)+NH4	8084.49038	(6096.59, 10072.39)	17949.1691	(14371.18, 21527.15)	5.0778E-05	0.00021516	2.22019796
TAG(54:6/FA20:5)+NH4	15264.7395	(11057.07, 19472.41)	44070.957	(32812.07, 55329.84)	5.468E-05	0.00022975	2.88710836
TAG(54:3/FA18:2)+NH4	12036.6266	(8858.79, 15214.46)	29908.8962	(23139.31, 36678.48)	5.6761E-05	0.00023495	2.4848238
TAG(52:3/FA20:0)+NH4	10079.6278	(8225.39, 11933.86)	22590.3814	(17691.58, 27489.18)	5.7199E-05	0.00023495	2.24119203

TAG(58:8/FA22:6)+NH4	19693.631	(15218.96, 24168.3)	56408.33	(41699.66, 71117.0)	5.7327E-05	0.00023495	2.86429303
TAG(45:1/FA18:1)+NH4	9416.99211	(6132.07, 12701.91)	23807.0226	(19011.07, 28602.98)	6.0612E-05	0.00024416	2.52809202
TAG(56:3/FA20:0)+NH4	12965.8724	(10711.27, 15220.47)	30795.74	(23641.27, 37950.21)	6.0966E-05	0.00024416	2.37513829
TAG(60:11/FA22:6)+NH4	12918.4288	(10112.48, 15724.38)	30085.0778	(23648.05, 36522.1)	6.1449E-05	0.00024416	2.32884961
TAG(48:2/FA14:0)+NH4	6558.27454	(5218.36, 7898.19)	16112.055	(12319.55, 19904.56)	6.1528E-05	0.00024416	2.45675214
TAG(52:5/FA20:3)+NH4	9807.42456	(7618.15, 11996.7)	26884.6899	(20013.16, 33756.22)	6.4051E-05	0.00025217	2.7412589
TAG(50:3/FA20:3)+NH4	10775.7372	(8288.26, 13263.22)	34259.144	(24999.05, 43519.24)	6.4743E-05	0.0002529	3.17928541
TAG(55:5/FA18:1)+NH4	18082.659	(14243.17, 21922.15)	37307.2302	(30127.5, 44486.96)	6.6476E-05	0.00025766	2.06314958
TAG(52:4/FA18:2)+NH4	9998.44867	(7666.53, 12330.37)	19711.9759	(16294.58, 23129.37)	7.1546E-05	0.00027262	1.97150343
TAG(52:6/FA18:2)+NH4	8087.25435	(5971.01, 10203.49)	18267.7799	(14482.85, 22052.71)	7.1604E-05	0.00027262	2.25883583
TAG(49:3/FA18:3)+NH4	7107.74958	(5887.05, 8328.45)	12969.5104	(10788.14, 15150.88)	7.2681E-05	0.00027262	1.82469997
TAG(52:7/FA22:6)+NH4	13594.0281	(11000.25, 16187.8)	27030.6656	(21919.43, 32141.91)	7.2972E-05	0.00027262	1.98842208
TAG(50:4/FA18:1)+NH4	20003.052	(15728.69, 24277.41)	35634.5898	(30515.95, 40753.23)	7.3063E-05	0.00027262	1.78145764
TAG(44:3/FA18:2)+NH4	8346.41357	(6103.64, 10589.18)	19221.1218	(15154.0, 23288.25)	7.4128E-05	0.00027441	2.30291989
TAG(56:6/FA18:0)+NH4	13679.6907	(10370.47, 16988.91)	40242.7184	(29384.87, 51100.57)	7.4639E-05	0.00027441	2.9417857
DAG(16:0/20:5)+NH4	72603.1409	(52365.13, 92841.15)	171473.594	(134227.58, 208719.61)	7.7876E-05	0.00028422	2.36179306
TAG(52:2/FA16:1)+NH4	11546.5074	(8302.04, 14790.97)	27075.6985	(21253.38, 32898.01)	7.899E-05	0.00028559	2.3449254
TAG(56:8/FA18:3)+NH4	14892.3919	(10810.94, 18973.84)	33977.8627	(26763.81, 41191.91)	7.9658E-05	0.00028559	2.28155845
TAG(48:3/FA18:1)+NH4	15864.9306	(11909.1, 19820.77)	30782.779	(25736.03, 35829.53)	8.05E-05	0.00028559	1.94030341
TAG(52:5/FA18:3)+NH4	8403.53369	(6781.73, 10025.34)	24164.6447	(17662.63, 30666.66)	8.0535E-05	0.00028559	2.87553374
TAG(58:7/FA22:6)+NH4	15938.8926	(11607.86, 20269.93)	35973.813	(28494.91, 43452.71)	8.4223E-05	0.00029656	2.25698321
TAG(52:7/FA20:5)+NH4	10065.9619	(7661.04, 12470.88)	21191.4976	(17014.54, 25368.46)	8.8939E-05	0.00031098	2.10526305
TAG(44:2/FA16:0)+NH4	8161.80665	(5806.51, 10517.1)	20733.1944	(15817.91, 25648.48)	8.9825E-05	0.00031189	2.54027023
TAG(60:10/FA22:5)+NH4	14748.0956	(12463.1, 17033.09)	36190.9506	(27149.35, 45232.55)	9.3447E-05	0.00032223	2.45394061
TAG(51:1/FA18:0)+NH4	10605.2564	(7602.82, 13607.69)	21478.7384	(17818.9, 25138.58)	9.4279E-05	0.00032287	2.02529176

TAG(50:3/FA16:1)+NH4	8208.67708	(6513.15, 9904.21)	23613.5805	(16917.2, 30309.96)	0.00010147	0.00034514	2.87666091
TAG(47:0/FA17:0)+NH4	6778.38903	(5620.31, 7936.47)	17550.7539	(12960.43, 22141.08)	0.00010651	0.00035983	2.58922198
TAG(56:4/FA18:0)+NH4	16787.2372	(13327.1, 20247.38)	41790.8503	(31344.74, 52236.96)	0.00010844	0.00036223	2.48944182
TAG(51:3/FA16:1)+NH4	8459.89851	(6553.72, 10366.08)	25108.0481	(18285.29, 31930.81)	0.00010867	0.00036223	2.96788999
TAG(53:4/FA18:2)+NH4	8804.13773	(7000.8, 10607.47)	22283.7435	(16815.55, 27751.94)	0.00011169	0.00036985	2.53105349
TAG(52:6/FA16:1)+NH4	7549.87534	(5420.95, 9678.8)	16000.3547	(13090.8, 18909.91)	0.00011449	0.00037661	2.11928726
DAG(18:0/18:3)+NH4	159461.47	(109168.12, 209754.82)	313850.206	(267407.85, 360292.56)	0.00011897	0.00038879	1.96818834
TAG(56:5/FA18:2)+NH4	12369.569	(8819.17, 15919.97)	25479.2574	(20919.14, 30039.37)	0.00012584	0.00040857	2.05983388
TAG(53:0/FA16:0)+NH4	10193.1933	(8386.09, 12000.29)	22449.6197	(17339.73, 27559.51)	0.00013067	0.00042076	2.20241282
CE(20:1)+H	10873.5763	(6824.22, 14922.93)	22477.1218	(19236.32, 25717.93)	0.00013132	0.00042076	2.0671324
TAG(52:5/FA16:1)+NH4	7996.99996	(6005.65, 9988.35)	21033.8911	(15934.99, 26132.8)	0.00013212	0.00042076	2.63022274
TAG(56:9/FA18:3)+NH4	16946.9788	(13187.95, 20706.01)	43761.2569	(32357.32, 55165.2)	0.00013431	0.00042504	2.58224534
TAG(58:6/FA20:4)+NH4	16722.253	(13117.81, 20326.7)	58009.6078	(39859.0, 76160.22)	0.00013578	0.00042699	3.46900671
TAG(51:4/FA20:4)+NH4	8337.41934	(6286.55, 10388.29)	15932.286	(13208.49, 18656.08)	0.0001385	0.00043274	1.91093734
TAG(60:11/FA22:5)+NH4	18218.0222	(13879.56, 22556.49)	41720.7771	(32097.15, 51344.4)	0.00013934	0.00043274	2.29008268
TAG(48:5/FA18:3)+NH4	8798.02196	(7013.09, 10582.95)	19865.4626	(15220.72, 24510.21)	0.00014103	0.00043528	2.25794647
TAG(49:2/FA17:0)+NH4	8957.56258	(6123.33, 11791.8)	25325.1837	(18508.8, 32141.56)	0.00014655	0.00044953	2.8272405
TAG(52:5/FA22:5)+NH4	8917.40576	(7165.05, 10669.76)	16936.7944	(13751.56, 20122.03)	0.00015589	0.00047526	1.89929614
CE(20:5)+H	13983.222	(11185.15, 16781.3)	28887.8108	(22730.51, 35045.11)	0.00015771	0.0004779	2.06589088
TAG(52:5/FA16:0)+NH4	8525.55643	(6796.83, 10254.28)	25889.4514	(18152.01, 33626.9)	0.00016988	0.00051169	3.03668759
TAG(48:4/FA18:2)+NH4	10552.9434	(9195.4, 11910.48)	27711.5896	(19975.1, 35448.08)	0.00017518	0.00052449	2.62595833
TAG(58:10/FA22:6)+NH4	11757.5934	(9082.95, 14432.23)	24307.0943	(19218.86, 29395.33)	0.0001765	0.00052528	2.06735286
LCER(24:1)+H	41971.6687	(30026.46, 53916.87)	115246.227	(82954.35, 147538.1)	0.00018083	0.00053501	2.74581
TAG(52:4/FA20:3)+NH4	9205.52923	(7705.41, 10705.65)	29645.7651	(20371.07, 38920.46)	0.0001839	0.00053916	3.22043028
TAG(54:4/FA20:2)+NH4	10204.207	(7729.51, 12678.9)	20375.5587	(16407.8, 24343.32)	0.00018439	0.00053916	1.99678022

TAG(54:4/FA16:1)+NH4	13521.1388	(11707.09, 15335.19)	29843.5922	(22535.27, 37151.92)	0.00019207	0.00055721	2.20718037
DAG(16:0/18:1)+NH4	299053.793	(211644.08, 386463.5)	664343.517	(520201.55, 808485.49)	0.0001928	0.00055721	2.22148501
TAG(52:4/FA18:3)+NH4	13101.2969	(10427.76, 15774.83)	30585.7835	(23191.93, 37979.64)	0.00020408	0.00058643	2.33456151
TAG(54:5/FA20:2)+NH4	12131.5614	(9997.65, 14265.47)	24284.8965	(19234.75, 29335.04)	0.00020623	0.00058922	2.0017948
TAG(54:1/FA16:0)+NH4	14385.9867	(10653.88, 18118.09)	28350.7872	(23046.38, 33655.19)	0.00020783	0.00059043	1.97072247
TAG(46:1/FA18:0)+NH4	11286.9278	(7016.5, 15557.35)	24723.8335	(20229.73, 29217.94)	0.00020984	0.00059132	2.19048389
CE(22:1)+H	10542.3236	(8244.65, 12840.0)	24953.5457	(18659.38, 31247.71)	0.00021051	0.00059132	2.36698726
TAG(53:4/FA18:3)+NH4	8335.90253	(6791.87, 9879.94)	20226.5914	(14901.37, 25551.81)	0.00021775	0.00060663	2.42644289
SM(18:0)+H	2958954.77	(1984077.5, 3933832.04)	9024497.68	(6550965.05, 11498030.3)	0.00021839	0.00060663	3.04989376
TAG(48:2/FA16:0)+NH4	8267.60686	(6145.49, 10389.72)	18238.4909	(14088.5, 22388.48)	0.00022423	0.00061943	2.20601817
TAG(49:3/FA16:1)+NH4	7772.61814	(5973.41, 9571.82)	22320.3942	(15967.92, 28672.87)	0.00023402	0.00064233	2.87166999
TAG(49:2/FA16:0)+NH4	8408.03992	(5430.42, 11385.66)	22540.6878	(16612.94, 28468.43)	0.00023509	0.00064233	2.68084928
PE(P-18:0/20:2)-H	46316125.5	(42404875.48, 50227375.58)	63031870.8	(56207610.98, 69856130.64)	0.00024197	0.00065429	1.36090552
TAG(53:2/FA18:2)+NH4	8286.51587	(6122.05, 10450.99)	16614.4404	(13347.52, 19881.36)	0.00024209	0.00065429	2.00499711
TAG(47:1/FA16:1)+NH4	9215.35456	(6886.7, 11544.01)	19319.534	(15155.25, 23483.82)	0.00025188	0.0006771	2.09645043
TAG(52:2/FA18:0)+NH4	11240.1319	(6726.37, 15753.9)	24011.6177	(20088.77, 27934.46)	0.00025723	0.00068779	2.13623985
TAG(56:6/FA18:1)+NH4	14008.5828	(10698.37, 17318.8)	27768.7847	(22153.47, 33384.1)	0.00026132	0.000695	1.98226937
TAG(52:3/FA18:3)+NH4	8560.96359	(6116.22, 11005.7)	18132.1584	(14398.08, 21866.23)	0.00027041	0.00071536	2.11800438
TAG(58:6/FA22:5)+NH4	22717.1476	(17674.25, 27760.04)	46100.333	(36037.88, 56162.79)	0.00031325	0.00082433	2.02931873
TAG(55:4/FA18:2)+NH4	11361.7375	(9142.77, 13580.71)	22557.1061	(17502.23, 27611.98)	0.00033681	0.0008817	1.9853571
TAG(52:1/FA20:1)+NH4	12109.8819	(10052.13, 14167.63)	19678.5887	(16618.24, 22738.94)	0.00035884	0.00093448	1.62500253
TAG(60:10/FA22:6)+NH4	21193.7119	(16397.76, 25989.67)	45344.0934	(34550.28, 56137.91)	0.00037398	0.00096418	2.13950692
TAG(50:3/FA16:0)+NH4	6700.21127	(4759.52, 8640.91)	18586.9433	(13290.5, 23883.39)	0.0003741	0.00096418	2.77408317
TAG(54:4/FA20:3)+NH4	10128.87	(7920.82, 12336.92)	27224.6115	(19146.18, 35303.04)	0.00038076	0.00097632	2.68782319
LCER(d18:0/20:0)+H	45117.7457	(37588.36, 52647.13)	77718.9465	(63573.42, 91864.48)	0.00039517	0.00100148	1.72258044

TAG(45:1/FA16:0)+NH4	6675.03178	(4923.63, 8426.43)	16315.9994	(11911.25, 20720.75)	0.00039636	0.00100148	2.44433284
TAG(52:3/FA18:2)+NH4	13152.1347	(10923.98, 15380.29)	24341.8388	(19311.29, 29372.39)	0.00039659	0.00100148	1.85078995
TAG(58:9/FA20:4)+NH4	16217.1061	(12304.69, 20129.52)	35579.9678	(26872.6, 44287.34)	0.00040828	0.00102525	2.19397761
TAG(52:6/FA16:0)+NH4	8972.0916	(6634.46, 11309.72)	23572.818	(16761.65, 30383.99)	0.0004101	0.00102525	2.62734923
TAG(54:7/FA18:2)+NH4	8031.79005	(6452.74, 9610.84)	17701.9654	(13158.58, 22245.35)	0.0004496	0.0011184	2.20398757
TAG(52:6/FA18:3)+NH4	9696.39505	(8326.45, 11066.34)	19122.9037	(14633.86, 23611.94)	0.00045444	0.00111935	1.97216632
DAG(18:0/18:2)+NH4	473282.152	(361525.16, 585039.15)	799671.774	(681690.9, 917652.65)	0.00045446	0.00111935	1.68963011
TAG(52:8/FA16:1)+NH4	8065.05406	(6027.21, 10102.9)	14888.4888	(12150.63, 17626.34)	0.00047746	0.00116532	1.84604947
TAG(50:4/FA18:3)+NH4	18086.3039	(13545.32, 22627.28)	32932.3246	(27056.15, 38808.5)	0.00047778	0.00116532	1.82084327
TAG(44:2/FA14:0)+NH4	5947.79591	(4773.05, 7122.54)	12080.2565	(9334.2, 14826.31)	0.00048302	0.00117195	2.03104758
TAG(55:1/FA16:0)+NH4	10431.1931	(7895.22, 12967.17)	21417.2596	(16440.88, 26393.64)	0.00048519	0.00117195	2.05319367
TAG(54:2/FA18:0)+NH4	19753.8174	(10914.49, 28593.14)	44233.8917	(35641.32, 52826.46)	0.00051306	0.00123332	2.23925791
TAG(44:1/FA14:0)+NH4	5264.19825	(4116.71, 6411.69)	9867.74045	(7849.56, 11885.93)	0.00052108	0.00124201	1.87450016
HCER(d18:0/26:1)+H	2970.32334	(1677.8, 4262.84)	12101.0382	(7721.49, 16480.58)	0.00052165	0.00124201	4.07398012
TAG(54:6/FA20:4)+NH4	9551.29812	(7285.44, 11817.15)	19687.7666	(15099.54, 24275.99)	0.00052687	0.00124851	2.06126606
CE(20:3)+H	14047.8996	(9656.22, 18439.58)	31640.7136	(23914.39, 39367.04)	0.00053054	0.00125127	2.2523448
TAG(52:5/FA20:4)+NH4	9890.7932	(7215.48, 12566.1)	27769.4714	(17769.67, 37769.27)	0.00053954	0.00126653	2.80760813
TAG(58:7/FA22:4)+NH4	12891.375	(9727.13, 16055.62)	25257.1021	(19996.35, 30517.86)	0.00054838	0.00128126	1.95922484
TAG(58:3/FA18:1)+NH4	11334.7493	(9278.82, 13390.68)	19425.1185	(15871.87, 22978.37)	0.00055606	0.00129316	1.71376693
TAG(56:5/FA20:4)+NH4	16274.69	(11847.17, 20702.21)	30833.0442	(24901.83, 36764.25)	0.00056755	0.00131378	1.89453957
TAG(56:6/FA22:5)+NH4	19390.8217	(14140.57, 24641.07)	43803.8652	(32503.89, 55103.84)	0.00059122	0.00136226	2.25899995
TAG(54:1/FA20:1)+NH4	12144.4772	(10326.87, 13962.09)	20174.8607	(16492.23, 23857.5)	0.00060362	0.00138446	1.66123748
TAG(56:7/FA16:1)+NH4	36014.8182	(27143.38, 44886.25)	77885.3223	(58264.7, 97505.95)	0.00063983	0.0014608	2.16259102
TAG(46:3/FA18:2)+NH4	21991.9588	(17112.88, 26871.03)	53199.8974	(38507.3, 67892.49)	0.00070366	0.00159922	2.41906134
TAG(52:2/FA14:0)+NH4	7059.49404	(5557.54, 8561.45)	14591.4661	(10975.04, 18207.89)	0.00071566	0.00161914	2.06692803

TAG(56:5/FA16:0)+NH4	17035.0488	(13144.79, 20925.31)	54553.8738	(36105.7, 73002.05)	0.00071966	0.00162086	3.20244894
TAG(46:3/FA16:0)+NH4	9407.11218	(7122.16, 11692.06)	20928.0818	(15377.49, 26478.67)	0.00073128	0.00163965	2.22470844
TAG(54:5/FA16:0)+NH4	13417.8341	(10103.73, 16731.94)	32464.1176	(23149.96, 41778.27)	0.00076349	0.00170422	2.41947526
TAG(52:3/FA20:1)+NH4	8109.45144	(6213.92, 10004.98)	19562.9565	(14058.73, 25067.19)	0.00077535	0.00172301	2.41236496
TAG(46:1/FA16:1)+NH4	7939.00879	(5974.48, 9903.54)	16621.9331	(12769.16, 20474.7)	0.00077998	0.00172563	2.09370383
TAG(52:4/FA20:0)+NH4	10852.5615	(8854.99, 12850.13)	19731.3484	(15513.48, 23949.22)	0.00079983	0.00176173	1.81812822
CE(22:0)+H	8436.50276	(6438.42, 10434.58)	23675.9311	(15904.98, 31446.89)	0.00081337	0.00178371	2.80636796
TAG(48:1/FA16:1)+NH4	9533.45537	(6004.98, 13061.93)	20754.673	(16100.32, 25409.03)	0.00083248	0.00181765	2.17703574
DAG(18:2/18:3)+NH4	27520.5275	(20013.5, 35027.55)	52284.2971	(41572.57, 62996.03)	0.00084006	0.00182623	1.89982903
TAG(46:1/FA18:1)+NH4	20958.5041	(16065.77, 25851.24)	38522.6984	(30502.0, 46543.4)	0.00085678	0.00185451	1.83804618
TAG(52:1/FA20:0)+NH4	10866.9548	(8727.8, 13006.11)	18539.4261	(15169.58, 21909.27)	0.000871	0.00187137	1.70603693
TAG(58:7/FA20:4)+NH4	18089.6507	(14484.49, 21694.81)	33092.9715	(26002.3, 40183.65)	0.00087206	0.00187137	1.82938698
TAG(48:4/FA20:4)+NH4	11646.8096	(9039.68, 14253.94)	25155.2904	(18481.1, 31829.48)	0.0008761	0.00187201	2.15984388
TAG(58:8/FA22:5)+NH4	11642.1047	(9206.39, 14077.82)	20863.7692	(16732.98, 24994.56)	0.00088384	0.00188052	1.792096
TAG(56:6/FA18:3)+NH4	16832.6154	(12656.2, 21009.03)	36169.6111	(26782.79, 45556.43)	0.00089068	0.00188704	2.14878142
TAG(54:4/FA20:1)+NH4	12509.2262	(9449.75, 15568.7)	25467.6653	(19465.17, 31470.16)	0.00091156	0.00192312	2.03591054
TAG(51:5/FA18:2)+NH4	8400.71212	(6662.26, 10139.16)	20025.7374	(14074.64, 25976.84)	0.00092458	0.00194239	2.38381427
TAG(56:5/FA20:1)+NH4	10028.5077	(7441.03, 12615.99)	22145.4837	(16390.69, 27900.27)	0.00094828	0.00198384	2.20825315
TAG(55:3/FA18:1)+NH4	13332.195	(10310.46, 16353.93)	24132.5606	(19194.73, 29070.39)	0.00097159	0.00201632	1.81009658
TAG(49:3/FA16:0)+NH4	8035.75411	(6180.34, 9891.17)	18109.4144	(13037.08, 23181.74)	0.00097424	0.00201632	2.25360485
PE(P-18:0/18:1)-H	722930140	(661654720.64, 784205559.33)	885322154	(823446292.93, 947198014.43)	0.0009759	0.00201632	1.2246303
TAG(54:3/FA16:0)+NH4	13795.994	(10560.19, 17031.79)	30661.141	(22202.35, 39119.93)	0.00098944	0.00203106	2.22246698
CE(18:3)+H	34485.5443	(26990.03, 41981.06)	72457.6202	(53490.37, 91424.87)	0.00099115	0.00203106	2.10110125
TAG(53:3/FA16:0)+NH4	11560.0258	(8448.26, 14671.79)	32479.6981	(22041.4, 42917.99)	0.00099938	0.00203954	2.80965619
TAG(58:8/FA18:2)+NH4	38131.8532	(25928.79, 50334.92)	113181.185	(74660.74, 151701.63)	0.00101515	0.00206331	2.96815327

TAG(56:7/FA18:3)+NH4	23513.0039	(17320.38, 29705.63)	44967.8871	(35454.45, 54481.32)	0.00102097	0.00206674	1.91246883
TAG(53:4/FA16:0)+NH4	15157.6562	(9189.1, 21126.22)	35966.4484	(26708.06, 45224.84)	0.00102895	0.0020745	2.37282386
TAG(52:2/FA18:1)+NH4	35595.24	(16257.58, 54932.9)	78919.284	(65784.53, 92054.03)	0.00103686	0.00208205	2.21713027
TAG(54:2/FA18:2)+NH4	10024.9715	(7851.12, 12198.82)	19200.0958	(14741.93, 23658.26)	0.00105587	0.00211174	1.91522697
TAG(52:4/FA20:2)+NH4	8178.00101	(6383.43, 9972.57)	17010.798	(12575.97, 21445.62)	0.00107599	0.0021434	2.08006798
TAG(52:3/FA20:2)+NH4	8517.57319	(6608.52, 10426.62)	14240.0365	(11792.37, 16687.7)	0.00109167	0.002166	1.67184199
LCER(d18:0/24:1)+H	32361.9379	(24111.53, 40612.34)	56159.1387	(46212.68, 66105.6)	0.00110337	0.00218058	1.73534535
TAG(54:6/FA18:1)+NH4	10965.4381	(9041.38, 12889.49)	20019.302	(15617.85, 24420.75)	0.00114195	0.00224793	1.8256728
TAG(53:2/FA17:0)+NH4	19235.3405	(12754.97, 25715.71)	40880.5524	(31120.73, 50640.38)	0.00114835	0.00225167	2.12528353
TAG(49:1/FA14:0)+NH4	6781.90004	(5231.54, 8332.26)	13566.6088	(10257.13, 16876.09)	0.00130286	0.00253548	2.00041415
TAG(58:9/FA22:5)+NH4	11860.3502	(9145.49, 14575.21)	19451.6886	(16253.86, 22649.51)	0.00130324	0.00253548	1.64006022
TAG(54:5/FA18:2)+NH4	11796.6637	(9985.56, 13607.76)	18196.595	(15155.32, 21237.87)	0.0013145	0.00254749	1.54252045
TAG(53:3/FA17:0)+NH4	11716.7467	(6792.42, 16641.07)	33356.5375	(22535.93, 44177.14)	0.00132147	0.00255109	2.84691122
DAG(18:1/22:4)+NH4	31531.6737	(21921.79, 41141.56)	65653.851	(49384.59, 81923.11)	0.00132974	0.00255719	2.0821556
TAG(50:2/FA16:0)+NH4	15975.7255	(11788.46, 20162.99)	32296.9175	(24357.03, 40236.81)	0.00133507	0.00255761	2.02162446
TAG(54:3/FA18:1)+NH4	32441.4535	(21871.69, 43011.22)	67707.6762	(51176.71, 84238.64)	0.00138996	0.00265259	2.08707283
TAG(54:3/FA18:3)+NH4	10127.9324	(7724.34, 12531.52)	18193.0941	(14396.4, 21989.79)	0.00140838	0.00267753	1.79632855
TAG(46:2/FA18:1)+NH4	41333.5545	(27584.49, 55082.62)	81589.1173	(63795.85, 99382.39)	0.00144257	0.00273214	1.97391969
TAG(44:0/FA18:0)+NH4	8031.90848	(4174.41, 11889.41)	21286.0315	(15017.27, 27554.79)	0.00146042	0.00275552	2.65018352
TAG(54:7/FA18:1)+NH4	10844.2304	(7232.35, 14456.11)	23845.7352	(17520.62, 30170.85)	0.00148227	0.00277192	2.19893292
TAG(56:8/FA18:1)+NH4	11915.3792	(9587.4, 14243.36)	25772.0251	(18365.98, 33178.07)	0.00148317	0.00277192	2.16292111
TAG(51:1/FA16:0)+NH4	10637.243	(8690.39, 12584.1)	20438.5706	(15175.65, 25701.49)	0.00148584	0.00277192	1.92141617
TAG(54:5/FA22:4)+NH4	11407.7751	(8501.32, 14314.23)	19170.0817	(15931.6, 22408.56)	0.00149129	0.00277192	1.68044
TAG(44:2/FA16:1)+NH4	5883.16497	(4091.88, 7674.45)	13732.3087	(9704.41, 17760.21)	0.00151696	0.00280919	2.33417025
TAG(53:1/FA16:0)+NH4	9759.22153	(7545.43, 11973.02)	18610.3416	(14418.51, 22802.18)	0.00153869	0.0028389	1.9069494

TAG(54:8/FA20:5)+NH4	9320.38184	(6895.0, 11745.76)	19613.4982	(14230.76, 24996.23)	0.00155916	0.0028661	2.10436638
DAG(18:2/22:5)+NH4	9547.50593	(8006.83, 11088.18)	16996.2407	(13200.73, 20791.75)	0.00161476	0.00295743	1.78017598
TAG(55:5/FA20:4)+NH4	9385.53463	(7806.24, 10964.82)	18200.9376	(13454.25, 22947.62)	0.00166867	0.00304502	1.93925421
TAG(51:2/FA18:1)+NH4	11985.567	(8126.38, 15844.76)	27754.8117	(19675.88, 35833.74)	0.0016767	0.00304855	2.31568616
TAG(54:4/FA16:0)+NH4	19986.725	(15887.53, 24085.92)	65227.2022	(39851.03, 90603.38)	0.00168794	0.00305786	3.26352628
TAG(56:3/FA18:0)+NH4	14971.8311	(11625.24, 18318.42)	31065.3265	(22304.64, 39826.01)	0.00175365	0.00316544	2.0749183
TAG(52:2/FA20:1)+NH4	10152.8149	(7640.04, 12665.59)	18871.8475	(14572.41, 23171.29)	0.0017696	0.00318273	1.85877984
TAG(53:1/FA17:0)+NH4	14701.5042	(11209.18, 18193.83)	34559.5922	(24471.55, 44647.64)	0.00180545	0.00323558	2.35075212
CE(20:2)+H	16537.5313	(11587.68, 21487.39)	29334.9881	(23924.17, 34745.8)	0.00182296	0.00325206	1.77384324
TAG(51:5/FA18:3)+NH4	9919.93924	(8107.91, 11731.96)	21282.4901	(15026.68, 27538.3)	0.00182766	0.00325206	2.14542544
TAG(52:7/FA16:0)+NH4	12595.738	(9589.9, 15601.58)	24084.521	(18187.17, 29981.88)	0.0019139	0.00339344	1.9121167
DCER(20:0)+H	229214.509	(181642.28, 276786.74)	351392.194	(299418.41, 403365.98)	0.0019301	0.00340843	1.53302771
TAG(51:4/FA18:3)+NH4	8567.88931	(6996.69, 10139.09)	14629.9645	(11505.68, 17754.25)	0.00193599	0.00340843	1.70753426
TAG(50:2/FA16:1)+NH4	8723.56226	(6630.41, 10816.71)	24069.2529	(15758.57, 32379.94)	0.00194385	0.00341026	2.75910828
CE(16:0)+H	23136.0305	(16334.68, 29937.38)	41310.2356	(33298.71, 49321.76)	0.00195805	0.00342316	1.78553688
TAG(56:4/FA18:2)+NH4	10417.1626	(7383.63, 13450.69)	21323.9439	(16112.19, 26535.7)	0.00196648	0.00342593	2.04700117
TAG(56:3/FA20:1)+NH4	19435.3335	(14717.5, 24153.16)	36455.7184	(27635.47, 45275.96)	0.00200698	0.00348433	1.87574442
TAG(42:1/FA18:1)+NH4	9558.83733	(7061.62, 12056.05)	22829.5299	(15187.38, 30471.68)	0.00203194	0.00351546	2.3883166
TAG(54:5/FA16:1)+NH4	20598.6615	(13364.1, 27833.22)	40414.6175	(31470.95, 49358.28)	0.00204704	0.00352937	1.96200212
TAG(50:4/FA14:0)+NH4	12422.0656	(10050.08, 14794.05)	23383.952	(17330.83, 29437.08)	0.00205908	0.00353794	1.88245278
TAG(58:6/FA16:0)+NH4	12434.4866	(9460.17, 15408.8)	22102.3761	(17568.96, 26635.79)	0.00211032	0.00358822	1.77750613
TAG(56:9/FA20:4)+NH4	8959.34871	(6311.53, 11607.16)	16479.8221	(13058.13, 19901.52)	0.00211293	0.00358822	1.83939956
TAG(56:7/FA22:6)+NH4	19653.7612	(15864.12, 23443.41)	36783.1639	(27326.82, 46239.51)	0.00211694	0.00358822	1.87155851
CE(22:5)+H	17780.0462	(13315.98, 22244.12)	31888.8802	(24984.82, 38792.94)	0.00211705	0.00358822	1.79352065
TAG(44:1/FA16:1)+NH4	5903.19653	(4685.66, 7120.73)	12524.1246	(8842.66, 16205.59)	0.00221246	0.00373726	2.12158354

DAG(18:2/22:6)+NH4	15173.5669	(10619.21, 19727.92)	26824.7004	(21708.61, 31940.79)	0.0022873	0.00385068	1.76785726
TAG(52:4/FA16:0)+NH4	11776.0107	(9033.31, 14518.71)	26601.0408	(18588.15, 34613.93)	0.00231271	0.00388038	2.25891785
TAG(50:6/FA20:4)+NH4	10844.198	(8366.05, 13322.35)	19855.3349	(15155.11, 24555.56)	0.00234796	0.00392635	1.83096389
TAG(54:6/FA16:1)+NH4	13543.1055	(10576.32, 16509.89)	23390.7023	(18374.61, 28406.79)	0.00242268	0.00403779	1.72712988
TAG(46:2/FA14:0)+NH4	7252.51404	(5899.23, 8605.8)	13599.7386	(10090.81, 17108.67)	0.00244827	0.00406689	1.87517576
TAG(51:1/FA17:0)+NH4	11211.0263	(8901.81, 13520.24)	26069.765	(17363.49, 34776.04)	0.0024938	0.0041288	2.32536829
TAG(46:4/FA18:2)+NH4	11140.1085	(7466.49, 14813.72)	20120.9648	(16325.32, 23916.61)	0.00250792	0.00413848	1.80617315
TAG(56:7/FA20:3)+NH4	10529.973	(7701.43, 13358.51)	19844.9345	(15190.48, 24499.39)	0.00257856	0.00424106	1.884614
TAG(54:3/FA20:2)+NH4	12135.5804	(9841.06, 14430.1)	26689.2907	(18304.52, 35074.06)	0.00262337	0.0043006	2.19925952
LPG(20:1)-H	3148490.24	(2648646.94, 3648333.54)	4477068.77	(3860256.79, 5093880.76)	0.00263306	0.00430238	1.42197321
TAG(58:10/FA22:5)+NH4	8925.21654	(6720.28, 11130.15)	14703.6988	(12044.55, 17362.85)	0.00264191	0.00430279	1.64743328
TAG(48:1/FA16:0)+NH4	14661.8103	(9903.87, 19419.75)	30823.7215	(22239.78, 39407.66)	0.00268414	0.00435737	2.10231348
TAG(42:1/FA16:0)+NH4	7945.55334	(5351.58, 10539.53)	16463.1854	(12300.16, 20626.21)	0.00271041	0.00438577	2.07199986
TAG(56:7/FA18:0)+NH4	10459.1137	(7892.62, 13025.61)	19800.0818	(14933.87, 24666.29)	0.00282069	0.00454949	1.89309366
TAG(52:6/FA20:5)+NH4	8181.85687	(6054.31, 10309.41)	19301.6552	(12565.96, 26037.35)	0.00292297	0.00469932	2.35908004
TAG(56:10/FA18:2)+NH4	12376.0735	(7652.08, 17100.07)	23021.6707	(18627.42, 27415.92)	0.00296607	0.00474863	1.86017566
TAG(53:2/FA18:1)+NH4	14634.7766	(11503.75, 17765.8)	23629.0451	(19262.64, 27995.45)	0.00297264	0.00474863	1.61458188
TAG(52:4/FA18:1)+NH4	11273.0809	(8648.46, 13897.7)	19905.2241	(15361.9, 24448.55)	0.00303983	0.00484049	1.76573062
TAG(49:1/FA16:1)+NH4	9148.69846	(6580.89, 11716.51)	17500.2352	(13107.94, 21892.53)	0.0030973	0.00491636	1.91286611
TAG(54:0/FA16:0)+NH4	15600.6764	(10717.03, 20484.32)	27989.101	(22317.22, 33660.98)	0.00311507	0.00492891	1.79409535
TAG(50:3/FA18:2)+NH4	7240.24896	(5933.8, 8546.7)	16848.3466	(11297.87, 22398.82)	0.00327792	0.00517023	2.32703968
TAG(52:2/FA20:0)+NH4	12170.6798	(9631.5, 14709.86)	18920.1157	(15617.98, 22222.25)	0.00344665	0.00541926	1.55456523
TAG(56:6/FA18:2)+NH4	20999.4044	(16337.36, 25661.45)	56756.3871	(35087.85, 78424.92)	0.00357111	0.00559736	2.70276175
TAG(54:4/FA18:0)+NH4	11613.4045	(9107.55, 14119.26)	19970.0132	(15401.19, 24538.84)	0.00374729	0.00585513	1.7195658
TAG(46:2/FA16:1)+NH4	10104.8727	(6751.78, 13457.97)	24857.9857	(16526.23, 33189.74)	0.00385113	0.00599864	2.45999988

TAG(52:5/FA18:2)+NH4	9859.55532	(7665.2, 12053.91)	21360.929	(14502.59, 28219.27)	0.00387047	0.00601005	2.16652052
TAG(50:2/FA18:2)+NH4	10516.9554	(8353.52, 12680.39)	17239.9399	(13577.05, 20902.83)	0.00421112	0.00651876	1.63925197
TAG(50:0/FA14:0)+NH4	8165.13941	(7038.74, 9291.54)	12855.9879	(10096.91, 15615.06)	0.00434616	0.00670704	1.57449705
TAG(52:8/FA18:2)+NH4	10525.7204	(7851.35, 13200.09)	17678.2735	(13995.85, 21360.69)	0.00439892	0.00676757	1.67953098
TAG(54:3/FA20:3)+NH4	10257.3029	(7725.38, 12789.23)	18420.2039	(14109.23, 22731.18)	0.00455942	0.00699297	1.79581358
TAG(54:2/FA20:2)+NH4	10123.0727	(7566.97, 12679.18)	16973.7363	(13401.17, 20546.3)	0.00467171	0.00714329	1.67673757
TAG(56:1/FA16:0)+NH4	12536.5994	(9909.6, 15163.6)	19192.1219	(15807.96, 22576.29)	0.00481243	0.00733603	1.53088739
TAG(51:2/FA16:1)+NH4	9607.47136	(7508.73, 11706.21)	17376.8524	(12965.23, 21788.48)	0.00484982	0.00737054	1.80868116
TAG(45:0/FA14:0)+NH4	6333.37495	(3801.29, 8865.46)	14753.5599	(9935.21, 19571.91)	0.00497249	0.00752906	2.32949415
LPG(22:4)-H	771554.362	(664055.74, 879052.99)	1131151.14	(924961.3, 1337340.98)	0.00498424	0.00752906	1.46606797
TAG(50:4/FA18:2)+NH4	15050.1496	(11761.77, 18338.53)	26768.4547	(19929.15, 33607.76)	0.00504107	0.00759197	1.77861719
TAG(52:1/FA16:1)+NH4	10028.8801	(8458.2, 11599.56)	16639.8543	(12764.3, 20515.41)	0.00518228	0.0077812	1.65919366
TAG(48:0/FA14:0)+NH4	8945.06212	(5421.79, 12468.34)	16520.7731	(13091.88, 19949.67)	0.00523544	0.00783748	1.84691542
TAG(57:2/FA18:1)+NH4	12089.8942	(9951.49, 14228.3)	18134.4458	(14824.95, 21443.94)	0.00529925	0.0078876	1.49996728
TAG(48:4/FA16:1)+NH4	11727.8455	(9231.3, 14224.39)	19719.2901	(15269.73, 24168.85)	0.00530046	0.0078876	1.68140773
TAG(46:2/FA16:0)+NH4	7675.04756	(6034.26, 9315.84)	14660.7443	(10398.5, 18922.99)	0.00541827	0.00803898	1.91018286
TAG(47:2/FA14:0)+NH4	8047.29878	(6182.81, 9911.79)	15441.8243	(11244.95, 19638.7)	0.00553439	0.00818696	1.91888294
TAG(52:2/FA16:0)+NH4	23991.9556	(17030.86, 30953.05)	66834.9549	(38755.1, 94914.81)	0.00565312	0.00833794	2.78572352
TAG(50:5/FA16:1)+NH4	12407.694	(10324.08, 14491.31)	23010.7217	(16394.97, 29626.47)	0.00567052	0.008339	1.85455264
TAG(53:2/FA16:0)+NH4	12049.9753	(9738.85, 14361.1)	21723.5878	(15585.31, 27861.86)	0.00603224	0.00884492	1.80279106
LPG(20:2)-H	1784974.21	(1512224.69, 2057723.73)	2679610.56	(2151878.18, 3207342.94)	0.00608304	0.00889333	1.50120408
TAG(56:7/FA16:0)+NH4	12657.873	(9636.85, 15678.89)	40029.1605	(21463.14, 58595.18)	0.00632757	0.00922386	3.16239234
TAG(54:3/FA20:1)+NH4	10865.6902	(8541.86, 13189.53)	21434.7808	(14724.42, 28145.14)	0.00663188	0.00963646	1.97270311
TAG(54:3/FA16:1)+NH4	12148.3519	(10043.33, 14253.37)	20235.9901	(15224.09, 25247.89)	0.00664916	0.00963646	1.66573953
TAG(51:0/FA17:0)+NH4	9934.22089	(7980.57, 11887.87)	16365.8974	(12452.79, 20279.0)	0.00723102	0.01044946	1.64742637

TAG(48:2/FA18:2)+NH4	9954.24641	(7637.36, 12271.14)	15676.4619	(12543.63, 18809.29)	0.00730083	0.01051058	1.5748517
TAG(56:4/FA20:3)+NH4	18031.2102	(15162.17, 20900.25)	26868.2934	(21718.35, 32018.24)	0.00731536	0.01051058	1.49009929
TAG(48:0/FA18:0)+NH4	10182.7032	(8024.67, 12340.73)	16548.983	(12760.99, 20336.97)	0.00759887	0.01088664	1.62520529
TAG(42:1/FA16:1)+NH4	5940.06197	(4029.12, 7851.0)	11293.3755	(8238.11, 14348.64)	0.00764202	0.01091717	1.90122183
TAG(54:7/FA18:3)+NH4	11399.9249	(7202.41, 15597.44)	20767.4685	(15825.43, 25709.51)	0.00819279	0.01167064	1.82171976
TAG(56:7/FA22:4)+NH4	10683.7215	(8517.6, 12849.85)	15812.7534	(12993.22, 18632.29)	0.0082784	0.01175909	1.48007914
TAG(52:3/FA18:0)+NH4	11622.5712	(9479.94, 13765.2)	17611.0244	(14129.16, 21092.88)	0.00845224	0.01197201	1.51524341
TAG(58:9/FA18:2)+NH4	14790.346	(10022.99, 19557.7)	24308.1105	(19729.02, 28887.2)	0.00851201	0.01202261	1.64351195
TAG(56:3/FA18:2)+NH4	12700.7043	(9050.68, 16350.73)	20032.7719	(16407.63, 23657.91)	0.00919358	0.01293723	1.57729614
TAG(53:4/FA20:4)+NH4	10968.8324	(9183.03, 12754.64)	16699.88	(13182.86, 20216.9)	0.00921131	0.01293723	1.52248475
TAG(44:0/FA14:0)+NH4	6401.1235	(4318.08, 8484.17)	13649.3081	(8976.64, 18321.98)	0.00936686	0.01311885	2.13233007
TAG(48:4/FA14:0)+NH4	7275.5486	(5959.9, 8591.2)	11519.1538	(8805.0, 14233.31)	0.00981744	0.01371151	1.58326945
TAG(48:3/FA18:3)+NH4	12332.101	(8676.92, 15987.28)	20854.6776	(16320.41, 25388.94)	0.00990434	0.01379435	1.69108877
TAG(48:3/FA14:0)+NH4	8844.15576	(6088.71, 11599.61)	15300.5066	(11581.27, 19019.74)	0.00996468	0.01383983	1.73001324
TAG(50:1/FA18:1)+NH4	24998.159	(14989.89, 35006.43)	43800.7316	(34759.39, 52842.07)	0.01043572	0.01445391	1.75215829
PS(14:0/22:6)-H	143990.725	(98506.72, 189474.73)	60558.9266	(26412.5, 94705.35)	0.01072056	0.0148074	-2.37769612
TAG(46:0/FA18:0)+NH4	10571.064	(7209.48, 13932.65)	19556.7035	(13898.22, 25215.18)	0.01100211	0.01515443	1.85002224
TAG(48:1/FA14:0)+NH4	12922.2412	(8437.73, 17406.76)	23928.8414	(17254.42, 30603.26)	0.01106283	0.01516844	1.85175628
CE(18:1)+H	27494.3864	(18204.23, 36784.54)	50412.9517	(36293.69, 64532.21)	0.01107296	0.01516844	1.83357253
CE(22:4)+H	34684.1447	(25152.9, 44215.39)	58927.9737	(44183.24, 73672.71)	0.01110978	0.0151773	1.69898881
TAG(51:2/FA18:2)+NH4	9796.21001	(7572.8, 12019.62)	15942.1251	(12073.11, 19811.14)	0.01129928	0.01539411	1.62737682
TAG(56:4/FA16:0)+NH4	18801.8073	(12740.14, 24863.48)	32838.6417	(24629.72, 41047.56)	0.01139075	0.01547656	1.74656835
TAG(48:0/FA16:0)+NH4	27554.7044	(17866.96, 37242.45)	46755.994	(36726.33, 56785.66)	0.011659	0.0157981	1.69684252
TAG(54:4/FA20:4)+NH4	11330.6141	(8487.92, 14173.31)	18803.968	(14130.6, 23477.33)	0.01190218	0.01608403	1.65957183

TAG(56:6/FA22:4)+NH4	11456.3656	(9014.66, 13898.07)	19450.8078	(14087.48, 24814.14)	0.01245257	0.01678244	1.69781661
TAG(56:8/FA16:1)+NH4	15301.8326	(12149.55, 18454.11)	21985.7105	(18232.89, 25738.53)	0.0127501	0.01713724	1.43680244
TAG(54:7/FA20:5)+NH4	10722.7721	(7432.14, 14013.4)	19754.3264	(14073.19, 25435.47)	0.01290485	0.01729873	1.84227793
TAG(54:8/FA22:6)+NH4	9097.80313	(6630.99, 11564.62)	14066.533	(11308.32, 16824.74)	0.0132855	0.01776137	1.54614612
LCER(d18:0/24:0)+H	25931.392	(20765.88, 31096.9)	41830.2524	(31440.8, 52219.71)	0.01350931	0.01801241	1.61311249
TAG(45:0/FA16:0)+NH4	8479.24241	(6830.89, 10127.6)	15364.9844	(10643.92, 20086.04)	0.01357732	0.01805495	1.81207043
TAG(47:1/FA14:0)+NH4	8806.11484	(5960.27, 11651.96)	15768.2297	(11361.83, 20174.63)	0.01369446	0.01816241	1.79060005
TAG(53:1/FA18:0)+NH4	11166.1079	(8187.52, 14144.7)	18192.2807	(13851.29, 22533.27)	0.01379996	0.01825392	1.62924099
TAG(48:1/FA18:1)+NH4	13015.1095	(5262.14, 20768.08)	26472.1378	(19975.89, 32968.39)	0.01407053	0.01852547	2.03395429
TAG(56:5/FA20:3)+NH4	18751.6244	(14207.67, 23295.57)	31737.9188	(23097.95, 40377.89)	0.01407936	0.01852547	1.69254237
TAG(49:2/FA14:0)+NH4	8112.59227	(6097.53, 10127.65)	14428.4832	(10073.48, 18783.48)	0.01502965	0.01972396	1.77852932
LCER(d18:0/18:0)+H	27910.7691	(20036.96, 35784.58)	42490.5786	(34722.16, 50259.0)	0.0156332	0.0204623	1.52237219
TAG(46:3/FA16:1)+NH4	11307.2763	(8142.84, 14471.72)	21737.923	(14614.99, 28860.85)	0.01581105	0.02064106	1.92247208
TAG(49:2/FA18:2)+NH4	7442.98378	(5139.88, 9746.08)	15629.8254	(9755.9, 21503.75)	0.01637569	0.02132251	2.09994081
TAG(46:1/FA14:0)+NH4	8925.47076	(6386.72, 11464.22)	13740.8774	(11007.02, 16474.74)	0.01690108	0.02194945	1.5395129
TAG(51:0/FA18:0)+NH4	10097.6425	(6901.95, 13293.33)	16139.8615	(12699.22, 19580.51)	0.01721095	0.02229398	1.59837917
TAG(47:2/FA16:1)+NH4	10484.9996	(7711.53, 13258.47)	17971.3443	(12849.59, 23093.1)	0.01732188	0.02237923	1.71400525
TAG(52:4/FA20:4)+NH4	8790.0509	(7081.74, 10498.36)	14447.1127	(10634.54, 18259.68)	0.01736628	0.02237923	1.64357555
TAG(54:4/FA22:4)+NH4	9588.47397	(7276.39, 11900.56)	14809.804	(11521.8, 18097.8)	0.01769709	0.02274691	1.54454234
TAG(42:1/FA14:0)+NH4	4906.51392	(3888.09, 5924.94)	7101.2134	(5708.65, 8493.78)	0.01839757	0.02358663	1.44730322
TAG(54:7/FA16:1)+NH4	15930.5771	(7068.87, 24792.29)	28901.6647	(23796.64, 34006.69)	0.01871817	0.02393628	1.81422585
TAG(50:0/FA18:0)+NH4	16714.1691	(12087.1, 21341.24)	27654.6139	(20233.23, 35076.0)	0.01892785	0.02413403	1.6545611
TAG(52:3/FA18:1)+NH4	19109.6057	(12531.93, 25687.28)	30253.559	(24397.51, 36109.61)	0.01896934	0.02413403	1.58315977
TAG(52:5/FA20:5)+NH4	10702.9374	(8611.83, 12794.04)	16475.1129	(12297.61, 20652.61)	0.01968441	0.02498022	1.5393076
TAG(55:2/FA18:2)+NH4	10574.8785	(8815.05, 12334.71)	15309.1508	(11949.43, 18668.87)	0.02049842	0.02594737	1.44769047

TAG(50:4/FA20:4)+NH4	13794.5973	(10419.48, 17169.72)	28212.3311	(17114.98, 39309.69)	0.02099055	0.02650322	2.0451725
TAG(46:0/FA14:0)+NH4	9789.73397	(6386.6, 13192.87)	17172.8367	(12182.53, 22163.14)	0.02187394	0.02754905	1.75416787
TAG(53:5/FA20:4)+NH4	11323.0971	(9037.48, 13608.72)	16890.5992	(13088.42, 20692.78)	0.02203616	0.02768362	1.49169428
PE(O-18:0/20:1)-H	27886674.7	(25169113.6, 30604235.8)	33522055.8	(29907542.42, 37136569.14)	0.02213682	0.02774038	1.2020815
PE(O-16:0/18:0)-H	1390746.75	(1043312.51, 1738181.0)	1931375.84	(1660836.17, 2201915.5)	0.02247689	0.02797165	1.38873295
TAG(42:0/FA14:0)+NH4	5462.32084	(2926.59, 7998.06)	11262.3814	(7208.63, 15316.13)	0.02250327	0.02797165	2.06183081
TAG(42:0/FA16:0)+NH4	10107.1662	(7838.73, 12375.6)	15723.2719	(11748.62, 19697.92)	0.02253346	0.02797165	1.55565581
TAG(46:3/FA14:0)+NH4	9011.85835	(7210.92, 10812.8)	15878.5674	(10433.52, 21323.62)	0.02254515	0.02797165	1.76196371
TAG(47:2/FA18:1)+NH4	24496.9807	(19248.7, 29745.26)	37003.0457	(28348.1, 45658.0)	0.02396311	0.02965732	1.51051455
PE(O-18:0/22:5)-H	13531920.1	(12498263.25, 14565576.95)	15284484.2	(14267826.08, 16301142.39)	0.0244581	0.03019519	1.12951334
TAG(50:3/FA14:0)+NH4	7186.84488	(5247.09, 9126.6)	10940.7364	(8428.93, 13452.54)	0.02742969	0.03378041	1.52232816
TAG(53:3/FA18:2)+NH4	8849.03608	(6363.6, 11334.47)	13709.4289	(10556.78, 16862.08)	0.02754	0.03383293	1.54925675
PE(P-18:1/18:2)-H	52656286.9	(43392413.81, 61920159.91)	68155523.7	(58728579.6, 77582467.85)	0.02867999	0.03514704	1.29434732
TAG(54:2/FA18:1)+NH4	27710.984	(15218.56, 40203.41)	48016.4621	(35574.9, 60458.02)	0.03142704	0.03841937	1.73275919
TAG(56:4/FA20:4)+NH4	11291.1034	(9095.97, 13486.24)	17855.3551	(12582.55, 23128.16)	0.03175357	0.03872387	1.58136494
TAG(54:5/FA22:5)+NH4	10126.308	(7663.09, 12589.52)	14592.0198	(11573.55, 17610.49)	0.03217799	0.03914598	1.44100098
TAG(51:0/FA16:0)+NH4	10146.2522	(7844.04, 12448.46)	20586.0959	(11779.67, 29392.52)	0.03263618	0.03951872	2.02893595
TAG(46:2/FA18:2)+NH4	14350.671	(10618.16, 18083.19)	21607.269	(16407.99, 26806.54)	0.03264246	0.03951872	1.50566262
TAG(50:0/FA16:0)+NH4	18478.4938	(9662.24, 27294.75)	30975.9792	(24260.18, 37691.78)	0.03486263	0.04204538	1.67632598
TAG(52:2/FA18:2)+NH4	11256.9588	(8314.63, 14199.28)	16967.6658	(12844.54, 21090.79)	0.03489766	0.04204538	1.50730461
PE(P-18:2/20:4)-H	3814144.27	(3215487.66, 4412800.87)	4824055.78	(4153112.89, 5494998.67)	0.0355451	0.04272247	1.26478063
PE(P-18:0/20:1)-H	179140055	(160001512.88, 198278597.44)	210066447	(190216080.42, 229916814.3)	0.03578111	0.04290301	1.17263806
TAG(56:4/FA22:4)+NH4	10436.3293	(8268.53, 12604.13)	13722.1187	(11694.93, 15749.31)	0.03894635	0.04658654	1.31484148
TAG(50:4/FA16:0)+NH4	15433.4086	(11390.34, 19476.48)	21517.3514	(17658.48, 25376.23)	0.04137467	0.04937312	1.39420604
TAG(54:6/FA20:3)+NH4	10437.1065	(8760.5, 12113.71)	13997.1561	(11191.49, 16802.82)	0.04166291	0.0495987	1.34109546

TAG(56:5/FA22:5)+NH4	11664.1094	(8253.57, 15074.65)	17613.8633	(13449.23, 21778.49)	0.04229385	0.05023022	1.51009072
TAG(58:2/FA18:1)+NH4	12801.8436	(10510.54, 15093.14)	17834.9201	(13876.26, 21793.58)	0.04272871	0.05062644	1.39315248
TAG(50:5/FA20:4)+NH4	22494.6366	(17489.77, 27499.5)	32119.81	(24568.88, 39670.74)	0.04592248	0.0542819	1.42788748
PS(18:2/16:1)-H	2195801.8	(1865408.32, 2526195.27)	3014856.47	(2309961.55, 3719751.39)	0.0479473	0.05654163	1.37300938
PE(P-18:2/22:6)-H	600648.386	(508178.4, 693118.37)	761373.423	(639214.6, 883532.25)	0.04856057	0.05713008	1.2675859
PS(18:0/18:3)-H	559793.17	(487870.63, 631715.71)	686286.981	(587853.66, 784720.31)	0.05090892	0.05975225	1.22596526
LCER(d18:0/26:1)+H	14630.0778	(11202.8, 18057.36)	24369.9958	(15547.79, 33192.2)	0.05271877	0.06173158	1.66574615
PS(18:2/18:2)-H	614481.784	(513979.76, 714983.81)	491886.079	(426027.78, 557744.38)	0.05465216	0.06384598	-1.24923597
TAG(54:0/FA18:0)+NH4	15160.7671	(10905.96, 19415.57)	21737.7667	(16857.42, 26618.11)	0.05565895	0.06487057	1.43381707
TAG(50:1/FA16:1)+NH4	10587.908	(7466.23, 13709.59)	15399.9631	(11792.5, 19007.43)	0.05729225	0.06661889	1.45448592
TAG(44:2/FA18:2)+NH4	10807.698	(7088.36, 14527.04)	16384.1474	(12298.03, 20470.26)	0.05847391	0.06783516	1.51597014
PS(16:0/22:6)-H	885506.579	(622185.85, 1148827.3)	593243.903	(456480.07, 730007.74)	0.05887022	0.06813683	-1.4926518
PE(14:0/14:0)-H	312843.065	(270371.16, 355314.97)	364529.566	(332939.96, 396119.17)	0.06521698	0.07530829	1.16521543
PG(18:2/16:1)-H	4182986.8	(2360590.3, 6005383.3)	2310085.49	(1675820.13, 2944350.84)	0.06675846	0.07691066	-1.81074979
LPG(22:5)-H	117109.468	(98201.74, 136017.19)	158276.267	(120253.35, 196299.18)	0.06706966	0.07709156	1.35152409
TAG(52:1/FA16:0)+NH4	28524.4916	(10047.48, 47001.51)	50202.2412	(37265.82, 63138.66)	0.06932756	0.07950408	1.75996971
LPG(20:3)-H	4451920.48	(3711575.48, 5192265.49)	5629989.78	(4645851.6, 6614127.95)	0.0705669	0.08074016	1.26462047
DAG(18:2/20:3)+NH4	23190.6649	(17372.85, 29008.48)	31606.3435	(24929.79, 38282.89)	0.07233564	0.08257493	1.36289078
TAG(56:7/FA22:5)+NH4	13336.3191	(10585.99, 16086.65)	18537.6709	(13727.29, 23348.05)	0.07570928	0.08622924	1.39001405
TAG(54:7/FA20:4)+NH4	12887.8284	(9980.94, 15794.71)	17448.2462	(13248.5, 21647.99)	0.09033929	0.10244543	1.35385463
PS(18:1/18:2)-H	581464.874	(501259.43, 661670.32)	492963.939	(434691.99, 551235.88)	0.0903972	0.10244543	-1.17952821
TAG(56:5/FA20:2)+NH4	13490.001	(9959.57, 17020.44)	17591.9856	(14646.96, 20537.01)	0.09056176	0.10244543	1.30407592
TAG(49:0/FA16:0)+NH4	10334.0866	(5769.53, 14898.64)	15150.7022	(11841.24, 18460.16)	0.10444052	0.11787869	1.46609012

PE(P-18:0/22:6)-H	332576735	(297427220.9, 367726248.53)	376246824	(339012406.33, 413481241.1)	0.10500335	0.11824702	1.13130831
TAG(52:1/FA18:1)+NH4	20894.2176	(6873.87, 34914.56)	34315.8761	(26883.88, 41747.88)	0.10778132	0.1211026	1.64236234
PG(18:2/18:3)-H	131755.419	(92854.42, 170656.42)	95643.0271	(76749.9, 114536.16)	0.11214666	0.12572496	-1.37757474
TAG(54:6/FA18:2)+NH4	13741.6957	(10477.91, 17005.48)	19801.5084	(13078.88, 26524.14)	0.12246649	0.13698712	1.44097998
PS(14:0/22:4)-H	1224637.17	(1035181.15, 1414093.18)	1641477.81	(1091893.52, 2191062.09)	0.15993452	0.17849834	1.34037889
TAG(50:5/FA16:0)+NH4	15759.8601	(12434.98, 19084.74)	19662.9235	(15404.91, 23920.93)	0.17116525	0.19060718	1.24765851
TAG(54:5/FA20:5)+NH4	11129.8942	(7840.84, 14418.95)	15082.0033	(10608.95, 19555.06)	0.17320972	0.19245524	1.35508955
TAG(58:9/FA18:1)+NH4	16173.4001	(11707.32, 20639.48)	20114.5356	(16670.07, 23559.0)	0.17817527	0.19753356	1.24368008
LPG(22:6)-H	70357.8061	(57919.89, 82795.72)	58654.0447	(47028.62, 70279.47)	0.18794392	0.20790257	-1.19953886
PE(O-18:0/18:0)-H	1493405.39	(1157916.74, 1828894.05)	1766046.87	(1552818.72, 1979275.02)	0.18893186	0.20853406	1.18256361
PI(20:0/20:4)-H	364913.74	(329015.95, 400811.53)	326505.186	(282587.22, 370423.16)	0.19446282	0.21416611	-1.11763536
PE(18:2/20:5)-H	49513.3267	(38641.77, 60384.88)	41010.6317	(34387.4, 47633.86)	0.2078735	0.22843242	-1.20732904
TAG(56:2/FA16:0)+NH4	11553.4315	(9004.1, 14102.76)	13424.6981	(11963.75, 14885.65)	0.21472205	0.23544084	1.1619663
PG(14:1/14:1)-H	10986.7238	(6403.33, 15570.12)	15259.8999	(10373.78, 20146.02)	0.22089828	0.24168302	1.38893998
PE(18:2/20:3)-H	2028442.25	(1597103.8, 2459780.7)	2458449.09	(1937863.17, 2979035.0)	0.22217315	0.24254711	1.2119887
TAG(54:8/FA18:2)+NH4	12199.0984	(9270.5, 15127.69)	15104.8271	(11590.54, 18619.11)	0.22278054	0.24268032	1.23819209
PS(18:1/20:3)-H	222864.114	(192951.65, 252776.58)	198171.036	(171927.93, 224414.14)	0.23336734	0.25366015	-1.12460488
TAG(56:8/FA22:5)+NH4	10077.6164	(8261.31, 11893.92)	11956.6859	(9434.59, 14478.78)	0.24532649	0.26608079	1.18645972
TAG(54:1/FA18:1)+NH4	20127.2264	(8294.64, 31959.81)	27980.2307	(21488.76, 34471.71)	0.26311989	0.28476178	1.39016823
TAG(52:1/FA18:0)+NH4	24166.4068	(3649.24, 44683.57)	36902.7994	(28958.31, 44847.28)	0.26552095	0.28673969	1.52702881
TAG(50:5/FA20:5)+NH4	16896.3483	(11979.14, 21813.56)	21091.0476	(15394.2, 26787.9)	0.28330905	0.30528992	1.2482607
TAG(50:5/FA14:0)+NH4	9411.38048	(6974.75, 11848.02)	11222.7885	(9013.71, 13431.87)	0.28897694	0.31072789	1.19246996

TAG(52:0/FA18:0)+NH4	16976.8675	(5939.29, 28014.45)	24005.3282	(17364.0, 30646.65)	0.29341158	0.31481929	1.41400221
PS(14:0/20:1)-H	703706.526	(564542.88, 842870.18)	621659.756	(548669.82, 694649.69)	0.31432709	0.33653864	-1.13198019
PE(14:0/16:1)-H	1187820	(1029850.09, 1345789.92)	1295004.29	(1160312.97, 1429695.61)	0.31964533	0.34143676	1.09023613
TAG(56:6/FA22:6)+NH4	17495.5404	(9151.61, 25839.47)	22455.5018	(17669.47, 27241.53)	0.32026768	0.34143676	1.28349861
PS(18:1/20:1)-H	161288.208	(136755.54, 185820.87)	191328.039	(137756.35, 244899.73)	0.34140755	0.36319952	1.18624939
TAG(57:3/FA18:2)+NH4	11988.8025	(9945.95, 14031.66)	13606.9293	(10489.31, 16724.55)	0.3960213	0.42040478	1.13496985
PE(P-18:0/18:3)-H	582894.376	(413493.9, 752294.85)	682081.863	(533689.88, 830473.85)	0.40715581	0.43130912	1.17016374
PS(14:0/20:3)-H	154543.107	(115601.07, 193485.14)	178059.849	(135059.8, 221059.9)	0.43532423	0.4601736	1.15216947
PE(18:2/20:4)-H	3579906.9	(3103048.58, 4056765.22)	3834739.78	(3273750.48, 4395729.08)	0.50273274	0.5303088	1.07118422
TAG(50:5/FA18:3)+NH4	23434.9629	(15278.39, 31591.54)	27236.9606	(19266.07, 35207.85)	0.51846687	0.5457546	1.16223613
PS(18:1/20:5)-H	501220.44	(420034.19, 582406.69)	469422.634	(407236.96, 531608.31)	0.54682808	0.57439924	-1.06773812
PE(14:0/20:5)-H	11883.5003	(8559.14, 15207.86)	10735.195	(9007.88, 12462.51)	0.56941608	0.5968722	-1.10696642
TAG(46:0/FA16:0)+NH4	16769.7147	(12042.27, 21497.16)	18993.8707	(12811.64, 25176.1)	0.57954279	0.60621631	1.13262933
PE(P-18:2/18:2)-H	579188.639	(473431.07, 684946.21)	533858.708	(423119.73, 644597.68)	0.5816123	0.60711096	-1.08490998
PS(16:0/20:5)-H	6863045.29	(5696490.18, 8029600.4)	7283175.77	(6361401.24, 8204950.3)	0.58378567	0.60811007	1.06121634
PE(18:2/22:5)-H	656091.321	(475580.27, 836602.37)	594520.999	(445992.26, 743049.73)	0.61004935	0.63414693	-1.10356291
PS(16:0/20:4)-H	2587354.45	(2188602.36, 2986106.54)	2751902.68	(2260779.8, 3243025.57)	0.61391507	0.63684136	1.0635971
TAG(57:10/FA22:6)+NH4	15311.9252	(11769.05, 18854.8)	16402.1293	(13846.68, 18957.58)	0.62512976	0.64713226	1.07119967
PS(18:0/20:5)-H	5160404.29	(4386254.49, 5934554.08)	5433059.46	(4622637.61, 6243481.32)	0.63694169	0.65799762	1.05283601
PS(18:0/16:1)-H	5030752.19	(4331619.19, 5729885.19)	5288121.63	(4302575.95, 6273667.32)	0.6793085	0.70016818	1.05115924
PE(14:0/20:1)-H	1398200.61	(1178192.41, 1618208.8)	1330967.98	(1102784.97, 1559150.99)	0.68056347	0.70016818	-1.05051408
PS(16:0/20:2)-H	252651.115	(162800.84, 342501.39)	277308.444	(201239.25, 353377.64)	0.68345009	0.70169413	1.09759438

TAG(58:10/FA18:2)+NH4	13746.3828	(7784.42, 19708.34)	15057.8518	(12282.77, 17832.94)	0.69864981	0.71582972	1.09540466
LPG(20:0)-H	989891.501	(594440.49, 1385342.51)	1066414.36	(774697.36, 1358131.36)	0.76230979	0.77945786	1.07730429
PS(18:2/20:5)-H	317223.665	(266006.73, 368440.6)	305348.974	(246782.72, 363915.23)	0.76688953	0.78254033	-1.03888892
PE(P-18:1/18:3)-H	872029.024	(410950.8, 1333107.25)	966630.509	(522633.53, 1410627.49)	0.77405959	0.78824805	1.10848433
PE(O-18:0/20:2)-H	3638017	(2340902.5, 4935131.49)	3399536.06	(2312185.09, 4486887.02)	0.78431929	0.79707245	-1.07015102
TAG(54:1/FA18:0)+NH4	30564.9106	(1358.72, 59771.1)	34018.5148	(25789.21, 42247.82)	0.8201215	0.83176623	1.11299245
LPG(18:3)-H	56830.5916	(34036.21, 79624.97)	53786.4712	(40153.53, 67419.41)	0.8237822	0.83378765	-1.0565964
PS(16:0/22:5)-H	4053765.21	(3330389.51, 4777140.92)	3938038.42	(3192243.99, 4683832.86)	0.82866248	0.83703281	-1.02938691
PS(18:2/20:1)-H	285263.8	(237571.97, 332955.63)	293744.379	(227895.96, 359592.8)	0.84393779	0.85074374	1.0297289
PS(18:1/20:4)-H	822486.192	(729945.8, 915026.59)	830760.351	(780446.06, 881074.64)	0.87867137	0.88397522	1.01005994
LPG(17:1)-Hstd.IS	489152.269	(382069.77, 596234.76)	497254.382	(397675.48, 596833.28)	0.91424406	0.91791572	1.01656358
PG(18:1/22:5)-H	1074514.98	(959104.39, 1189925.58)	1070004.65	(891690.14, 1248319.16)	0.96756192	0.96950092	-1.00421525
PS(18:1/20:2)-H	61788.0105	(37695.52, 85880.5)	61377.2904	(45851.22, 76903.36)	0.97719913	0.97719913	-1.00669173

Table S6: Optimized model parameters for each machine learning algorithm evaluated for prediction of cognitively healthy controls as compare to Mild-AD.

	accuracy	AUC	sensitivity	specificity	balanced accuracy	f1-score pos	precision
**logistic reg**	0.84	91.00	0.87	0.81	0.84	0.84	0.81
**discr linear**	0.74	84.00	0.87	0.63	0.75	0.76	0.68
**discr quad**	0.74	82.00	0.87	0.63	0.75	0.76	0.68
**gaussian nb**	0.84	85.00	0.87	0.81	0.84	0.84	0.81
**svm linear**	0.77	83.00	0.80	0.75	0.78	0.77	0.75
**svm kernel**	0.74	82.00	0.87	0.63	0.75	0.76	0.68
**knn**	0.71	81.00	0.73	0.69	0.71	0.71	0.69
**decision tree**	0.84	90.00	0.87	0.81	0.84	0.84	0.81
**random forest**	0.84	91.00	0.87	0.81	0.84	0.84	0.81
**gbm**	0.90	92.00	1.00	0.81	0.91	0.91	0.83
**xgboost**	0.90	91.00	0.93	0.88	0.90	0.90	0.88

Table S7: Optimized model parameters for each machine learning algorithm evaluated for prediction of Mild-AD as compare to AD.

	accuracy	AUC	sensitivity	specificity	balanced accuracy	f1-score pos	precision
**logistic reg**	0.87	92.00	0.94	0.80	0.87	0.88	0.83
**discr linear**	0.71	88.00	0.75	0.67	0.71	0.73	0.71
**discr quad**	0.71	88.00	0.75	0.67	0.71	0.73	0.71
**gaussian nb**	0.77	90.00	0.81	0.73	0.77	0.79	0.76
**svm linear**	0.84	91.00	0.94	0.73	0.84	0.86	0.79
**svm kernel**	0.84	90.00	0.94	0.73	0.84	0.86	0.79
**knn**	0.71	86.00	0.75	0.67	0.71	0.73	0.71
**decision tree**	0.87	93.00	0.94	0.80	0.87	0.88	0.83
**random forest**	0.90	94.00	1.00	0.80	0.90	0.91	0.84
**gbm**	0.90	92.00	0.94	0.87	0.90	0.91	0.88
**xgboost**	0.90	94.00	0.94	0.88	0.90	0.91	0.88

Table S8: Optimized model parameters for each machine learning algorithm evaluated for prediction of HC as compare to AD.

	accuracy	AUC	sensitivity	specificity	balanced accuracy	f1-score pos	precision
**logistic reg**	0.84	99.00	0.94	0.75	0.84	0.86	0.79
**discr linear**	0.78	95.00	0.81	0.75	0.78	0.79	0.76
**discr quad**	0.78	93.00	0.81	0.75	0.78	0.79	0.76
**gaussian nb**	0.84	95.00	0.94	0.75	0.84	0.86	0.79
**svm linear**	0.84	96.00	0.94	0.75	0.84	0.86	0.79
**svm kernel**	0.88	95.00	0.94	0.81	0.88	0.88	0.83
**knn**	0.78	93.00	0.81	0.75	0.78	0.79	0.76
**decision tree**	0.84	90.00	0.94	0.75	0.84	0.86	0.79
**random forest**	0.91	99.00	0.95	0.89	0.91	0.91	0.88
**gbm**	0.91	88.00	0.94	0.90	0.91	0.91	0.88
**xgboost**	0.91	89.00	0.93	0.86	0.91	0.91	0.88

Figure S1: Heatmap of thirteen class of lipids selected during Random Forest classification, following recursive feature elimination, which were consistently above the level of quantification (LOQ). AD, Alzheimer's disease; MAD, Mild-AD (Mild Alzheimer's disease).

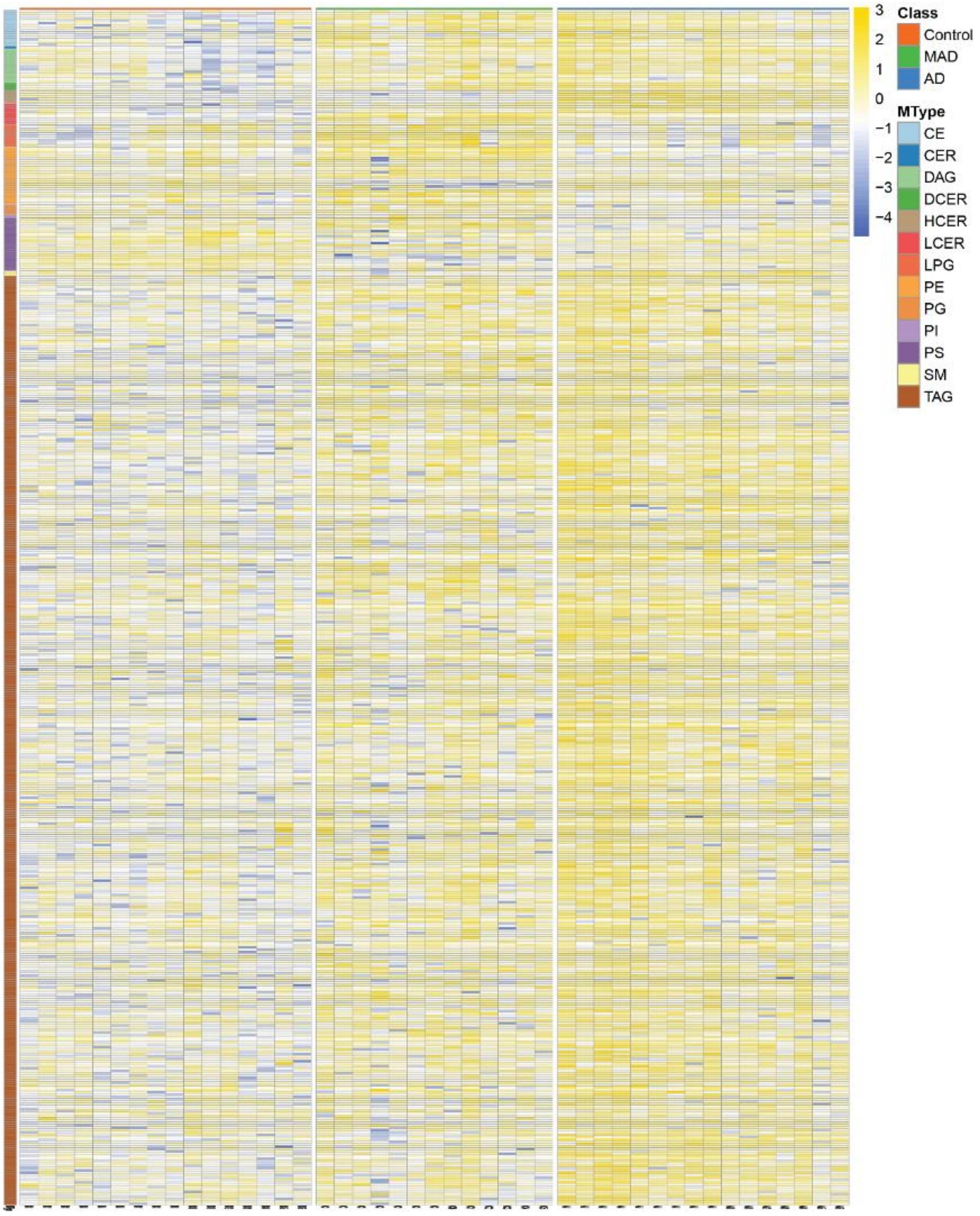

Supplement: Supplementary file 1 [file cells-10-02591-s001.zip › cells-1391474-supplementary.pdf]
